# Exposure of laboratory animals to small air ions: a systematic review of biological and behavioral studies

**DOI:** 10.1186/s12938-018-0499-z

**Published:** 2018-06-05

**Authors:** William H. Bailey, Amy Lavin Williams, Megan Jeanne Leonhard

**Affiliations:** 1 Health Sciences Center, Exponent, 17000 Science Drive, Suite 200, Bowie, MD 20715 USA; 2 Health Sciences Center, Exponent, Alexandria, VA 22314 USA; 3 Health Sciences Center, Exponent, Bellevue, WA 98007 USA

**Keywords:** Air ions, Atmospheric ions, Corona ions, Positive ion, Negative ion, Ionization, Space charge, Animal, Toxicity, ARRIVE

## Abstract

**Background:**

Air ions are molecules of air that have become ionized—that is, they have either lost or gained an electrical charge. Past speculation has suggested that exposure to positive air ions may be harmful to one’s health, while exposure to negative air ions may be associated with beneficial health effects. Air ions arise from natural sources as well as direct-current transmission lines and commercial ionizers. Several recent clinical studies have suggested therapeutic effects of air ions on various types of depression at exposure levels 10- to 1000-fold higher than most previous human studies. The aim of this study was to assess the evidence from studies of laboratory animals for beneficial or adverse effects of air ions on health.

**Methods:**

Sixty-two studies (1935–2015) in nine topics areas were evaluated for quality and potential systematic bias by ARRIVE guidelines. Standardized mean differences or proportional differences between exposed and control groups were computed for 44 studies to quantitatively assess the strength of the evidence for exposure-related effects.

**Results:**

Many of the studies were conducted before 1990 and exhibited various reporting and methodological deficiencies, including small sample size, failure to control for the influence of potential confounding variables, lack of randomized assignment to treatment groups and blinded analyses, and statistical errors relating to treating group-exposed animals as individuals. The highest quality studies consistently reported no effects of exposure on any of the endpoints examined. There were no evident dose–response relationships within or across studies.

**Conclusions:**

Experimental studies of laboratory animals exposed to positive and negative air ions for minutes to years over a five-log unit range of intensities did not suggest any consistent or reliable effects on measures of behavior, learning and memory, neurotransmitters, tracheal function, respiratory infection, cardiovascular function, reproduction and growth, carcinogenesis, or other health endpoints. These data do not provide evidence of adverse or beneficial effects of air ion exposure on health, and did not suggest any biological mechanism of interaction, except perhaps for mechanosensory stimulation of body surfaces by static electric fields at high air ion concentrations.

**Electronic supplementary material:**

The online version of this article (10.1186/s12938-018-0499-z) contains supplementary material, which is available to authorized users.

## Background

Small air ions are clusters of molecules of air that have become ionized—that is, they have either lost or gained an electron, and thus, carry an electrical charge of negative or positive polarity. Small air ions are a natural phenomenon generated by various atmospheric and weather events (e.g., rain, wind, snow, lightning), waterfalls, the rolling of ocean waves, combustion, the natural radioactivity produced by assorted geologic formations, and cosmic radiation [1].

Other sources include direct-current electric transmission lines. Although alternating-current transmission lines also produce air ions, the levels away from the conductors at ground level are very low because most air ions are attracted back to the conductor and neutralized with each alternating cycle. The generation of air ions from transmission lines occurs during corona; defined as a “*luminous discharge due to ionization of the air surrounding an electrode {power line conductor} caused by a voltage gradient {electric field} exceeding a certain critical value*” [2]. Corona discharge may also produce small amounts of ozone, and audible noise. Similarly, corona generated by air ionizers sold commercially for in-home use as air purifiers adds electric charge to the air to precipitate particles.

The existence of small air ions as clusters of gas molecules is determined by one or more attached electrical charges. The removal of an electron from an atom or gas molecule creates an elemental ion that immediately attracts a cluster of water and gas molecules. When the charge is neutralized, by recombination with ions or molecules of opposite charge or transferred to a larger particle or aerosol, the air ions cease to exist as such and are just air molecules.

Since air ions carry electric charge, the behavior of air ions is determined not only by mechanical forces (e.g., diffusion, air currents, etc.) as are other gas molecules, but also by electric gradients in the air that exert force on the ions to move along potential gradients. Small air ions are clusters of a few molecules held together by the electric charge with radii less than 0.001 µm and mobility in the range of 10^−5^ m^2^/V s to 2 × 10^−4^ m^2^/V s [2]. In contrast, large air ions are particles or nuclei that have electrical mobilities about 500-fold lower, and unlike small air ions, these nuclei persist in a neutral state when uncharged [1].

Measurements of the chemical species of positive and negative air ions around direct-current transmission lines by tandem mass spectroscopic analysis confirm that their composition is like naturally occurring air ions measured away from transmission lines [3, 4]. As described above, factors including the concentrations of air ions and aerosols, and the presence of electric fields can result in small air ions with short lifetimes (seconds) or somewhat longer lifetimes (minutes). Therefore, while the concentrations of air ions studied in a natural or experimental environment may be similar, the lifetimes of these ions may not be the same. A point to note is that air ions generated by sources other than corona discharge may not be accompanied by as large electric fields, and exposures to ozone, audible noise, and light can be expected to be minimal.

**Fig. 1:**
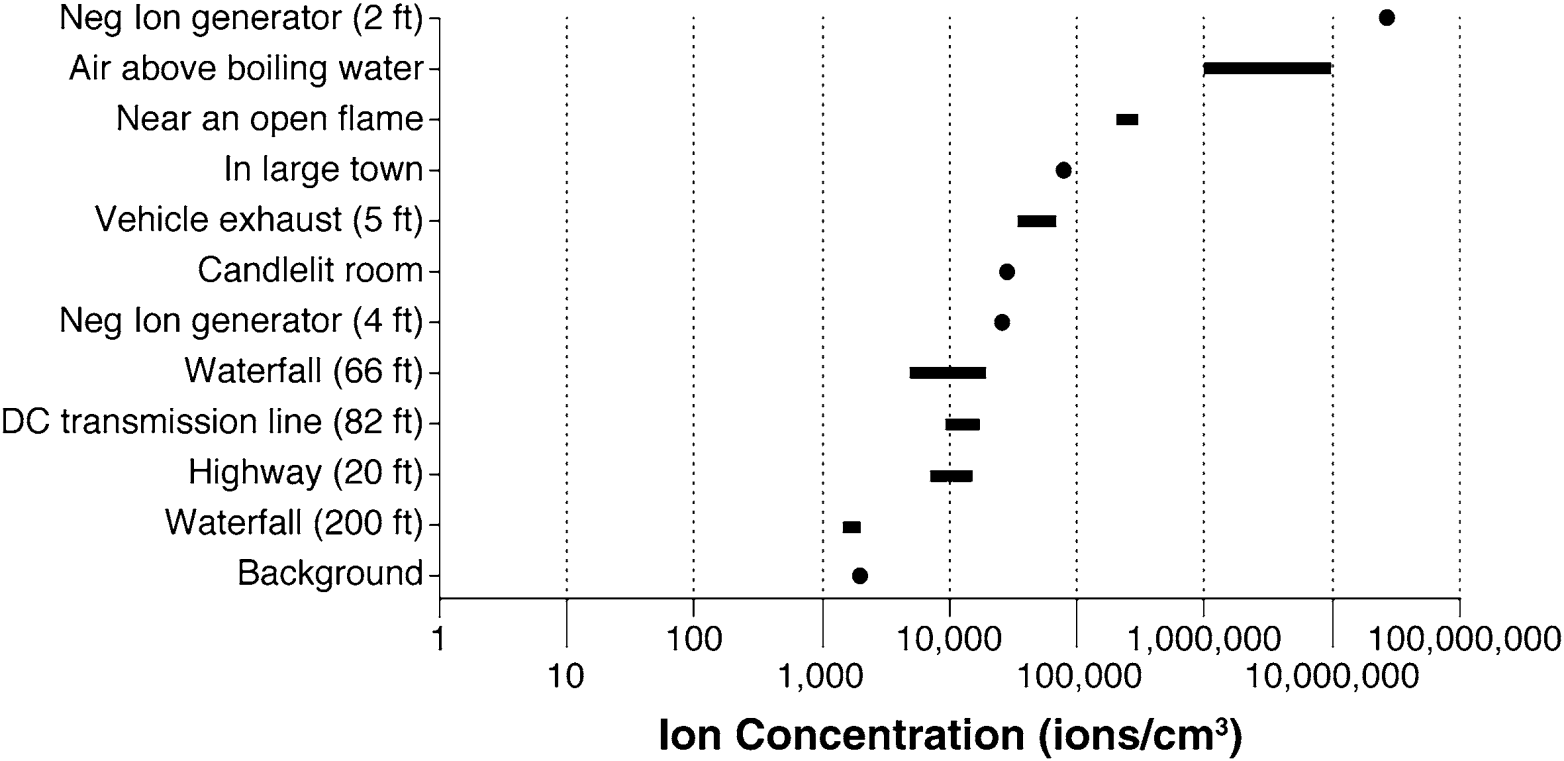
Total density of air ions measured in a variety of locations and near sources. Data sources [5–10].  See [6] for conversion of ion levels in Terman and Terman [5]

Measurements of air ions, the exposure of interest in this review, are not widely reported, but Fig. 1 shows measurements of the concentration of air ions per cm^3^ of air at a variety of locations, including those at identified distances from specific sources. The highest air ion concentration in Fig. 1 was reported in a clinical study of patients treated for seasonal affective disorder (winter depression) with negatively-charged air ions [5].

Since the discovery of electrical charges on molecules in the air in 1899 [11] there has been speculation suggesting that exposure to positive air ions in the natural environment or from experimental sources may be adverse to one’s health, while exposure to negative air ions may be associated with beneficial health effects [12, 13]. Most of the investigations of potential biological responses to air ions in human studies have focused on the respiratory system and on mood and behavior. Two comprehensive meta-analytic reviews summarized this research. Alexander et al. [14] found no consistent evidence for an effect of positive or negative air ions on respiratory function. Similarly, Perez et al. [6] reported no experimental support for an effect of positive or negative air ions on various psychological parameters related to mood or emotional state. The exception was an association between exposure and high concentrations of negative air ions and lower depressive scores of participants in a few recent clinical studies [5, 15, 16]. Perez et al. [6] recommended more research to address the biological plausibility of this finding.

Examination of experimental studies in which animals were exposed to air ions is warranted to better assess the general plausibility of claims for air ion effects and, in light of recent tests of negative air ions, as a therapeutic treatment for various types of depression. These human studies were conducted at exposure levels (> 2 × 10^5^–2 × 10^7^ ions/cm^3^) [5, 15, 16] that are 10- to 1000-fold higher than most previous human studies. Hence, there also is a need to assess the potential for toxic effects to humans based on the results of experimental animal studies.

Data from experimental animal studies are important for addressing potential health risks of air ions to humans for several reasons. First, in many of these studies, laboratory animals were exposed to air ions at much higher levels and for a longer duration (particularly in terms of an animal’s lifespan) than in most of the human studies. Second, portions of the respiratory system of laboratory animals may incur greater exposure than human subjects even at the same air ion concentration. This occurs in the tracheobronchial region of the respiratory tract of the rat, where the inhaled deposited dose of aerosol particles smaller than 2 µm is 5–14 times greater than that of a human (regional deposited dose ratio), which may have implications for exposure to air ions as well [17]. In addition, the fraction of air ions available for inhalation is reduced in the presence of static electric fields at the head [18]. Thus, since the local electric field at the head of a rodent is lower than at the head of an upright person [19], the opportunity for respiratory tract exposure may be greater for a rodent than a human when any static electric field is present. Finally, experimental animal studies have examined a wider range of behavioral and physiological measures than in human studies. Since there are substantial similarities between humans and other mammals in terms of how their physiological systems function, responses observed in laboratory animals are generally considered in the assessment of potential responses of humans, particularly regarding health and safety.

There are no current reviews of the animal literature on exposure to air ions. The most recent comprehensive evaluation was conducted in 1997 for the Oak Ridge National Laboratory [20], which addressed the available experimental animal studies of exposures as part of a generic environmental assessment of high-voltage direct-current transmission lines, including exposure to air ions. A few studies of indicators of the distribution of space charge around alternating-current transmission lines and mechanistic hypotheses were reviewed by the International Agency for Research on Cancer [21] and by the Advisory Group on Non-Ionizing Radiation on behalf of the National Radiation Protection Board of Great Britain [22]. Both reviews assessed the hypothesis that high-voltage-power lines might increase general exposure to charged aerosols and in turn increase the deposition of airborne pollutants on the skin and on airways inside the body, possibly adversely affecting health. Neither concluded that air ions would have any significant effect on the health of even the most exposed persons.

The objective of this review is to assess the potential biological effects of small air ions on laboratory animals to determine the biological plausibility of the wide range of weak and largely unconfirmed responses reported in human studies. This review evaluates the research literature published from 1935 to 2015 and includes studies in nine major topic areas of investigation. Information regarding experimental details, reported findings, including the strength of effects in exposed groups relative to control groups, and methodological strengths and weaknesses are provided.

## Methods

### Identification and selection of studies

Systematic searches of the literature were conducted to identify experimental animal studies of air ion exposure. The databases were: Medline (PubMed) bibliographic database (coverage 1946 to the present) [23]; the IEEE Xplore Digital Library (coverage 1872 to the present) [24]; and EMF-Portal, a specialized database of biological and health studies of electric and magnetic fields hosted by RWTH Aachen University since 2002 [25]. These databases were searched for studies conducted in laboratory animals prior to March 2017 using the search terms space charge, atmospheric ion, charged aerosol, air ionization, corona ion, electromagnetic phenomenon, and animals. No relevant studies published after 2015 were found.  In addition, references cited in previous structured reviews [20, 26] and in the reference lists of studies retrieved were reviewed, and studies not retrieved by the systematic literature searches of databases that met selection criteria were added.

In this review, articles were restricted to those published in English that reported primary data from investigations conducted in experimental animals under controlled laboratory conditions. Secondary reports or reviews were excluded. Studies conducted in humans or in vitro cellular systems also were excluded.

### Data extraction and statistical analysis

Information on the animal subjects, study design, ion concentration and polarity, and exposure duration was extracted by reviewers (ALW, WHB) and summarized into nine subject matter categories: behavior; learning and memory; serotonin and other neurotransmitters; tracheal function; respiratory infection; cardiovascular function; reproduction and growth; carcinogenesis; and other health endpoints. Quantitative data were extracted from 44 studies by an independent third reviewer (MJL). Because of the disparate nature of the outcome measures within each of the categories of studies, meta-analyses of the data were not considered appropriate nor informative. Rather, the standardized mean difference (SMD)—the difference in group means between exposed and unexposed groups divided by the pooled standardized deviation—was computed with Hedges’ g formula with correction for small sample bias [27] and displayed in forest plots. Since the endpoints reported in the air ion literature vary widely, expressing the results of the experimental tests in a uniform way allowed for more direct comparison of results and an appreciation of the relative magnitude of the reported effects. The data reported in studies of respiratory infection were expressed as the proportional difference (PD) in the mortality of treated and untreated groups to the infectious agents. The PD represents the difference between the reported probability of an event in the exposed group and the reported probability of an event in the unexposed group. SMD and PD values > 0 show the extent to which the mean of the animals exposed to air ions exceeded the mean response of control animals. SMD and PD values < 0 show the converse.

In addition, to assess the strength-of-evidence from the studies, SMDs and PDs were categorized as providing moderate and strong evidence for treatment effects of air ions. In Bayesian terms, the strength of the evidence is calculated as the logarithm of the likelihood ratio of the probability of the result given the null hypothesis and the probability of the result given the alternative hypothesis. Goodman [28] has shown that study results with a minimum Bayes factor of 1/28 or 1/216 can be identified by calculating *p* values = 0.01 and 0.001, respectively. So, following Goodman, if we assume an a priori probability of 75% for the null hypothesis, then the posterior probability of the null hypothesis being true for *p* = 0.01 is reduced to no less than 10%. A greater strength of the evidence is demonstrated for an effect with a *p* value = 0.001, because an a priori probability of the null hypothesis of 75% is reduced to no less than 1%.

Significance levels of *p* < 0.01 and *p* < 0.001 were selected to screen for modest and strong effects of air ions [29]. These significance levels were selected post hoc to focus the evaluation and discussion on the small fraction of the large number of endpoints that were included in the review for which there was quantitative evidence for an effect. The application of the *p* < 0.001 level was further justified given that most of the studies had a small number of subjects per group (n ≤ 12) and therefore a low statistical power to detect effects. Based on assessments of animal studies, underpowered studies also more frequently test unlikely or novel hypotheses, offer weak protection against false positive findings, report multiple tests of significance without corrections, and are more likely to report a greater over dispersion of values because data from subjects are not independent [30]. Underpowered studies are also associated with overstated findings [31]. In the interest of transparency for the reader, SMDs and PDs that were calculated to meet *p* < 0.01 and *p *< 0.001 are identified in the forest plots. When calculating these p levels, reported sample sizes were used whether the animals were exposed individually or in groups.

In the body of research reviewed here almost none of the studies examined had an expected power approaching 80% based on just a priori considerations of sample size. For example, a two-tailed test capable a priori of detecting a difference of an SMD = 0.8 between two independent groups at *p* = 0.05, 0.01, and 0.001 would call for 21, 33, and 51 animals, respectively, in each group (G*Power software version 3.1.9.2) [32].

The validity of the standard statistical analyses used to assess the likelihood of a result depends upon the assumption that the observations of the subjects are independent. In a good number of the studies reviewed, this assumption was violated because the subjects received treatment exposures in groups. This means that the observed behavioral and biological responses of the animals cannot be considered wholly independent, which is a requirement of statistical models used in data analyses. In such studies, the exposed groups should be considered as the experimental unit in statistical analyses, not the individual animal. Where this design and analysis error occurs, the variability of the within-group measurements is underestimated, and therefore, the magnitude of the SMDs and calculated *p*-values may be exaggerated, which occurs when the individual and not the litter is used as the unit of analysis in studies of reproductive effects [33–37]. A large majority of the studies reviewed here were found to have little apparent power because the animals were exposed in groups and so a higher criterion to assess the statistical strength of evidence should be required.

### Assessment of study quality and systematic bias

Certain aspects of experimental research studies are important indicators of study quality. Two authors (WHB and ALW) reviewed key indicators of appropriate experimental design and control for potential systematic bias in each study, based on general criteria recommended in the Animals in Research: Reporting in Vivo Experiments (ARRIVE) guidelines for reporting animal laboratory research by study investigators and tailored to address the issues posed by the research literature reviewed [38]. The need for evaluation of study design and risk of bias features cannot be overstated since fewer than 50% of randomly sampled in vivo life-science publications reported on such measures that reduce risk of bias [39]. The reviewers rated studies as Yes or No as to the use of sham controls, blinding, randomization of subjects, single subjects as the unit of exposure and analysis, and control of potential confounding factors (electric field, ozone, noise, and light) as key indicators of quality to protect against potential systematic bias. Such characteristics are regarded as indicators of study quality because they reduce the risk of systematic error or bias [30, 38–42]. Differences in ratings were resolved by consultation between WHB and ALW. The relationship between the ratings of eight quality indicators (percent of possible) achieved (not all indicators were applicable to all studies) and the statistical strength of the evidence (percent of endpoint tests with differences at *p* < 0.01 and *p* < 0.001) were compared in scatter plots.

#### Sham controls

Even though experimental studies carried out in laboratories should be carried out under conditions where the only difference between the environment of the control and treatment groups is the exposure of interest, rarely are all potentially relevant aspects of the environment of the study subjects well controlled and described. In particular, if controls are to be properly compared to exposed subjects, their history should be as similar as possible and so sham-exposed controls are best, particularly when most of the endpoints of interest are neurobiological and might be affected by differential handling or environmental conditions. The use of sham-exposed controls was noted as a quality indicator in the studies reviewed.

#### Randomization

Randomization of subjects to treatment and control groups is one of the key concepts of ARRIVE guidelines. Since the history of the pre-experimental conditions of treated and control animals may differ (e.g., light, availability of food, prevalence of viral and bacterial infections, and other factors), allocating all animals from one cage to control conditions and all animals from another cage to a treatment group may lead to differences between these groups that reflect conditions extraneous to the experimental variable of interest. Hence, all studies included in this review of animal research were evaluated for evidence that the investigators selected and assigned subjects to control and treated groups by a randomization process that could minimize the potential for systematic bias to affect the outcome of the experiment.

#### Blinding

ARRIVE guidelines dictate that since knowledge of the exposure history of study subjects can be another source of systematic bias, the experiment must be designed so that the investigators are blind to the subjects’ exposure history when collecting data and conducting preliminary analyses. Knowledge of the exposure history of the subject is recognized to affect the investigator’s attitude, perception, and handling of the animals during experiments [43, 44]. Studies that report methods to blind the investigator to information about the test animals (including their experimental groups) until the conclusion of the final data analysis to safeguard against this source of bias are judged to be of higher quality than those that do not report such methods.

#### Confounding

Examples of exposures that may accompany the experimental generation of air ions that might elicit biological and behavioral responses of the animals independent of any effect of air ions alone include the generation of unwanted static electric fields, ozone, high frequency noise, and light. Hence, the reviewers scrutinized each study to determine if the investigators measured or controlled these confounding factors. These confounding exposures are most likely to occur when air ions are generated by corona discharge. A review [45] and scientific review panels such as those assembled by the US National Research Council [46] have long recognized the importance of identifying and controlling confounding factors in biological studies of electrical phenomena, but this has been invariably ignored by many study investigators.

#### Dose–response relationship

Generally, a response that is causally related to treatment will increase as the treatment intensifies (i.e., as the dose increases) or as the duration of exposure increases. In contrast, responses that occur in an experiment without a clear relationship to the treatment may be due to the influence of other extraneous factors in the experiment or result from the inherent variability of that response. Since a greater response to exposure with increasing intensity or duration of exposure (i.e., the dose–response relationship) can be a strong indicator that the exposure of interest is causal, attention was focused on this aspect of the studies reviewed. Dose–response relationships for effects related to the concentration of air ions were evaluated by plotting the SMDs reported within each topic category.

## Results

A total of 62 studies published from 1935 to 2015 were retrieved that met the screening criteria, and no study was excluded based on its quality or results.

### Summary tables of identified studies and findings

Details regarding the species, strain, sex, number of animals per group, air ion concentration and exposure duration, source of ion generation, use of sham controls, control for confounders, random allocation of subjects to treatment groups and blinding to prevent experimental bias, and findings are summarized in Additional file 1: Tables S1–S9.

### Graphic displays of standardized mean differences

The differences between the mean response of animals exposed to air ions and controls, expressed in units of SMD or PD, are illustrated in Additional file 2: Figures S1–S8. In each figure, the SMD or PD values are shown by markers opposite each response reported in the paper. The SMDs and PDs are identified separately for exposure to negative and positive air ions. An SMD or PD equal to 0 indicates that the mean responses of the exposed and control groups do not differ. If the SMD or PD has a value greater than 0, then the response of the exposed group is greater than the control group; an SMD or PD less than 0 indicates that the response of the exposed group is less than the response of the control group. The calculated confidence interval (CI) about the mean SMD or PD values is shown for an assumed *p* < 0.05. A wide CI reflects considerable imprecision in the estimated mean value; conversely, a narrow CI reflects greater precision.

A difference between treatment groups, however, at the *p* < 0.05 level provides little protection against incorrectly rejecting the null hypothesis [28], so those SMD or PD markers where the differences were less than *p* < 0.01 were identified by blue color coding to indicate moderate statistical evidence and orange color coding to denote *p* < 0.001 for stronger statistical evidence that exposed and control group mean values differed. As discussed subsequently, if the animals were exposed as a group, but the analysis considered the individual animal as the experiment unit, then the statistical analysis will be invalid and the n per group reduces to 1.

In some studies, the publications provided insufficient information to determine the SMDs or PDs. The results of the studies not summarized in SMDs and PDs in Additional file 2 are discussed in less detail below, but also are summarized in Additional file 1. The studies for which SMDs or PDs could not be extracted also are identified in notes to figures in Additional file 2.

### Behavioral measures

**Fig. 2:**
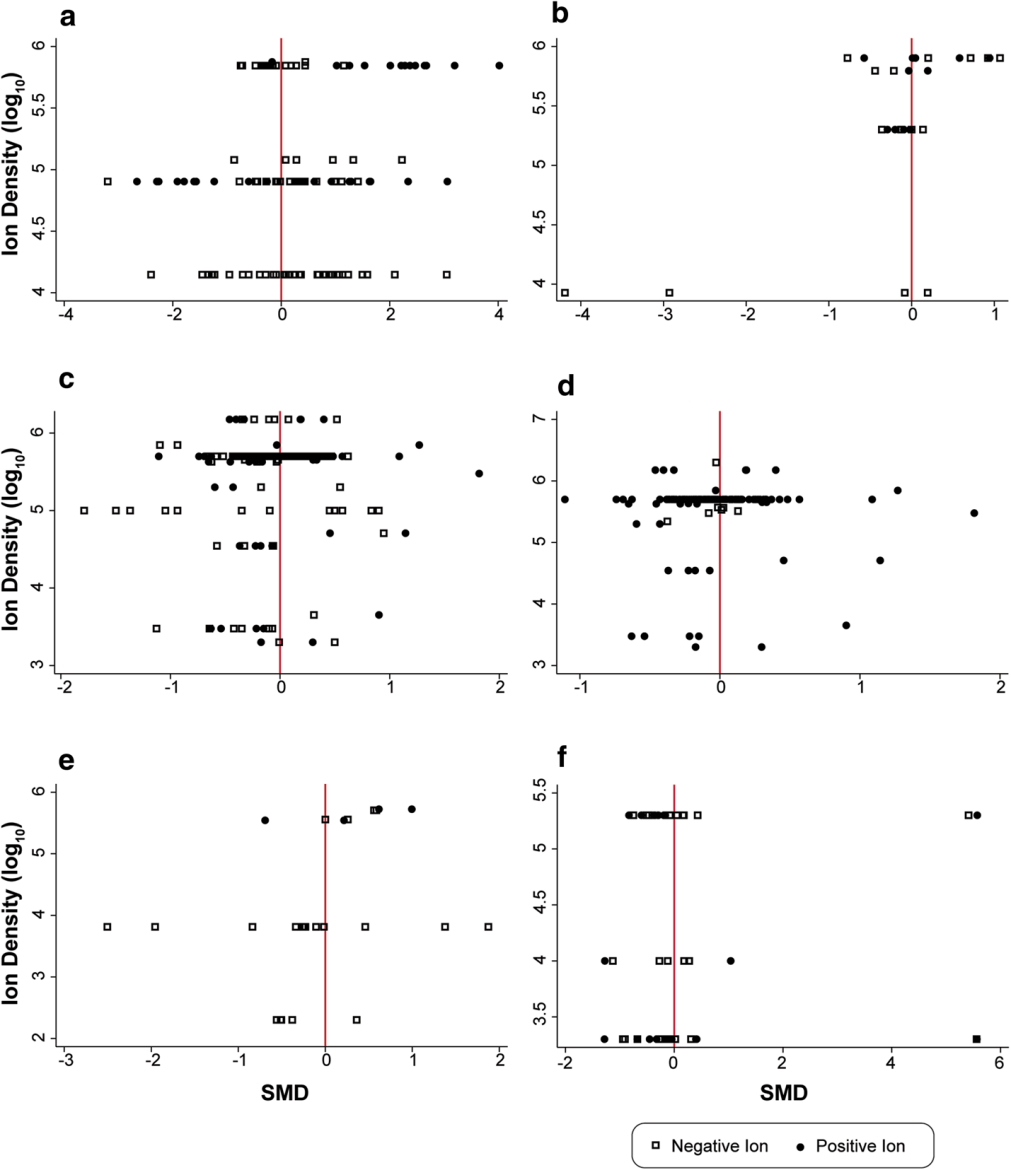
Dose–response plots for negative and positive ion exposures: **a** behavior; **b** learning and memory; **c** serotonin and other neurotransmitters; **d** respiratory infection; **e** cardiovascular function; **f** reproduction and growth

Thirteen studies were reviewed in which rats, mice (one study), and hamsters (one study) were exposed to positive or negative air ions over periods from 10 min to 300 days. Additional file 2: Figure S1 summarizes the SMDs for 118 tests performed in 10 of these studies, including wheel running [47, 48]; brain electrical activity [49, 50], multiple measures of spontaneous behavior [49, 51, 52], responses to aversive stimuli [53, 54], and altered sleep patterns [55]. About 10% of the 118 SMDs summarized in Additional file 2: Figure S1 exhibited strong support for a difference between animals exposed to positive or negative air ions and controls at *p* < 0.001. Because multiple measurements (e.g., amplitude of the electroencephalogram [EEG]), frequency of EEG, and behavior were reported at more than one time point on each animal in most studies, the data were highly correlated, and this was not considered in the statistical analyses. There was no apparent consistency in the responses within or between studies. Exposure to higher levels of air ions did not produce greater responses on behavioral measures (Fig. 2a).

The findings of other studies for which SMDs were not calculated included increased motor activity with either polarity of air ion exposure [56], no effect of positive or negative air ion exposure on spontaneous motor activity [57], reduced pain response to aversive stimulation following positive air ion exposure [58], reporting the same data as the authors’ previous studies [53, 54] discussed above, no evidence for avoidance of high air ion exposure of either polarity [59], and prevention of an acute stress response by exposure of immobilized rats to negative air ions [60]. While some of the studies reported that air ions affect some measured aspects of rodent behavior, none of the tests employed were sufficiently similar in content or validity to standard tests used to screen for antidepressant activity to constitute a test for this specific type of response [61].

### Learning and memory

Nine studies evaluated effects of air ion exposure on learning and memory in rats and mice. Only three studies permitted calculation of SMDs (Additional file 2: Figure S2). Jordan and Sokoloff [62] reported an improbably large reduction in errors on the water maze performance of older, but not younger, rats when exposed to negative air ions in groups of five (SMD = 2.9 and 4.2; *p* < 0.001). Another study described the results of two different experiments that examined the effects of short- and long-term exposure on 24 measures of learning and memory performance [49]. Some of the same data were reported by these authors in an earlier study [63]. A third study [64] tested whether negative and positive air ions (and concurrent static electric-field exposures) administered after drinking sweetened water would suppress later drinking of sweetened water as has been observed for other stimuli that produce gastric distress or other adverse effects. Neither study suggested any effect of air ion exposure, as the SMDs all cluster around zero. Exposure to higher levels of air ions did not produce greater responses on learning and memory tasks (Fig. 2b). Another six studies for which SMDs were not extracted reported no or inconsistent effects on learning of various tasks [65–70].

### Serotonin, other neurotransmitters, and brain development

One of the main foci of air ion research has been on the involvement of neurotransmitters in neurobehavioral functions postulated to be affected by air ions [13]. The SMDs of tests reported from 13 studies are summarized in Additional file 2: Figure S3. The laboratory of Alfred Krueger studied the effect of positive and negative air ions on the levels of serotonin, also known as 5-hydroxytryptamine (i.e., 5-HT), in multiple tissues. In the initial experiment, groups of four mice were exposed to negative air ions for 14 h or continuously exposed for an unspecified period. Improbably large reductions in the serotonin content of the trachea were reported for negative air ion exposure, with SMDs of 2.8 after 14-h exposures and 4.4 after continuous exposures of unspecified duration [71]. In later studies, the Krueger laboratory measured serotonin levels in the blood of mice exposed in groups of 2–12 [72–74], and in the brains of mice exposed in groups of 10–12 [75]. One of these experiments reported modest to strong evidence for an increase in the serotonin level in the blood of mice exposed to positive air ions generated from air supplemented with carbon dioxide gas [72]. No reliable effects of exposure to positively or negatively charged air ions generated from air supplemented with carbon dioxide, nitrogen, or oxygen gases on blood serotonin were reported in replication experiments conducted in the Krueger laboratory [72, 74].

A much larger number of tests for effects of air ions on serotonin levels in the brain were reported by Krueger and Kotaka [75]. After 12 h of exposure, there was modest evidence for reductions in brain serotonin levels of mice exposed to positive or negative air ions (one of three tests at each polarity). In other experiments reported in this paper, no reduction was observed in zero of six tests at 24 h; one of six tests (negative air ions) at 48 h; and one of six tests (negative air ions) after 72 h. The mice in this experiment were exposed in groups of 10.

Where differences were noted in the experiments reported by Krueger and his colleagues, the results reported in these studies were by no means as statistically significant or as consistent as they described. One reason that results appear statistically significant is that Krueger and his colleagues mistakenly treated the animals exposed as a group as individuals for the statistical analysis. Consider that the individuals in groups tested by Krueger and colleagues shared many similar characteristics and aspects of the testing environment other than air ions, which if they affected one animal of the group were likely to have affected the other animals as well. Hence, this would have led to a degree of interdependence in the responses of the animals within the group that would not have existed had the animals been tested as individuals. Without the inflated number of subjects per group and a lowered variation in the responses because of the similarity of the animals’ experience, the few inconsistent differences reported would not be expected to be reliably different. A similar criticism applies to Diamond et al. [76] who studied smaller groups (n = 4–12); they reported that groups of rats exposed to negative air ions living in an enriched environment, but not an impoverished environment, had lower serotonin and cyclic adenosine monophosphate (AMP) levels in one part of the brain cortex.

Other investigators attempted without much success to replicate or advance the claims of the Krueger laboratory about opposite effects of positive and negative air ions on serotonin levels despite the inconsistencies in the data reported by the Krueger laboratory. Gilbert [77] reported weak evidence that intermittent or continuous exposure to negative air ions reduced brain serotonin levels. At a Rockefeller University laboratory, Bailey and Charry [78] reported no effects of either positive or negative air ion exposure on the concentrations of serotonin in any of six brain regions examined or on a measure of neurotransmitter turnover (the ratio of serotonin to its metabolite, 5-hydroxyindole acetic acid) in these regions after 2, 18, or 66 h of exposure. Dowdall and De Montigny [79] did not find that air ions affected the response of hippocampal neurons to applied norepinephrine, serotonin, or acetylcholine. Kellogg et al. [80, 81], in Krueger’s laboratory, reported no effect of positive or negative ions on blood serotonin levels in rats after group exposures of 25 rats per exposure group either after 140 days of exposure or at the end of life (260 days). Beardwood et al. [58] reported that groups of four to seven rats exposed to negative air ions showed modest evidence for a reduction of serotonin levels in lung tissue following negative air ion exposure but no effect of exposure to positive air ions. Neither positive nor negative air ions affected serotonin levels in brains of rats in this study.

Charry and Bailey [82] also tested to see if exposure to negative or positive air ions at high levels (500,000 ions/cm^3^) affected the levels of two other neurotransmitters—dopamine and norepinephrine—in five different brain regions of rats after 2, 18, or 66 h. No effects of exposure were reported. The baseline levels of these neurotransmitters did not change following exposure and the rate of turnover of these transmitters also did not change following the administration of alpha-methyl-p-tyrosine methyl ester to block catecholamine neurotransmitter synthesis.

Altogether, the 280 SMDs describing possible effects of air ions on serotonin and other neurotransmitters in these studies, summarized in Additional file 2: Figure S3, provided few differences with modest support or strong support. Thus, the research provides no reliable or consistent evidence to suggest that air ion exposure affects neurotransmitter functions. The scattered SMDs that indicated some modest or strong evidence for effects are compatible with a no effect hypothesis because the differences appear largely attributable to chance given such a large number of comparisons. Also, exposure to higher levels of air ions did not produce greater responses (Fig. 2c). Another concern regarding most of these studies, except those from the Rockefeller University laboratory [78, 82], is that the investigators did not control for the time of day when the samples were collected to preclude potential variations in the endpoints measured within and between experimental groups with respect to circadian cycle. The time of day at which samples are collected is known to affect the concentrations of serotonin and catecholamine neurotransmitters [83–85].

### Tracheal function

Another principal interest of the Krueger laboratory was the effect of air ions on mucus flow and the movement of cilia that line the trachea in anesthetized rabbits, rats, guinea pigs, and mice, and their potential relationship to levels of the neurotransmitter, serotonin. Although the Krueger laboratory reported that positive and negative air ions have opposite effects of small magnitude on mucous flow and ciliary rate in anesthetized rats, mice, rabbits, and guinea pigs, a quantitative assessment of these claims could not be performed due to the lack of sufficient information about the experimental design and results, including the absence of statistical analyses [71, 86–89]. Krueger also claimed that air ions had similar effects on isolated tracheal tissues exposed in vitro [90], but others have not been able to replicate this claim [91–93].

Even though the seven studies on this topic did not report sufficient information to compute SMDs, the means of control, experimental measures, and other data reported in the whole animal studies by the Krueger laboratory and in subsequent studies of whole animals by other investigators are summarized in Additional file 1: Table S10. It should be noted that the relevance of studies of anesthetized rats is unknown because the normal exposure pathway that would trap and neutralize many air ions entering the nose and mouth was bypassed in these studies by applying air ions directly to the trachea. Nevertheless, despite differences in the species, exposure duration, and intensity, and the limitations noted above, the Krueger publications suggest that ciliary activity and mucous flow are lower when positive air ions were applied in 15 of 20 tests, whereas these measures are increased in 19 of 22 tests by the application of negative air ions. In contrast, another researcher [94] reported that ciliary activity was highly variable and sensitive to small changes in temperature and humidity and he was unable to replicate Krueger’s studies. In the Andersen study [94], neither negative nor positive air ion exposure caused alterations in the ciliary beat frequency or mucous flow compared to controls. In response to the criticism levied by Anderson [94], Krueger admitted that *“[i]n retrospect, the experiments performed during our novitiate* [*sic*] *in air ion studies are open to criticism on several counts”* [95], which included primitive exposure set up, lack of control over temperature, humidity, and pollutants, and the absence of statistical analyses. Additional support for the criticisms levied by Anderson based on the methodological strengths of his study is provided in his doctoral thesis [96].

A more recent study by Sirota et al. [97] reported that daily exposure of rats to concentrations of negative air ions at 100,000–600,000 ions/cm^3^ produced by a Lustre ionizer caused histological damage to the trachea and biochemical changes suggestive of oxidative stress. These authors reported, however, that similar levels of negative air ions produced by two other ionizers did not damage the trachea. The inconsistency of effects for similar air ion exposures suggests that other unmeasured factors, possibly ozone, influenced the outcomes of the Sirota et al. [97] study. These investigators later reported that exposures of rats at similar levels to those used in their earlier study did not produce histological damage, but did alter indicators of reactive oxygen species, responses also compatible with a lesser degree of exposure to ozone [98]. Because most studies of tracheal function did not provide measurements of air ion concentrations, no dose–response assessment was performed.

### Respiratory infection

Increased mortality was claimed in mice infected with a fungus, a bacterium, or influenza and exposed to negative and positive air ions in five studies [99–103]. Since the results are presented as the number of mice that died at varying times after infection, the results summarized in Additional file 2: Figure S4 are presented as the PDs in mortality between groups exposed to air ions and those in control groups for 21 air ion tests from the 5 studies published by this laboratory. The studies of animals infected with *Coccidioides* or *Klebsiella* suggested no or weak evidence for an effect of positive air ions [99, 100]. Apparently more robust but inconsistent effects of exposure to positive air ions on the mortality of animals infected with influenza were reported [100]. Here again, as in the Krueger studies of serotonin, 10–12 animals were exposed simultaneously in a group, yet the statistical analysis treated each animal as if it had been an independently-tested subject and grouped together results from multiple experiments to achieve total group numbers between 40 and 237 in the investigators’ analysis, which inflated the apparent statistical differences between groups.

In other studies, increased mortality from influenza was reported for mice exposed to positive air ions, but negative air ion exposure was reported as having no effect on mortality [96–98, 101, 102], or reduced mortality in a few experiments or when mixed at lower concentrations with positive air ions [102]. A role for positive air ions in increasing mortality after infection to influenza virus is hard to support from these data because groups of animals exposed to ion-depleted air provided modest evidence for increased mortality relative to sham controls that was greater or no different from that reported for positive ions, negative ions, or a mixture of positive and negative ions [102]. In this latter study, the authors stated, without presenting any data, that exposure to any of the ion-treatment conditions for a short time (duration not specified) prior to administration of the influenza virus had no effect on mortality. In a final study, Krueger et al. [103] reported no effect of exposure to positive air ions, negative air ions, or ion-depleted air on mortality from the influenza virus for exposures of similar duration and similar air ion concentrations as in their  previous studies. The authors argued that this complete failure to replicate their previous research was because the influenza virus was administered by disbursing the virus as an aerosol rather than by direct application to the nose as in all previous studies. Still, no explanation was offered as to why this difference in the delivery of the virus to the subjects was important. An explanation not offered by the authors is that high concentrations of air ions caused the aerosolized virus to be removed from the air, thus reducing exposure of the animals to the virus and mortality. It is well known that high concentrations of air ions can reduce levels of viruses, aerosols, and particles in confined spaces [104, 105]. Overall, exposure to higher levels of air ions did not produce greater mortality from respiratory infections than lower density exposures (Fig. 2d).

### Cardiovascular function

Four studies reported on heart rate, respiration, and blood pressure measured in rats exposed to positive or negative air ions. The 25 SMDs for these tests are summarized in Additional file 2: Figure S5. Three studies of animals exposed to positive and negative air ions for periods of 30 min to 8 weeks reported no modest or consistent effects of exposure on heart rate, respiration rate, or blood pressure [106–108]. The fourth study, Suzuki et al. [109], measured the heart rate and blood pressure responses of anesthetized rats to negative air ions and reported no reliable effects. Suzuki et al. [109], however, also measured levels of *c*-*fos* protein, a marker of neuronal activity, in brain regions that receive input from peripheral sensory autonomic nerves. They reported that the levels of *c*-*fos* protein were reduced in two brain regions and increased in another region in intact rats, but these changes were abolished when the vagus nerve was cut. Only a modest reduction in the *c*-*fos* activity was apparent in one brain region. If a real effect, this could be explained if air ions presented to the nose of the rat stimulated peripheral sensory receptors. This could be a more important factor in experiments like this where the concentrations of air ions presented to the rat’s nose through a tube from the ion generator, as described by Suzuki et al. [109], would be far higher than the concentration measured in the open air. Whether physiological responses of anesthetized rats to air ions in this study might predict those of unanesthetized rats or to what degree the observed responses may be due to ozone, not air ions, is unknown. Dose-related changes in cardiovascular measures were not observed (Fig. 2e). In another publication [110], the researchers give a preliminary descriptive report on peak changes in the electrocardiograms of the rats from two of the studies [106, 107] described above. Few, if any, clear changes, however, were noted and the data were not quantitatively evaluated.

### Reproduction and growth

The data presented in seven studies permitted calculation of SMDs for studies of the reproduction and growth of rats and mice exposed to negative and positive air ions. The results of these studies included measures of blood constituents and growth with short- and long-term exposure, as summarized in Additional file 2: Figure S6. Hinsull et al. [111] examined the growth rates of four generations of rats exposed to negative air ions. They reported a small slowing of growth among first generation male rats after 120 days of exposure to negative air ions, a slightly higher growth rate of exposed males in the second generation, and no effect on the growth of males in the third generation. No effects of negative air ions on the growth rate of female rats were reported. The authors also concluded that *“[e]xposure to negative ions during the post*-*weaning period [0*-*260* *days] had no significant effect on the growth of animals throughout the four generations studied”* (Hinsull et al. [111], p. 167). A replication experiment by the same authors failed to find any effect of negative air ions on body weights of male or female rats after 95 weeks of exposure [112]. The authors also described greater longevity of groups exposed to negative air ions than controls, however, and an accompanying increase in mammary tumors, which were not statistically evaluated. Fisher’s exact Chi square tests performed as part of this review indicated that the increased longevity of the female rats exposed to negative air ions over control females was strong at 100 weeks (*p *< 0.01), but the difference in reported tumors provided only modest evidence for an elevation of tumors in males (*p* < 0.05). Since the incidence of mammary tumors is age related, the observation of more tumors in the exposed group may be explained by the longer period of life in which tumors could develop.

In a study of positive air ions, body weight was reported to decrease after 50 weeks of exposure in parental males but increase in first generation males [113]. The results, however, were confounded by respiratory disease in the colony. Kellogg et al. [80, 81] reported on mice exposed to positive or negative air ions at two intensities (or static fields of positive or negative polarity) for 2 years. A third publication summarized the results of these two previous studies [114]. No effects on body weight or on multiple measures of blood constituents were observed, except for modest evidence of lower levels of blood glucose and cholesterol and higher levels of blood urea nitrogen in the ion-exposed groups in year one [80] but not in year two [81, 114] of exposure. The survival of mice in this study was greatest for groups exposed to static electric fields and lowest for those exposed to negative air ions. In the negative air ion group, the survival was similar for mice exposed to high or low levels of air ions. Overall, the survival of mice exposed to negative air ions was about 7% lower than controls. For groups exposed to positive air ions, those exposed to higher levels of positive air ions survived 18% longer than those exposed to lower levels. A robust effect of air ions on longevity was not supported. Furthermore, the authors reported many statistical comparisons in the analysis for which no adjustment for multiple comparisons was made. More important, however, is that the findings were confounded by a mild vitamin deficiency and severe intestinal infections in the mouse colony. The latter prevents any clear interpretation of the survival data. As described by the authors:

*By 6/7/83 we had diagnosed this disease [intestinal infection] as resulting from proteus vulgaris. Animals dying from this infection had the salient features of severe gastroenteritis, splenic hypertrophy, and occasional purulent salpingitis. Sections showed the small intestines filled with pus, with the mucosal surface having various degrees of liquefaction necrosis, and marked infiltration of the muscularis mucosa by reactive cells, notably polymorphonuclear leucocytes. Occasionally animals showed marked salpingitis with grossly enlarged fallopian tubes filled with purulent materials. Final autopsies at the end of the experiment revealed animals with some degree of proteus infection from all cage conditions. ****Obviously, the prevalence of proteus infections markedly complicates the interpretation of the cause of death for affected experimental animals*** (Kellogg et al. [81], p. 271, emphasis added).

Data from two interim reports from Kellogg et al. [80, 81] on growth and development were not extracted. For measures of growth, the data extracted from the final published report [114] were analyzed as described above.

Finally, two more recent studies from Japan studied perinatal development. Yamamoto et al. reported that the maternal body weight, food consumption, uterine weight, as well as the  bone development of male and female offspring of rats exposed to high levels of air ions (8500,000 ions/cm^3^) at both polarities for the first 20 days of pregnancy were no different from those of sham-exposed controls [115]. The second study from the same laboratory performed a similar experiment but studied the second-generation offspring as well [116]. However, a concern with both these studies is that the exposure to rats was likely far lower than the study suggests because the rats were exposed simultaneously to positive and negative air ions at equal intensities so rapid neutralization of the ions generated could be expected. Among the eight studies for which SMDs were calculated, exposure to higher levels of air ions did not produce greater responses (Fig. 2f). Because these two studies from Japan exposed animals to both positive and negative ions simultaneously [115, 116] these were not plotted in Fig. 2f with the rest of the studies that reported primary exposures to just one polarity of ion.

The data from three other studies could not be extracted to calculate SMDs. In an early investigation, Herrington and Smith [47] reported no effects of negative air ions at 120,000 ions/cm^3^ on rat development in a study lasting almost 1 year. Hinsull [117] reported no effects of ionization on pregnancy or pathology of embryos examined 14–18 days after delivery. Three litters of rats exposed to negative air ions exhibited a greater mortality from respiratory disease prevalent in the rat colony with neonatal exposure of dams and pups to negative air ions, but not positive air ions. The body weights and growth rates of healthy litters exposed to negative air ions post-weaning were not affected. The data were inappropriately analyzed because the experimental unit considered was the individual embryo or pup and not the litter, and the litter sizes were not reported. Another study by Hinsull et al. [118] reported that exposure of rats to negative air ions over three generations for up to 20 weeks did not affect the reproduction or growth of second or third generations but did reduce the thymus weights of second (but not third) generation rats. The effect of exposure on the second generation was not explained by increased adrenal steroid output (no difference between exposed and control groups in either second or third generations).

### Carcinogenesis

Two studies examined the response of rats and mice exposed to negative air ions on 30 measures related to carcinogenesis, as summarized in Additional file 2: Figure S7. The first study reported that air ions generated by water shearing (i.e., water-generated negative air ions [WNI]) increased the activity of natural killer cells, which protect against cancer, after 12- to 48-h of exposure (*p* < 0.001) [119]. The study further examined the development of tumors in mice injected with a cancer-causing chemical and then exposed to WNI or WNI and the anti-tumor drug, TS-1. At 5 weeks, the evidence provided strong support for a reduction in the tumor volumes and tumor weights in the mice treated with WNI only and just tumor volume when treated with WNI + TS-1 compared to the control group. During the next 67 weeks the survival of mice was observed, and tumor weight and body weight measured at death. Survival was significantly longer and tumor weight was lower; these factors were associated with strong support for increases in the body weight of mice treated with WNI or with WNI + TS-1. Important experimental details including the concentration of air ions, animal exposure conditions, and statistical analyses were not reported; however, the evidence for beneficial effects of treatment was strong.

**Fig. 3:**
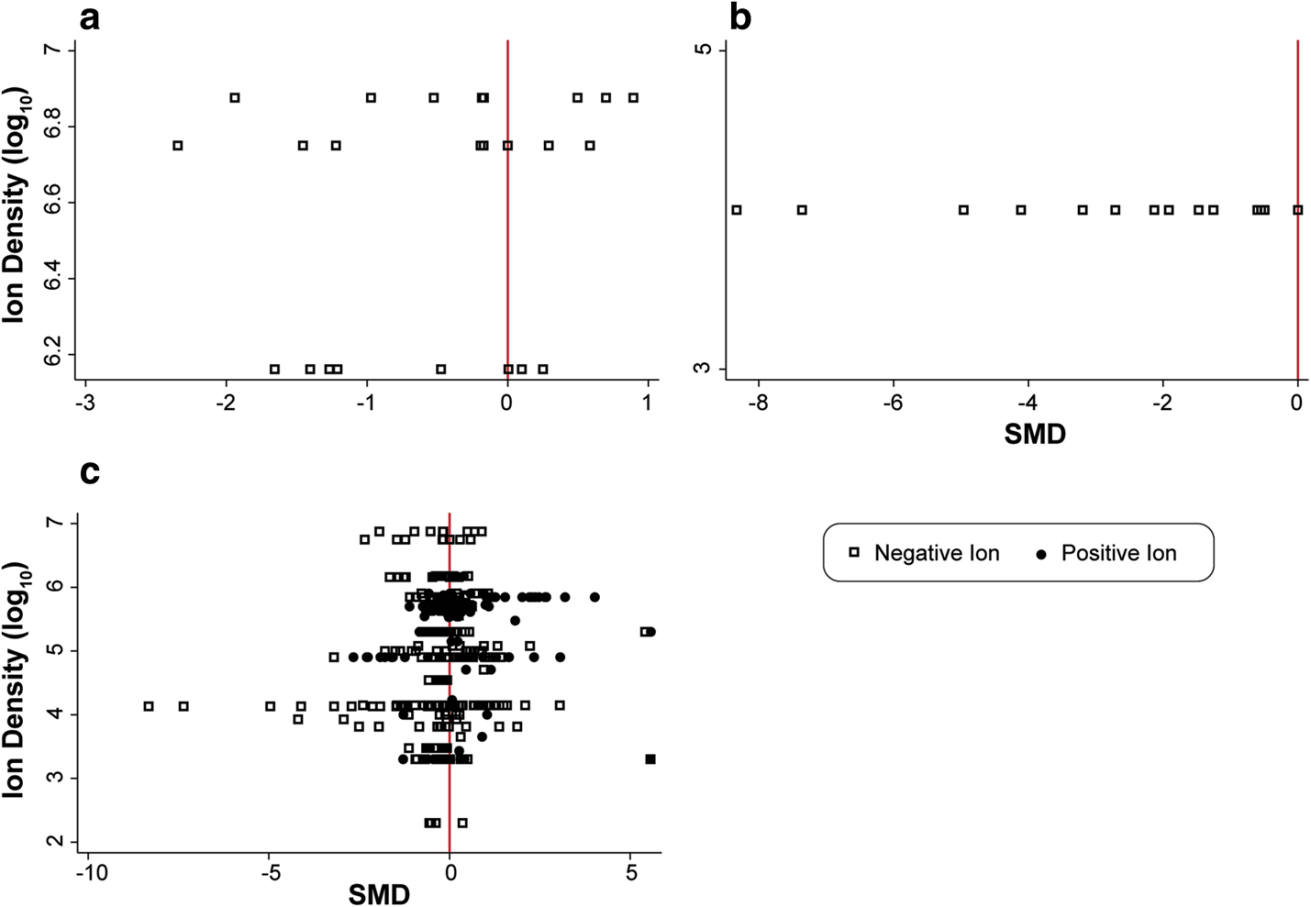
Dose–response plots for negative ion and positive ion exposures: **a** carcinogenesis; **b** other health endpoints; **c** all studies

In the second study, Takasawa et al. [120], investigated whether air ions at ion densities of 1.4, 5.6, and 7.5 million ions/cm^3^ can damage the DNA of cells in the lung or blood obtained from rats and mice. A concern for this study, like that of two recent studies from Japan discussed above [115, 116], is that the rats were simultaneously exposed to negative and positive air ions at similar levels and this suggests that the resulting concentrations of air ions could be expected to be much lower than reported because of the rapid neutralization of charge by ions of opposite polarity. Damage to DNA is relevant to cancer because damaged DNA, if not repaired by cellular repair mechanisms, can lead to the development of cells with aberrant growth. In this study DNA damage was assessed by the comet assay. However, there are recognized problems regarding the variability in the results of this assay for replicate samples analyzed within and between laboratories, and the interpretation of comet assays that impede its acceptance at this time as a reliable tool [121, 122]. Animals were exposed for 48 h and blood and tissues collected for analysis. In mice, neither of two indices of DNA damage as detected by the comet assay (length of DNA in the comet tail and percent of DNA in the comet tail) indicated that air ions were capable of damaging DNA of blood cells or lung cells at any of the air ion concentrations tested. In rats, the results were less clear: the percent of DNA in the comet tail of blood samples did not differ with air ion concentration, but the comet tail lengths in these samples were marginally lower at all three concentrations of negative air ions. In the lung, most all measures of comet tail length and percent of DNA were similar. For this study, none of the SMDs indicated even a modest effect of air ion exposure on indicators of DNA damage (*p* < 0.01). Since the previous study did not report air ion densities and the latter study did not test for effects at multiple levels of exposure, no assessment of dose–response relationships could be performed (Fig. 3a).

### Other health endpoints

Four studies reported on a variety of biological measures and health endpoints. Data from 39 tests extracted from three of these studies are summarized in Additional file 2: Figure S8. Wehner et al. [123] exposed rats in a single group of 10 animals to negative air ion aerosols for 90–140 min and then analyzed for 21 blood components typically included in general blood work-ups for humans and for pH and Ca^++^ concentration in cerebrospinal fluid. One of these measures, mean corpuscular hemoglobin concentration, differed modestly from the control group at *p* < 0.01 and the authors considered this finding as likely due to chance.

The remaining three studies assessed other potential therapeutic applications of air ions. Bordas and Deleanu [124] tested the hypothesis that negative air ions might reduce the incidence of ulcers of the stomach that developed in rats in which the part of the intestine immediately below the stomach was constricted by a surgical ligature for 24 h. Rats in one group were exposed to negative air ions together for 3–120 min for 10 days prior to the induction of ulcers and then examined 5 days after intestinal constriction. Another group was exposed together in the same manner as the previous group, but air ion treatment continued after ulcer induction for another 5 days, for a total of 15 days, until examination for ulcers. The mean number of ulcers in these treated groups was compared to control groups examined 24 h after constriction of the intestine or 5 days after constriction of the intestine. Although not discussed in the paper, it appears that the animals were exposed in groups, which reduced the variability and inflated the number of animals considered in the statistical analysis. In the group treated with negative air ions for 10 days, no effect was reported, but after 15 days of exposure the data provided modest evidence for a reduction in the total number of ulcers (*p* < 0.01).

A subsequent publication by this laboratory reported these same data on gastric ulcers from the earlier study [124] but provided additional analyses of the anterior pituitary and adrenal glands [125]. The study reported modest evidence that prophylactic treatment with negative air ions prior to induction of gastric ulcers reduced the weight of the anterior pituitary and the thickness of the adrenal fascicular cortex. Treatment with negative air ions both before and after induction of ulcers reduced the weight of the adrenal glands and produced extraordinarily large reductions in the weight of the anterior pituitary and the thickness of the fascicular cortex (*p* < 0.01 with SMDs of 7.4 and 8.3, respectively).

A fourth study, whose results were not extracted, reported that exposure of wounds on the back of rats to negative air ions accelerated healing at 10 days post-surgery (*p* < 0.001) with a recovery process similar to controls for the next 10 days whereas exposure to positive air ions slowed healing at 15 and 20 days post-surgery (*p* < 0.001) [126]. It is not clear if the effects reported in the latter two studies can be extrapolated to predict benefits to health in these studies. Air ion concentrations were only reported in two of the four studies of other endpoints and the air ion concentrations reported were the same, so no dose–response relationship could be evaluated (Fig. 3b).

### Dose response

The dose–response trends of SMDs and PDs across multiple studies in each topic area were discussed previously. The relationship of the magnitude of the change in the exposed group to the control group as a function of air ion density concentration was summarized within groups of studies in eight topic areas in Figs. 2, 3. There was no apparent dose–response relationship in any of the figures. In addition, a plot of all biological and behavioral response data extracted from studies in relation to air ion concentration, except for those applying bipolar exposures, is shown in Fig. 3c. Ordinarily, this would not be a very useful presentation of data, but the range of ion densities reported in the animal studies is very large, spanning five orders of magnitude (i.e., the highest exposures are 100,000 times or more above the lowest exposures). This large range of exposure intensities justifies looking for global evidence of a dose–response relationship across all studies. The SMDs are plotted as a function of the log_10_ of the ion density. For example, in Fig. 3c the ion densities range from 200 ions/cm^3^ to 8,500,000 ions/cm^3^ (the highest air ion density reported in the literature was 70,000,000 ions/cm^3^ in a study from which data could not be extracted [69]). The dose–response data from all extracted studies does not provide visual evidence for any apparent trend for the SMDs to differ as a function of positive or negative air ion density levels.

The dose–response analysis across studies was undertaken because only a small number of studies tested for effects of air ions at multiple exposure levels. In those 12 studies that did test at more than 1 exposure level [59, 69, 72, 73, 75, 80, 81, 97, 102, 103, 114, 120], none reported exposures to multiple levels of air ion densities or reported a dose-related increase or decrease in measured responses or had any effect at different levels, except Krueger et al. [72] and Kellogg and Yost [114]. Krueger et al. [72] reported that an increase from 4500 to 51,000 ions/cm^3^ comprised of positively-charged carbon dioxide (CO_2_) air ions increased blood serotonin levels modestly, whereas a similar increase in negatively-charged CO_2_ air ions also increased blood serotonin levels, but the difference was not reliable. Kellogg and Yost [114] reported that an increase in positive air ions from 2000 to 200,000 ions/cm^3^ decreased mortality in mice.

Among other studies that varied the duration of exposure to air ions [47, 51, 52, 57, 65, 71, 74, 75, 78, 82, 89, 97, 101, 110, 114, 117], responses measured by the investigators did not increase or decrease with longer exposure durations.

### Quality of studies and protection against biases

The ratings of quality of the design and reporting of air ion studies were highly variable. Table 1 below provides an overall summary of factors by topic area that pose a risk of systematic bias or threats to quality. See Additional file 3: Table S1 for details of the assessment of individual studies.

**Table 1:** Summary of quality ratings of studies reviewed

Topic	Number of studies^a^	Number of studies in compliance with quality indicators^b^				
		Shamcontrol	Confounding^bc^	Blinding	Randomized	Individual exposure
Behavior	13	12	6	4	4	6
Learning and memory	9	4	2	1	5	3
Serotonin and other neurotransmitters	13	6	2	4	6	4
Tracheal function	7	6	2	0	0	3
Respiratory infection	5	4	1	0	2	0
Cardiovascular measures	5	4	0	0	0	5
Reproduction and growth	11	8	3	0	3	1
Carcinogenesis	2	1	0	0	1	1
Other endpoints	4	2	1	0	1	1
Totals (%)	69	47 (68%)	22(35%) (32%)	9 (13%)	22 (32%)	24 (35%)

^a^Some of the 62 studies reported results in more than 1 topic area

^b^Some studies that did not generate air ions by corona discharge were not susceptible to major confounding factors

^c^Minimal compliance was indicated if any confounding factor, electric field, ozone, noise, or light was addressed

Forty-seven studies (68%) reported that sham-exposed groups were included in the study design. The absence of sham controls violates the essence of the experimental method, yet it was often difficult to ascertain whether the control group was in fact sham exposed.

Good quality studies attempt to minimize or control potential confounders associated with the generation of air ions. Depending upon the response under study, the production of an electric field, ozone, noise, or light from corona discharge systems may confound any potential effect that might be otherwise attributed to air ions. Confounders were of greater concern in studies where air ions were generated by corona discharge than by other means. The rating summary for confounders in Table 1 lists the number of studies in the group that addressed at least one of these confounders (minimal compliance). As evident from summaries in Additional file 1 and in Additional file 3: Table S1, most studies did not attempt to minimize such potential confounders and none of the studies that generated air ions by corona discharge from which data could be extracted, and addressed potential confounding by electric field, ozone, and noise, reported any effects of air ions. Only 32% of studies in Table 1 addressed confounding factors. The breakdown of the studies that addressed individual confounding factors, Additional file 3: Table S1, was 23% for the electric field. Among just the studies that generated air ions by corona discharge even fewer addressed confounding by ozone (23%), corona noise (29%), and corona light (5%). Corona discharge sources produce air ions by concentrating the electric field at the points of metal needles, so an electric field exposure will occur as well. In addition, the presence of air ions themselves (from any source) creates a static electric field. Hence, one would expect that this potential confounder would have been considered in the design of air ion studies. Yet only 23% of the studies that generated air ions by corona discharge addressed this potential confounder.

Blinding of the investigators to the treatment condition and exposure history of the animals during data collection and analysis also is important to minimize potential bias. Of the studies reviewed, 13% stated that blinding procedures were followed, which compares to 17% that was reported in the Macleod et al. survey of over 1000 publications of in vivo research from the top biomedical research institutions in the United Kingdom from 2009 to 2010 [42].

Failure to randomize subjects to study groups also is a potential source of bias. Only 32% of the studies randomly allocated subjects to control or treatment groups. While low, this is higher than the rate of randomization (14%) in the Macleod et al. survey of in vivo studies [42].

Air ion studies in which the exposure was not administered to individual animals, as is done in most studies of chemical exposures, poses problems for statistical analysis because the observations on individual animals are not independent and the presence of multiple animals can affect exposure to air ions. Only 35% of the studies exposed animals individually; 65% the studies exposed the animals in groups. It should be noted in studies of post-natal development that continued exposure of the dam and pups until weaning is necessarily a group exposure.

As summarized in the figures in Additional file 2, relatively few studies in any topic area provided moderate statistical evidence (*p *< 0.01) or strong statistical evidence (*p* < 0.001) that the difference between the exposed and control groups would be as large as observed if only chance were responsible. Further, there was no consistency for the direction of effects reported across studies within topic groups. A generic weakness of the studies in the animal air ion research literature is that the number of subjects in the control and exposed groups is often ≤ 12, which means that the power of a study to detect a difference greater than expected by chance alone is likely to be less than 80%. In multiple studies, particularly those from the Krueger laboratory, the number of independent experimental units was far less than the number of subjects reported because the animal subjects were exposed in groups ranging in size from 3 to 12, and so even if the total number of subjects in a group is reported to be large, the effective number of experimental units will be far smaller and the appropriately calculated *p* values will be greater than stated by the investigators [30]. Within the nine research topic areas, the incidence of group exposure to air ions varied from 0 out of 5 of the cardiovascular studies to 5 out of 5 of the respiratory infection studies. In other areas, 50–75% of the studies exposed the animals in groups.

Overall, there was a clear relationship between the rated quality of the study/potential for systematic bias and the statistical strength of the evidence. Additional file 3: Figures S1 and S2 show that the studies with a quality rating < 50% of possible contributed almost all of the results with calculated *p* < 0.01 and p < 0.001 values. Thus, the overall quality of the studies reviewed was not high and almost the entirety of SMDs or PDs were contributed by studies with low quality ratings. Prominently represented among the studies reporting SMDs or PDs at these *p* values were the studies published by Krueger and his collaborators.

## Discussion

Air ions are charged molecules of air that can be generated by many natural and man-made phenomena. These ions may be attracted to the surfaces of the skin and respiratory tract due to electrostatic forces. In the respiratory tract, these ions are generally retained in the upper respiratory passages (i.e., the nasal passages and upper bronchi) and rarely reach the alveoli of the lung; thus, they are unlikely to be absorbed systemically to any significant degree [26]. More importantly, the concentration of air ions considered as a fraction of the air molecules in a single cm^3^ of air is vanishingly small. At the highest concentration reported in any study reviewed, 1 air ion is diluted within 10^12^ other air molecules. Because of the low concentration and low probability for systemic absorption, a biologically plausible mechanism by which air ions—either positive or negative—could mediate physiological changes or cause either beneficial or adverse effects on health is neither obvious nor claimed to be known. A possible exception may be that at high air ion densities the charge on body hair from air ions and any accompanying static electric field may be perceived by mechanosensory stimulation. Nevertheless, numerous studies reported in the literature have made claims of biologically significant effects. The purpose of this analysis is to comprehensively review the results of animal laboratory studies of air ion exposure. This review updates the assessment presented in the 1997 Oak Ridge National Laboratory report on exposures related to direct-current transmission lines [20]. The findings of this systematic review of animal studies do not support claims that air ions have any biologically significant effects. Recent systematic reviews of the human experimental data on air ion exposure specific to effects on respiratory function and mood [6, 14] have reached a similar conclusion.

Experimental animal studies can be important for addressing the potential health risks of exposures to humans. These types of studies allow for the exposure of a relatively homogenous population to specific levels of a chemical or physical agent in a laboratory under controlled conditions. Often, the animals are exposed to much higher doses or concentrations of an agent than that to which humans typically may be exposed. Further, the duration of the exposures expressed as a percentage of an animal’s lifetime may be longer than exposure that humans typically experience under normal, environmental conditions and in clinical studies. In theory, the greater exposure (both in terms of the concentration of the agent and the duration of the exposure) increases the likelihood of observing a response to that exposure. Because substantial similarities exist between humans and other mammals in terms of how their physiological systems function, responses observed in laboratory animal studies are generally considered potential indicators of possible responses in humans. For this reason, experimental animal studies play an important role in human health risk assessment.

Nevertheless, many issues must be taken into consideration when interpreting the results of experimental animal studies. One issue in the interpretation of these study results revolves around how control groups are handled in the experiment. While many of the studies reviewed employed control animals that were not exposed to air ions, often these animals were not handled in a similar manner as the animals exposed to air ions and were not sham controls. For example, the control animals may have been kept in a room separate from the exposed animals; thus, they may have been exposed to different environmental variables (e.g., temperature, humidity, light, sound, etc.) than the animals exposed to air ions. The controls also may have been handled less than the exposed animals if exposures took place in a separate apparatus from the home cage. For these reasons, it is ideal for control animals to be sham exposed; that is, handled and housed in the same manner as the treated animals, including placement in a similar exposure apparatus that produces similar levels of light and noise, but not air ions. In the studies reviewed, only 68% reported sham exposure of the control animals.

In studies involving air ion exposure, one of the most important issues that must be taken into consideration is how the air ions are generated and whether appropriate experimental controls have been implemented to address associated confounding variables. For example, a large majority of studies reviewed in this analysis used a corona discharge system for the generation of air ions. This system uses a strong electric field to cause the ionization of air molecules around an electrode. Few of these studies, however, incorporated a control group exposed to an electric field only in the absence of air ions to address whether the observed responses are due to the presence of the electric field rather than the generated air ions. Because of the associated electric field, an animal that is exposed to air ions at high levels may accumulate a surface charge on the fur to such a level that charges in the air of the same polarity will be repelled and the exposure to air ions will be limited. For this reason, it is also important that the exposures occur in grounded cages to minimize the likelihood of this potential exposure limitation. Of the studies reviewed that generated air ions by corona discharge, only seven [48, 57, 59, 64, 78, 82, 109] included the proper electric-field-only controls. Several studies from the Krueger laboratory [80, 81, 99, 103, 114] and another investigator [94] also implemented electric-field-only controls, but these studies used a tritium ion generator rather than a corona discharge system for the generation of air ions; the issues associated with this type of ion generation are discussed further below. None of these studies reported differences between ion-exposed animals and controls exposed to just static electric fields. It is indeed surprising that greater attention was not given by investigators to the confounding effects of electric fields from corona ion generators since a static electric field is known to be a mechanosensory stimulus to hair on the body surface. Recent systematic reviews of static-field research on vertebrates and invertebrates have confirmed that superficial sensory stimulation by static electric field is the mechanism by which static electric fields produce behavioral and physiological effects in humans, animals, and plants [127, 128].

Poorly designed corona discharge ion generators can produce ozone sufficient to affect biological processes, particularly if the air ion generator is operated in a space without adequate ventilation. Even if the system does not produce appreciable levels of ozone in the absence of a test animal, the introduction of an animal into the exposure apparatus can substantially increase ozone generation—likely due to the corona discharge produced at the tips of the animal’s whiskers or due to actions on grounded water [129]. Despite these potential confounding variables, few animal studies of air ion exposure have attempted to measure these gaseous pollutants or have optimized their systems to minimize such exposures. Finally, corona air ion generators produce small amounts of high frequency noise and light, which often are not considered or controlled for in the experimental study design. Small amounts of light can be an issue in studies that look to address the potential effects of air ion exposure on sleep patterns and circadian rhythms. Of the studies reviewed, only 13 [48–50, 55, 57, 59, 64, 78, 82, 97, 125] controlled for the production of ozone and other gaseous by-products. Bailey and Charry [57, 78, 82] used a specially-designed exposure system evaluated by the US National Bureau of Standards to prevent such confounders [130]. For a few other ion generation systems there would appear to be little need for control measures on gaseous by-products [108, 109, 123]. Overall, the studies that addressed the greatest number of confounding variables related to the use of a corona discharge system for the generation of air ions were Olivereau and Lambert [48], Bailey and Charry [57, 78, 82], and Creim et al. [59, 64]. None reported any effect of air ions on the responses measured.

In many older experimental studies, air ions were produced by radioactive materials including tritium [71, 72, 74, 75, 77, 80, 81, 86–89, 93, 102, 103], polonium [62, 65, 93], and Krypton-85 [66, 72, 106, 107, 109]. While these sources have advantages because their operation is not accompanied by noise and light, and levels of gaseous pollutants should be minimal, concern about exposure to ionizing radiation from these sources, which could have long-term effects of their own and ionizing radiation safety issues, has precluded their use in more recent experimental work.

To address whether an observed response may be due to air ion exposure or some other factor, it is useful to expose different groups of animals to multiple concentrations of air ions. This allows the researchers to investigate the dose–response relationship for an observed effect. If the observed response increases as the exposure concentration of air ions increases or the duration of exposure increases, then it is more probable that the response is causally related to the exposure. If the observed response, however, does not exhibit the typical dose–response relationship (i.e., it does not increase with increasing concentrations of air ions or increasing exposure duration), then it is less likely that the observed response is causally related to air ion exposure. Unfortunately, multiple exposure concentrations were not employed, and dose–response relationships were not investigated in the majority of studies reviewed.

Investigator bias also can influence the outcome of a study. If the investigator has a preconceived notion regarding the outcome of the study or knows the exposure history of the subjects when analyzing the results, he or she may inadvertently bias the results, particularly if the parameters assessed are subjective or observed by the investigator instead of assessed by an automated system. For this reason, it is important for the investigator to be blinded to the status of the animals being studied (controls or exposed) until after the data are collected and analyzed. Studies that reported the implementation of blinded analyses include some of those from Olivereau and Lambert [49, 50, 55], those of Bailey and Charry [57, 78, 82], and several others [66, 76, 79].

This review demonstrates the importance of evaluating study quality and potential for bias since the strength of the evidence extracted from the literature was contributed by studies with quality ratings 50% or less than the highest possible score.

Finally, a key aspect in establishing the validity of a scientific observation is replication. Replication is the process of repeating a study using the same methods and design, but a different group of test subjects, to show whether the results of the original study can be independently confirmed. Ideally, a study is replicated in a laboratory that differs from the one in which the work was originally completed. If the observed effect has been reported in a single study only or shown only in studies from a single research laboratory, then there is less confidence that the results are valid and not due to the influence of other extraneous factors. Unfortunately, until recently, the importance of replication has not been sufficiently recognized. The increasing difficulty in replicating scientific observations in many fields, however, has led to calls to increase scrutiny of the reproducibility of research study findings [34]. In the case of air ions, few experimental animal studies exist in any one topic area, with the exceptions of studies on behavior, learning and memory, and serotonin and other neurotransmitters. Within these topic areas, however, the available studies have often addressed disparate endpoints, making the direct comparison of results across studies difficult. Even among the studies that measured one endpoint—levels of the neurotransmitter serotonin—they are not easily compared. Some of these studies looked at levels of serotonin within brain tissues (or within specific regions of the brain) [57, 58, 75, 76], while others looked at levels within the blood [72–74, 75–81, 114]. In two studies, the precursor or degradation products of serotonin were examined [57, 79], and one study examined the responsiveness of tissues to applied serotonin [79].

Overall, the most carefully controlled studies of air ion exposures were those conducted by Bailey and Charry [57, 78, 82] and Creim et al. [59, 64]. In the studies of Bailey and Charry, the study investigators used a specially-designed ion exposure system based on corona discharge that had been previously evaluated and tested for the uniformity of ion concentrations, current density, and electric fields, and the production of noise, ozone, and other gaseous by-products. Controls were sham exposed to either ambient air or a positive or negative static electric field (to address the potential effects of the electric field alone). All behavioral parameters were measured using automated systems and with the investigators blinded as to the exposure status of the animals until after the experiment was completed. In the studies of air ions on serotonin concentrations, the animals were sacrificed at the same time of day to avoid circadian effects. Further, the experimental design was counter-balanced to prevent any systematic differences from affecting the experimental outcome of the study. Although a single concentration of air ions was tested, multiple exposure durations were implemented to investigate potential duration-response relationships. In these studies, exposure to either positive or negative air ions had no effect on the measured parameters, including locomotor and rearing activity, as well as brain levels of serotonin, norepinephrine, or dopamine. The main limitation of these studies is that while the group sizes were 14–17 subjects for most analyses and the group sizes were greater than many other studies reviewed, they did not have sufficient power to detect very small effects.

In the studies of Creim et al. [59, 64], exposures were again conducted using a corona discharge ion generator system described in detail by Weigel et al. [131]. Controls were exposed to positive or negative static electric fields of varying field strengths, and exposed animals were treated with the same electric fields in the presence of air ions of varying concentrations to address potential dose–response relationships. Uniformity of the exposures was assessed based on measurements of the electric field taken at various locations within the exposure apparatus. The amounts of ozone, light, noise, and air flow produced were also carefully measured, and noise conditions were replicated with sham exposures. All parameters were measured using automated systems and the investigators were blinded to the exposure status of the animals. In these studies, air ion exposures had no effect on the amount of time animals spent in exposure or sham treatment compartments of a shuttle box apparatus and were not associated with a learned taste aversion.

## Conclusions

In summary, a systematic review of the experimental animal studies of air ion exposure finds that much of the research in this arena is relatively old, and very little new research on this topic has been pursued in recent years. Further, many of the studies suffer from various reporting and methodological deficiencies which pose a serious risk of bias. These include the absence of sham exposure for study controls, and potential investigator bias due to the lack of blinded analyses, and failure to control for the influence of confounding variables associated with the operation of the air ion generating system (i.e., the presence of an electric field, the production of ozone and other gaseous by-products, noise, and light). As an indication of the importance of poor quality and systematic bias in affecting the outcome of the studies reviewed, studies that were rated as low quality and a high potential for bias also tended to report more findings supported by a modest or strong statistical strength of evidence.

Additionally, few studies tested for effects at more than one air ion concentration or exposure duration to investigate possible dose–response relationships. Within each research topic and across all topics, there was no visible evidence of any dose–response relationships. The well-controlled studies, however, consistently reported no effects of exposure on any of the health endpoints examined. In conclusion, the available experimental animal studies do not provide reliable evidence for any biological response, adverse or beneficial, and thus do not provide a basis to conclude that air ions per se are toxic. This conclusion is only tempered by the low statistical power of many studies reviewed, which may have led to the underreporting of potential air ion effects. Overall, the conclusion is consistent with those of recent comprehensive reviews and meta-analyses of human experimental studies addressing air ions and effects on mood and behavior and respiratory function [6, 14].

The animal studies provide no responsive or supportive data about the potential therapeutic effects of exposures to very high concentrations of negative air ions on depression. To test such hypotheses, the best research approach would be to test for a reduction in depression in human clinical trials of suitable size, design, and quality control. The animal data reviewed here do not indicate any potential for toxicity from air ion exposure. To the extent that any additional animal research is warranted to test for therapeutic effects on depression, it should be performed only in a model animal system for which there is empirical evidence that the test system can reliably distinguish agents that have been found to be beneficial in treating human depression from agents that have non-specific behavioral test profiles.

## Additional files

**Additional file 1.** Tabular summaries of study characteristics and conclusions.

**Additional file 2.** Forest plots: quantitative differences between exposed and control groups.

**Additional file 3.** Evaluation of study quality and statistical strength of evidence.

## Abbreviations

5-HT: 5-Hydroxytryptamine
AMP: adenosine monophosphate
ARRIVE: Animals in Research Reporting In Vivo Experiments
CI: confidence interval
CO_2_: carbon dioxide
DNA: deoxyribonucleic acid
EEG: electroencephalogram
PD: proportional difference
SMD: standardized mean difference
TS-1: undefined anti-tumor drug
WNI: water-generated negative air ions
