# Supplementary material for: Exposure of laboratory animals to small air ions: a systematic review of biological and behavioral studies
Source: Biomed Eng Online. 2018 Jun 5;17:72. doi: 10.1186/s12938-018-0499-z (PMC5987445; doi:10.1186/s12938-018-0499-z)
Supplement: Supplementary file 1 — Additional file 1. Tabular summaries of study characteristics and conclusions. [file 12938_2018_499_MOESM1_ESM.pdf]

Additional File 1: Tables S1-S10. Tabular summaries of study characteristics and conclusions

Table S1	Experimental animal studies of exposure to air ions and behavior
Table S2	Experimental animal studies of exposure to air ions and learning and memory
Table S3	Experimental animal studies of exposure to air ions and serotonin or other neurotransmitters
Table S4	Experimental animal studies of exposure to air ions and tracheal function
Table S5	Experimental animal studies of exposure to air ions and respiratory infection
Table S6	Experimental animal studies of exposure to air ions and cardiovascular function
Table S7	Experimental animal studies of exposure to air ions and reproduction and growth
Table S8	Experimental animal studies of exposure to air ion exposure and carcinogenesis
Table S9	Experimental animal studies of exposure to air ions and other health endpoints
Table S10	Mean values − air ion tracheal function studies

Table S1 Experimental animal studies of exposure to air ions and behavior

Study	Species, Strain, Sex, n	Exposures	Source for Ion Generation	Sham exposures Used?	Confounders Addressed?	Blinded / Randomized?	Reported Findings
Herrington and Smith, 1935 [47]	Rat, unknown, M, 8	1,200,000 negative air ions/cm^3, ~23 hours/day, 300 days (Group exposed)	Corona discharge system	Y	• Electric field – No<br>• Ozone, gaseous by-products – No<br>• Noise – No<br>• Light – No	No/No	• No effect on wheel running at <175 days; Increased wheel running in exposed rats after >175 days<br>• Visible light from ion source noted
Bachman et al., 1966 [56]	Rat, Sprague Dawley, M, 11	100,000-2,680,000 negative air ions/cm^3; 100,000-4,900,000 positive air ions/cm^3, 45 minutes (Group exposed)	Kr-85 ion generator	Yes	• Electric field – No<br>• Ozone, gaseous by-products – N/A<br>• Noise – N/A<br>• Light – N/A	No/Yes	• Exposure-related increase in motor activity to both ion polarities<br>• Indicators of fear (urination and defecation) differed with exposure to positive and negative air ions (described but not analyzed)<br>• DATA NOT EXTRACTED
Olivereau and Lambert, 1981 [48]	Rat, unknown, M, 12	Experiment 3 750,000 positive or negative air ions/cm^3, 20 minutes (Group exposed)	Corona discharge system	Yes	• Electric field – Yes<br>• Ozone, gaseous by-products – Yes<br>• Noise – Yes<br>• Light – No	No/No	• Small reductions in spontaneous activity of rats exposed to positive air ions<br>• Small increase in spontaneous activity of rats exposed to negative air ions

Study	Species, Strain, Sex, n	Exposures	Source for Ion Generation	Sham exposures Used?	Confounders Addressed?	Blinded / Randomized?	Reported Findings
Olivereau et al., 1981 [49]	Rat, Wistar, M, 6	80,000 positive or negative air ions/cm^3, 3 weeks (Group exposed)	Corona discharge system	Yes	• Electric field – No<br>• Ozone, gaseous by-products – Yes<br>• Noise – No<br>• Light – No	Yes/No	• At low brain stimulation intensities, both positive and negative air ions reduced brain electrical activity in reticulocortical region of rats' brains<br>• At high brain stimulation intensities, positive air ions reduced brain electrical activity in reticulocortical region<br>• At high brain stimulation intensities, brain electrical activity in reticulocortical region was similar in control and negative air ion-treated animals<br>• The EEG measures are interdependent and are responses to brain stimulation. Thus, results are not independent of one another despite separate exposures of animals.
Lambert et al., 1981 [50]	Rat, Wistar, M, 6	80,000 positive or negative air ions/cm^3, 3 weeks	Corona discharge system	Yes	• Electric field – No<br>• Ozone, gaseous by-products – Yes<br>• Noise – No<br>• Light – No	Yes/No	• Increased amplitude of EEG readings in the rat brain frontal and occipital regions with positive air ions<br>• Increased amplitude and reduced frequency of theta hippocampal rhythm with positive air ions<br>• No significant effect of negative air ions
Beardwood et al., 1986, (Beardwood et al., 1987)* [53, 58]	Rat, Long-Evans, M, 5-8	700,000 positive air ions/cm^3, 4+ days (Group exposed)	Corona discharge system	Yes	• Electric field – No<br>• Ozone, gaseous by-products – No<br>• Noise – No<br>• Light – No	No/No	• Delayed response of rats to noxious stimuli<br>• Response concluded to involve the serotonergic system, but not the opioid signaling pathway<br>• DATA NOT EXTRACTED
Bailey and Charry, 1986 [78]	Rat, Sprague-Dawley (outbred Holtzman), M, 6-19	500,000 positive or negative air ions/cm^3, 2-66 hours	Corona discharge system	Yes	• Electric field – Yes<br>• Ozone, gaseous by-products – Yes<br>• Noise – Yes<br>• Light – No	Yes/Yes†	• No effect on locomotor or rearing activity of rats<br>• Controlled and well-characterized exposure set-up described in Charry et al., 1986<br>• DATA NOT EXTRACTED

Study	Species, Strain, Sex, n	Exposures	Source for Ion Generation	Sham exposures Used?	Confounders Addressed?	Blinded / Randomized?	Reported Findings
Lambert and Olivereau, 1987 [55]	Rat, Wistar, M, 6	80,000 positive or negative air ions/cm^3, 3 weeks (Group exposed)	Corona discharge system	Yes	• Electric field – No<br>• Ozone, gaseous by-products – Yes<br>• Noise – No<br>• Light – No	Yes/Yes	• Altered sleep patterns in rats with both treatments
Lenkiewicz et al., 1989 [52]	Hamster, Syrian Waterhouse, M, 14	14,000 negative air ions/cm^3, 10-30 minutes	Corona discharge system (BION 78-Medicor minitype ionizer)	Yes	• Electric field – No<br>• Ozone, gaseous by-products – No<br>• Noise – No<br>• Light – No	No/No	• Altered motor activity in hamsters<br>• The smell of ozone was reported
Beardwood and Jordi, 1990; (Beardwood et al., 1987)* [54, 58]	Rat, Long-Evans, M, 6-18	700,000 negative air ions/cm^3, 4+ days (Group exposed)	Corona discharge system	Yes	• Electric field – No<br>• Ozone, gaseous by-products – No<br>• Noise – No<br>• Light – No	No/Yes	• No effect on response of rats to noxious stimuli<br>• Reduced analgesic effect of morphine
Dabrowska et al., 1990 [51]	Mouse, CBA inbred, M, 10	14,000 negative air ions/cm^3, 10-40 minutes/day for 14 days	Corona discharge system (BION BJ6 Minitype ionizer)	Yes	• Electric field – No<br>• Ozone, gaseous by-products – No<br>• Noise – No<br>• Light – No	No/Yes	• Increased exploratory activity and reduced fear with 20-minute exposures; similar findings with 30-minute exposures, but to a lesser extent<br>• Minimal effects with 10- and 40-minute exposures
Creim et al., 1993 [59]	Rat, Long-Evans, M, 20-40	<2,000 positive or negative air ions/cm^3, 10,000 positive or negative air ions/cm^3, or 250,000 positive or negative air ions/cm^3, 60 minutes	Corona discharge system	Yes	• Electric field – Yes<br>• Ozone, gaseous by-products – Yes<br>• Noise – Yes<br>• Light - Yes	No/No	• No effect of air ions on response to static electric fields as to amount of time rats spent in exposure or sham compartment of shuttle box apparatus<br>• Assessed multiple air ion exposure levels<br>• Used a carefully controlled and well-characterized exposure system described in Weigel et al., 1987<br>• DATA NOT EXTRACTED

Study	Species, Strain, Sex, n	Exposures	Source for Ion Generation	Sham exposures Used?	Confounders Addressed?	Blinded / Randomized?	Reported Findings
Livanova et al., 1999 [60]	Rat, Wistar, M, 5-6	31.6/second negative air ions (concentration not reported), 60 minutes (Group exposed)	Corona discharge system	No	• Electric field – No<br>• Ozone, gaseous by-products – No<br>• Noise – No<br>• Light – No	No/No	• Prevention of acute stress response in immobilized rats<br>• No effect on non-immobilized animals<br>• DATA NOT EXTRACTED

* Data appear to be from Beardwood et al., 1986 and were not extracted separately.
† Confirmed by author.

Table S2 Experimental animal studies of exposure to air ions and learning and memory

Study	Species, Strain, Sex, n	Exposures	Source for Ion Generation	Sham exposures Used?	Confounders Addressed?	Blinded / Randomized?	Reported Findings
Bauer, 1955 [65]	Rat, derived from Wistar, M, 5-6	6,000 positive air ions/cm^3 or 3,000 negative air ions /cm^3, 36 days (Group exposed)	Polonium ion generator	No	• Electric field – No<br>• Ozone, gaseous by-products – N/A<br>• Noise – N/A<br>• Light – N/A	No/No	• No effect of air ion exposure on ability of rats submitted to electroconvulsive shock treatment to learn a water maze<br>• DATA NOT EXTRACTED
	Rat, derived from Wistar, M, 5-6	6,500 positive air ions or 3,000 negative air ions, 15 days (Group exposed)	Polonium ion generator	No	• Electric field – N/A<br>• Ozone, gaseous by-products – N/A<br>• Noise – N/A<br>• Light – N/A	No/No	• No effect of air ion exposure on ability of rats submitted to electroconvulsive shock treatment to build nests<br>• DATA NOT EXTRACTED
Jordan and Sokoloff, 1959 [62]	Rat, unknown, M, 75	8,000-9,000 negative air ions/cm^3, 3 hours/day,15 days (Group exposed)	Polonium ion generator	No	• Electric field – No<br>• Ozone, gaseous by-products – N/A<br>• Noise – N/A<br>• Light – N/A	No/No	• No effect on water maze performance of young rats with negative air ions, but young rats seemed slightly more excited and less coordinated<br>• Improved water maze performance of elderly rats with negative air ions
Duffee and Koontz, 1965 [66]	Rats, Wistar, M, 3	290,000 positive or 140,000 negative air ions/cm^3, 23+ hours/day, 33 days (Group exposed)	Krypton-85 ion generator	No	• Electric field – N/A<br>• Ozone, gaseous by-products – N/A<br>• Noise – N/A<br>• Light – N/A	Yes/Yes	• Exposure to air ions, particularly negative ions, improved maze performance of older rats<br>• DATA NOT EXTRACTED
Frey, 1967 [67]	Rat, Wistar, M, 10-18	Negative air ions producing body currents of 5 x 10^{-10} amperes, 60 minutes/day,13 days	Wesix Co, tritium generator	Yes	• Electric field – No<br>• Ozone, gaseous by-products – N/A<br>• Noise – N/A<br>• Light – N/A	Yes/No	• Ion-exposed rats showed less reduction in lever pressing for food when presented a tone previously paired with shocks to tail (inhibited the buzzer-induced conditioned emotional response)<br>• DATA NOT EXTRACTED

Study	Species, Strain, Sex, n	Exposures	Source for Ion Generation	Sham exposures Used?	Confounders Addressed?	Blinded / Randomized?	Reported Findings
Nazzaro et al., 1967 [68]	Rat, Sprague Dawley, M, 6	25,304 positive air ions/cm^3 or 31,349 negative air ions/cm^3, 8 days (Individual & group exposed)	Tritium ion generator	Yes (cross over)	• Electric field – No<br>• Ozone<br>• gaseous by-products –N/A<br>• Noise – N/A<br>• Light – N/A	No/Yes	• Inconclusive results regarding effects of air ions on anxiety and lever-pressing<br>• DATA NOT EXTRACTED
Terry et al., 1969 [69]	Rat, King-Holtzman hybrid, M/F, 10	7,000,000 negative air ions/cm^3 and 70,000,000 negative air ions/cm^3, duration not reported (Group exposed)	Beta ion generator (Dynamic Ionaire Mark VII ion generator)	Yes	• Electric field – No<br>• Ozone, gaseous by-products –<br>• Noise – N/A<br>• Light – No	No/Yes	• No effect on errors or time completing water maze in female rats<br>• Male rats exposed to negative air ions made significantly less errors in a water maze<br>• DATA NOT EXTRACTED
Falkenberg and Kirk, 1977 [70]	Rat, Sprague-Dawley, M, 10	100,000 positive or negative air ions/cm^3, 2-hour periods, 4 days	Corona discharge system (Philco ion generator)	No	• Electric field – No<br>• Ozone, gaseous by-products – No<br>• Noise – No<br>• Light – No	No/Yes	• Rats exposed to negative air ions exhibited greater avoidance performance than rats exposed to positive air ions<br>• DATA NOT EXTRACTED
Olivereau and Lambert, 1981 [48]<br><br>(Experiment 2 was reported in Lambert and Olivereau, 1980 [63] as well)	Rat, unknown, M, 60	Experiment 1: 600,000-650,000 positive or negative air ions/cm^3, 30 minutes (Group exposed)	Corona discharge system	Yes	• Electric field – Yes<br>• Ozone, gaseous by-products – Yes<br>• Noise – Yes<br>• Light – No	No/No	• Reduced time to escape and success in escaping a noxious stimulus in mice with negative air ions<br>• Increased time to escape and success in escaping a noxious stimulus in mice with positive air ions

Study	Species, Strain, Sex, n	Exposures	Source for Ion Generation	Sham exposures Used?	Confounders Addressed?	Blinded / Randomized?	Reported Findings
	Rat, unknown, M, 6-7	Experiment 2: 80,000 positive or negative air ions/cm^3, 3 weeks	Corona discharge system	Yes	• Electric field – Yes<br>• Ozone, gaseous by-products – No<br>• Noise – Yes<br>• Light – No	No/Yes	• Reduced step-through latency in successive passive avoidance retention tests in rats with exposure; response greater with positive ions than with negative air ions<br>• Increased activity index and exploratory behavior with negative air ions<br>• Reduced activity index with positive air ions
Creim et al., 1995 [64]	Rat, SPF Long-Evans, M, 14	200,000 positive or negative air ions/cm^3, 4 hours/day with + 75/kV/m or – 75 kV/m electric field	Corona discharge system	Yes	• Electric field – Yes<br>• Ozone, gaseous by-products – Yes<br>• Noise – Yes<br>• Light –Yes	No/Yes	• No learned taste aversion in rats associated with either positive or negative air ions and concomitant static electric field exposure<br>• Used a carefully controlled and well-characterized exposure system described in Weigel et al., 1987

Table S3 Experimental animal studies of exposure to air ions and serotonin or other neurotransmitters

Study	Species, Strain, Sex, n	Exposures	Source for Ion Generation	Sham exposures Used?	Confounders Addressed?	Blinded / Randomized?	Reported Findings
Krueger and Smith, 1960 [71]	Mouse; NAMRU; M/F, 4<br>Guinea pig, M/F M/F, 4	Negative air ions (concentrations not reported), 1-14+ hours (Group exposed)	Tritium ion generator	Yes	• Electric field – No<br>• Ozone, gaseous by-products – N/A<br>• Noise – N/A<br>• Light – N/A	No/No	• Negative air ions reduced serotonin levels in tracheal tissue<br>• Effect of negative air ions on 5-HIAA excretion in urine of guinea pigs, was inconclusive
Krueger et al., 1963 [72]	Mouse, NAMRU, M, 20	4,500 positive air ions/cm^3 or 51,000 positive air ions/cm^3, 7 days (Group exposed)	Tritium ion generator and Krypton-84 ion generator	No	• Electric field – No<br>• Ozone, gaseous by-products – N/A<br>• Noise – N/A<br>• Light – N/A	No/Yes	• Increased blood serotonin concentrations in mice after 3 days of positive air ion exposure<br>• Response thought to be associated with exposure to CO_2 positive air ions<br>• Results confounded by significant illness in animals exposed to CO_2 positive air ions
Krueger et al., 1966 [73]	Mouse, NAMRU, M, 10	430,000 positive air ions/cm^3 or 500,000 positive air ions/cm^3, 12-23 days (Group exposed)	Tritium ion generator	No (not within same experiment)	• Electric field – No<br>• Ozone, gaseous by-products – N/A<br>• Noise – N/A<br>• Light – N/A	No/Partial[§§]	• Increase in blood serotonin at 48 hours in mice exposed to 430,000 positive ions/cm^3 in 2% CO_2; smaller response at 14 days with exposure to 500,000 ions/cm^3<br>• In 4% CO_2, the drop in blood serotonin levels observed in the controls was overcome and peaked at 10 days of treatment<br>• Reliability of differences not assessed by statistical analyses
Krueger et al., 1968 [74]	Mouse, NAMRU, M, 50-80	400,000-500,000 positive or negative air ions/cm^3, 1-12 days (Group exposed)	Tritium ion generator	No	• Electric field – No<br>• Ozone, gaseous by-products – N/A<br>• Noise – N/A<br>• Light – N/A	No/No	• Increased blood serotonin concentrations in mice with positive air ions<br>• Reduced blood serotonin concentrations in mice with negative air ions<br>• Overlapping confidence intervals of exposed and control mice indicate no reliable effect of ion exposure

[§§] Some mice allocated randomly; others matched by weight.

Study	Species, Strain, Sex, n	Exposures	Source for Ion Generation	Sham exposures Used?	Confounders Addressed?	Blinded / Randomized?	Reported Findings
Krueger and Kotaka, 1969 [75]	Mouse, NAMRU, M, 30-56	2,000-4,000 positive or negative air ions/cm^3, 30,000-40,000 positive or negative air ions/cm^3, or 350,000-500,000 positive or negative air ions/cm^3, 12-72 hours (Group exposed)	Tritium ion generator	No	• Electric field – Yes<br>• Ozone, gaseous by-products – N/A<br>• Noise – N/A<br>• Light – N/A	No/Yes	• Reduced brain serotonin concentrations in mice with both positive and negative air ions
Gilbert, 1973 [77]	Rat, Sprague-Dawley, M, 5-6	30,000 negative air ions/cm^3, 8 hours/day intermittent or continuous	Tritium ion generator	No	• Electric field – No<br>• Ozone, gaseous by-products – N/A<br>• Noise – N/A<br>• Light – N/A	No/Yes	• Reduced handling reactions in individually-housed rats<br>• Reduced brain serotonin concentrations
Diamond et al., 1980 [76]	Rat, Long-Evans, M, 8-18	100,000 negative air ions/cm^3, 21 days (Group exposed)	Corona discharge system	No	• Electric field – No<br>• Ozone, gaseous by-products – No<br>• Noise – No<br>• Light – No	Yes/No	• Increased brain serotonin concentrations in rats
Charry and Bailey, 1985 [82]	Rat, Sprague-Dawley (outbred Holtzman), M, 6-14	500,000 positive or negative air ions/cm^3, 2-66 hours	Corona discharge system	Yes	• Electric field – Yes<br>• Ozone, gaseous by-products – Yes<br>• Noise – Yes<br>• Light – No	Yes/Yes†	• No effect on brain regional concentrations of norepinephrine or dopamine in rats<br>• Controlled and well-characterized exposure set-up described in Charry et al., 1986

† Confirmed by author.

Study	Species, Strain, Sex, n	Exposures	Source for Ion Generation	Sham exposures Used?	Confounders Addressed?	Blinded / Randomized?	Reported Findings
Dowdall and DeMontigny, 1985 [79]	Rat, Sprague-Dawley, M, 17-80	1,500,000 positive or negative air ions/cm^3, 21 days (Group exposed)	Corona discharge system	Yes	• Electric field – No<br>• Ozone, gaseous by-products – No<br>• Noise – No<br>• Light – No	Yes/No	• No effect on brain serotonin, tryptophan, or 5-hydroxyindoleacetic acid concentrations in rats<br>• No effect on blood serotonin or plasma tryptophan concentrations<br>• No effect on CA1 and CA3 pyramidal neuron responsiveness to norepinephrine or acetylcholine<br>• Increased (negative air ions) or decreased (positive air ions) CA1 and CA3 pyramidal neuron responsiveness to serotonin
Kellogg et al., 1985a,1985b; Kellogg and Yost, 1986 [80, 81,114]	Mouse, NAMRU, F, 4-47	2,000 positive or negative air ions/cm^3 or 200,000 positive or negative air ions/cm^3, 2 years (Group exposed)	Tritium ion generator	No	• Electric field – Yes<br>• Ozone, gaseous by-products – N/A<br>• Noise – N/A<br>• Light – N/A	No/No	• No effect on blood serotonin concentrations in mice with positive or negative air ions
Beardwood et al., 1987 [58]	Rat, Long-Evans, M, 8-19	700,000 positive or negative air ions/cm^3, 7 days (Group exposed)	Corona discharge system	No	• Electric field – No<br>• Ozone, gaseous by-products – No<br>• Noise – No<br>• Light – No	No/Yes	• Increased whole brain serotonin concentrations in rats with positive air ion exposure; no effect on lung serotonin concentrations<br>• Reduced whole brain and lung concentrations in rats with negative air ion exposure
Bailey and Charry, 1987 [78]	Rat, Sprague-Dawley (outbred Holtzman), M, 6-15	500,000 positive or negative air ions/cm^3, 2-66 hours	Corona discharge system	Yes	• Electric field – Yes<br>• Ozone, gaseous by-products – Yes<br>• Noise – Yes<br>• Light – No	Yes/Yes†	• No effect on brain regional concentrations of serotonin or serotonin turnover in rats<br>• Controlled and well-characterized exposure set-up described in Charry et al., 1986

† Confirmed by author.

Table S4 Experimental animal studies of exposure to air ions and tracheal function

Study	Species, Strain, Sex, n (range)	Exposres	Source for Ion Generation	Sham exposures Used?	Confounders Addressed?	Blinded / Randomized?	Reported Findings
Krueger and Smith, 1958 [86]	Mouse, NAMRU/aguouti, M/F, 10; rat, unspecified, M/F, 20; rabbit; unspecified, M/F; 20	1 x 10^9 positive or negative air ions/cm^2/second (concentrations not reported), 15-20 minutes (exposure before and after tracheotomy) (Group exposed)	Tritium ion generator	Yes (cross over)	• Electric field – Yes<br>• Ozone, gaseous by-products – N/A<br>• Noise – N/A<br>• Light – N/A	No/No	• Positive air ions reduced ciliary rate and mucus flow, and caused vascular irritability and muscle contraction in tracheal tissues of mice, rats, and rabbits<br>• Negative air ions increased ciliary rate and mucus flow, and caused muscle relaxation in tracheal tissues of mice, rats, and rabbits<br>• DATA NOT EXTRACTED
Krueger and Smith, 1958 [87]	Rabbit, unknown, unknown, unknown	Negative and positive air ions (concentrations not reported), 15-20 minutes (anesthetized) (Group exposed)	Tritium ion generator	Yes (cross over)	• Electric field – No<br>• Ozone, gaseous by-products – N/A<br>• Noise – N/A<br>• Light – N/A	No/No	• Negative air ions increased ciliary rate in O_2 enriched atmosphere but not in N_2, or CO_2 enriched atmospheres<br>• Positive air ions decreased ciliary rate in CO_2 enriched atmosphere but not in N_2 or O_2 enriched atmospheres<br>• DATA NOT EXTRACTED
Krueger and Smith, 1959 [88]	Mouse, NAMRU, M/F, 6	1 x 10^7 positive or negative air ions/cm^2/ second (concentrations not reported), 10 minutes to 3 days (Group exposed)	Tritium ion generator	Yes (cross over)	• Electric field – No<br>• Ozone, gaseous by-products – N/A<br>• Noise – N/A<br>• Light – N/A	No/No	• Effects on mouse tracheal function present for at least 4 weeks post-exposure to air ions<br>• Effects on mouse tracheal function present with as little as 20 minutes of air ion exposure<br>• DATA NOT EXTRACTED

Study	Species, Strain, Sex, n (range)	Exposres	Source for Ion Generation	Sham exposures Used?	Confounders Addressed?	Blinded / Randomized?	Reported Findings
Andersen, 1972 [96]	Rabbit; unspecified, unspecified, unspecified, 5-6	200,000 positive air ions/cm^3, 190,000 negative air ions/cm^3 or bipolar (200,000 negative + 210,000 positive ion/cm^3), 90 minutes	Tritium generator	Yes (cross over)	• Electric field – Yes<br>• Ozone, gaseous by-products – N/A<br>• Noise – NA<br>• Light – N/A	No/No	• No effect on ciliary beat frequency or mucous flow rates (visual analysis)<br>• Sensitivity of ciliary beat frequency to temperature and humidity<br>• DATA NOT EXTRACTED
Sirota et al., 2006 [97]	Rat, Wistar, M, 3-5	25,000-600,000 negative air ions/cm^3, 30 minutes daily for 1-8 days (Group exposed)	Corona discharge system (Elion 131M, Elion 132S, Elion 132R)	Yes	• Electric field – No<br>• Ozone, gaseous by-products – Yes<br>• Noise – No<br>• Light – No	No/No	• Lustre ionizer (100,000-600,000 negative air ions/cm^3) caused histological damage to trachea, reduced SOD, GPx, GSH reductase and NADPH oxidative enzyme activities and increased phagocytic blood cell activity<br>• Bioionizer (50,000 and 500,000 negative air ions/cm^3), Elion-132S (320,000 negative air ions/cm^3, 60 min) and Elion-132R (500,000 negative air ions/cm^3, 4 days) did not damage trachea<br>• DATA NOT EXTRACTED
Sirota et al., 2008 [98]	Rat, Wistar, M/F, unspecified	320,000-350,000 negative air ions/cm^3, 60 minutes (Group exposed)	Corona discharge system (Elion 132Sh)	No	• Electric field – No<br>• Ozone, gaseous by-products – Yes<br>• Noise – No<br>• Light – No	No/No	• No histological damage to trachea<br>• Increased protein secretion in lavage mucous<br>• Spontaneous production of reactive oxygen species (ROS) by lavage cells unaffected<br>• Increased response of female, but not male, blood cells to ROS stimulant<br>• Superoxide dismutase and glutathione reductase lower in blood lysate of males, but not females<br>• DATA NOT EXTRACTED

Table S5 Experimental animal studies of exposure to air ions and respiratory infection

Study	Species, Strain, Sex, n	Exposures	Source for Ion Generation	Sham exposures Used?	Confounders Addressed?	Blinded/Randomized?	Reported Findings
Krueger and Levine, 1967 [99]	Mouse, NAMRU, F, 10 (assembled into groups of 40)	300,000-400,000 positive air ions/cm^3, 30+ days (Grouped exposed)	Tritium ion generator	Yes	• Electric field – No<br>• Ozone, gaseous by-products – N/A<br>• Noise – N/A<br>• Light – N/A	No/Yes	• Increased mortality of mice due to Coccidioidomycosis infection with treatment<br>• Reduced fungal particles in pulmonary tissues with treatment and no difference in histopathology<br>• No effect of treatment applied before or much later after infection
Krueger et al., 1970 [100]	Mouse, NAMRU, F, 10 (Assembled into groups of 40-196)	100,000-410,000 positive air ions/cm^3, ≤16 days (Grouped exposed)	Tritium ion generator	Yes	• Electric field – No<br>• Ozone, gaseous by-products – N/A<br>• Noise – N/A<br>• Light – N/A	No/Yes	• Increased mortality of mice due to bacterial (K. Pneumoniae) and viral (influenza) pulmonary infection with treatment
Krueger et al., 1971 [101]	Mouse, NAMRU, F, 10, (Assembled into groups of 220-237)	200,000-500,000 negative air ions/cm^3, ≤11 days (Grouped exposed)	Tritium ion generator	Unknown	• Electric field – No<br>• Ozone, gaseous by-products – N/A<br>• Noise – N/A<br>• Light – N/A	No/No	• No effect on mortality from influenza infection with treatment

Study	Species, Strain, Sex, n	Exposures	Source for Ion Generation	Sham exposures Used?	Confounders Addressed?	Blinded/Randomized?	Reported Findings
Krueger and Reed, 1972 [102]	Mouse, NAMRU/Swiss HaM/1 cr., F, 10, (Assembled into groups of 40-215)	2,700 positive air ions/cm^3, 17,000 positive air ions/cm^3, 3,500 negative air ions/cm^3, 20,000 negative air ions/cm^3, 220,000-370,000 negative air ions/cm^3, 2,000-3,500 mixed air ions/cm^3, or ion-depleted air, 12-13 days (Grouped exposed)	Tritium ion generator, corona discharge system	Yes	• Electric field – No/No<br>• Ozone, gaseous by-products – No/Yes<br>• Noise – N/A;/No<br>• Light – N/A;/No	No/No	• Increased mortality from influenza in mice at low positive and negative air ion concentrations, mid-range positive air ion concentrations, and in ion-depleted air<br>• No effect on mortality rates from influenza at mid-range negative air ion concentrations<br>• Reduced mortality rates from influenza at high negative air ion concentrations and at background air ion concentrations of clean outdoor air
Krueger et al., 1974 [103]	Moue, SPF NAMRU, F, 90-220	2,700-5,000 positive or negative air ions/cm^3, 230,000-500,000 positive or negative air ions/cm^3, or ion-depleted air, 11+ days (Grouped exposed)	Tritium ion generator	Yes	• Electric field – Yes<br>• Ozone, gaseous by-products – N/A<br>• Noise – N/A<br>• Light – N/A	No/No	• No effect of low or high positive or negative air ion concentrations or ion-depleted air on mortality in mice from influenza aerosol exposure (no measures of variability or any statistical analysis)

Table S6 Experimental animal studies of exposure to air ions and cardiovascular function

Study	Species, Strain, Sex, n (range)	Exposures	Source for Ion Generation	Sham exposures Used?	Confounders Addressed?	Blinded / Randomized?	Reported Findings
Bachman et al., 1965 [106}	Rat, unspecified, M, 17	531,000 positive air ions/cm^3 or 510,000 negative air ions/cm^3, 30 minutes	Krypton-85 ion generator	Yes	• Electric field – No<br>• Ozone, gaseous by-products – N/A<br>• Noise – N/A<br>• Light – N/A	No/No	• Increased heart rate in rats with positive and negative air ions<br>• Increased respiration rate with positive ions; no effect of negative air ions
McDonald et al., 1965 [107]	Rat, unspecified, M, 17	350,000 positive air ions/cm^3 or 360,000 negative air ions/cm^3, 30 minutes (inhalation of air ions blocked)	Krypton-85 ion generator	Yes	• Electric field – No<br>• Ozone, gaseous by-products – N/A<br>• Noise – N/A<br>• Light – N/A	No/No	• Reduced heart rate in rats with positive air ions; no effect of negative air ions<br>• Possible increase in respiration rate after exposure to negative air ions; no effect with positive ions<br>• Responses could not have been produced by inhalation of air ions because exposure was limited to the rest of the body
Ju and Kubo, 1997 [108]	Rat, SHR, M/F, 30	200 negative air ions/cm^3, 8 weeks	Tourmaline ionizer	No	• Electric field – N/A<br>• Ozone, gaseous by-products – N/A<br>• Noise – N/A<br>• Light – N/A	No/No	• Decreased blood pressure in rats<br>• Decreased low frequency and increased high frequency of the heart rate variability power spectrum
Suzuki et al., 2008 [109]	Rat, Wistar, M, 4-6	5,000-8,000 negative air ions/cm^3, 60 minutes	Water ion generator	Yes	• Electric field – No<br>• Ozone, gaseous by-products – N/A<br>• Noise – No<br>• Light – N/A	No/No	• Decreased mean blood pressure and heart rate in rats<br>• Increased high frequency in the heart rate variability power spectrum in rats<br>• No effect on vagotomized rats<br>• Increased *c-fos* expression in PVN and LC, reduced *c-fos* expression in NA

Table S7 Experimental animal studies of exposure to air ions and reproduction and growth

Study	Species, Strain, Sex, n	Exposures	Source for Ion Generation	Sham exposures Used?	Confounders Addressed?	Blinded / Randomized?	Reported Findings
Herrington and Smith, 1935 [47]	Rat, unknown, M, 8	1,200,000 negative air ions/cm^3, ~23 hours/day, 300 days	Corona discharge system	Yes	• Electric field – No<br>• Ozone, gaseous by-products – No<br>• Noise – No<br>• Light – No	No/No	• No effect on body weight, growth, or hemoglobin concentrations<br>• Visible light from ion source noted.<br>• DATA NOT EXTRACTED
Hinsull et al., 1981 [117]	Rat, inbred WAB, M, unclear	10,000 positive or negative air ions/cm^3, gestation to adulthood (Group exposed)	Corona discharge system	No	• Electric field – No<br>• Ozone, gaseous by-products – No<br>• Noise – No<br>• Light – No	No/Yes	• No effect on embryonic development or post-weaning growth in rats<br>• No effect of positive air ions on neonatal development<br>• Reduced neonatal survival with negative air ions<br>• Results confounded by presence of respiratory disease in colony<br>• DATA NOT EXTRACTED
Hinsull et al., 1983 [118]	Rat, inbred WAB, M/F n not specified	10,000 negative air ions/cm^3, two generations of animals, 20 weeks (Group exposed)	Corona discharge system	No	• Electric field – No<br>• Ozone, gaseous by-products – No<br>• Noise – No<br>• Light – No	No/No	• No effect on reproduction or growth of rats in F_2 or F_3 generations<br>• Lower thymus weights in exposed rats in F_2 generation<br>• No effect on thymus weights in F_3 generation<br>• No effect on adrenal weights in F_2 for F_3 generations<br>• DATA NOT EXTRACTED
Hinsull et al., 1984 [111]	Rat, inbred WAB, M/F, 20	10,000 positive air ions/cm^3, two generations of animals, 20 weeks (Group exposed)	Corona discharge system	No	• Electric field – No<br>• Ozone, gaseous by-products – No<br>• Noise – No<br>• Light – No	No/No	• No effect on reproduction or growth of rats over two generations of animals

Study	Species, Strain, Sex, n	Exposures	Source for Ion Generation	Sham exposures Used?	Confounders Addressed?	Blinded / Randomized?	Reported Findings
Kellogg et al., 1985a,1985b; Kellogg and Yost, 1986 [80, 81,114]	Mouse, NAMRU, F, 4-48	2,000 positive or negative air ions/cm^3 or 200,000 positive or negative air ions/cm^3, 2 years (Group exposed)	Tritium ion generator	Yes	• Electric field – Yes<br>• Ozone, gaseous by-products – N/A<br>• Noise – N/A<br>• Light – N/A	No/No	• No effect on body weights, organ weights (liver, kidney, spleen, adrenals), blood serotonin, or serum globulin levels in mice<br>• Reduced serum glucose concentrations in mice with positive and negative air ions; no dose response<br>• Effects on serum cholesterol, urea nitrogen concentrations with positive and negative air ions in first year of study; not seen in second year of study<br>• Reduced survival with positive and negative air ions; no dose-response<br>• Results confounded by mild vitamin deficiency and serious intestinal infection in colony
Hinsull and Head, 1986 [113]	Rat, inbred WAB, M/F, 20	10,000 positive ions/cm^3, four generations of animals (Group exposed)	Corona discharge system	Yes	• Electric field – No<br>• Ozone, gaseous by-products – No<br>• Noise – No<br>• Light – No	No/No	• No effect on reproduction or growth of rats over two generations of animals
Hinsull, 1988 [112]	Rat, inbred WAB, M/F, 25	10,000 negative air ions/cm^3, lifetime exposure starting at 5 weeks of age (Group exposed)	Corona discharge system	Yes	• Electric field – No<br>• Ozone, gaseous by-products – No<br>• Noise – No<br>• Light – No	No/No	• No effect on growth of rats over lifetime of animals<br>• Suggested increased lifespan with exposure
Yamamoto et al., 2014 [115]	Rat, Crl:CD (SD), F, 10	8,500,000 air ions/cm^3 of both polarities simultaneously, 6 hours/day, during 20 days of pregnancy (Group exposed)	Bipolar corona discharge system	Yes	• Electric field – No<br>• Ozone, gaseous by-products – Yes<br>• Noise – No<br>• Light - No	No/Yes	• No effect on body weight, food consumption or clinical signs of dams<br>• No effect on embryo-fetal development (12 parameters) and fetal morphology (20 parameters)

Study	Species, Strain, Sex, n	Exposures	Source for Ion Generation	Sham exposures Used?	Confounders Addressed?	Blinded / Randomized?	Reported Findings
Yamamoto et al., 2015 [116]	Rat, Crl:CD (SD), M, F, 24	7,900,000 ions/cm^3 of both polarities simulataneously for 10 weeks + through weaning of parents and offspring (Grouped exposed)	Bipolar corona Discharge system	Yes	• Electric field – No<br>• Ozone, gaseous by-products – No<br>• Noise – No<br>• Light - No	No/Yes	• Increased thyroid, pituitary, uterus weights in parental rats (incidental)<br>• No effects in first generation offspring except increased food consumption of day 20 of gestation but not other days<br>• No effects on reproduction, development or organ histology of two generations

Table S8 Experimental animal studies of exposure to air ion exposure and carcinogenesis

Study	Species, Strain, Sex, n	Exposures	Source for Ion Generation	Sham exposures Used?	Confounders Addressed?	Blinded / Randomized?	Reported Findings
Yamada et al., 2006 [119]	Mouse, unknown, sex not known, 10	Negative air ions, quantity and duration not clearly reported (Group exposed)	Water ion generator and corona discharge system	No	• Electric field – No<br>• Ozone, gaseous by-products – No<br>• Noise – No<br>• Light – No	No/No	• Enhanced cytotoxic NK cell response<br>• Inhibited carcinogenesis<br>• Results confounded by unclear reporting of exposures
Takasawa et al., 2011 [120]	Rat, Crl:CD (SD), M, 5<br>Mouse, Crlj:CD-I (ICR), M, 5	Negative and positive air ions at 1,420,000, 5,630,000 and 7,520,000 ions/cm^3, 48 hours	Bipolar corona discharge system	Yes	• Electric field – No<br>• Ozone, gaseous by-products – No<br>• Noise – No<br>• Light – No	No/Yes	• No change in body weight, clinical signs,<br>• No DNA damage (comet assay tail length and % DNA) in lung or blood<br>• No histopathological abnormalities in lung

Table S9 Experimental animal studies of exposure to air ions and other health endpoints

Study	Species, Strain, Sex, n (range)	Exposures	Source for Ion Generation	Sham exposures Used?	Confounders Addressed?	Blinded / Randomized?	Reported Findings
Wehner et al., 1983 [123]	Rat, Sprague-Dawley, M, 10	Negative air ions (concentrations not reported), 90-140 minutes	Electro aerosol generator	Yes, but not exposed to water aerosol (only air exposed)	• Electric field – No<br>• Ozone, gaseous by-products – N/A<br>• Noise – No<br>• Light – N/A	No/Yes	• No effect on 9 hematologic parameters, 12 serum clinical chemistry<br>• No effect on CSF calcium concentrations or pH<br>• Variable exposure period
Jaśkowski and Myśliwski, 1986 [126]	Rat, Wistar, M/F, 10	2 x 10^{10} positive air ions/ second or 5 x 10^{11} negative air ions/second (concentrations not reported), 3 hours	BION 80 ion generator	Yes	• Electric field – No<br>• Ozone, gaseous by-products – No<br>• Noise – No<br>• Light – No	No/No	• Skin wounds on rats healed more quickly with negative air ions and more slowly with positive air ions<br>• DATA NOT EXTRACTED
Bordas and Deleanu, 1989 [124]	Rat, “mixed,” M, 5	12,000-15,000 negative air ions/cm^3, 10-15 days (Group exposed)	Corona discharge system	No	• Electric field – No<br>• Ozone, gaseous by-products – No<br>• Noise – No<br>• Light – No	No/No	• Improved healing of ulcers in rats with prophylactic and therapeutic treatment
Deleanu and Bordas,1991 [125]	Rat, “mixed,” M, 5	12,000-15,000 negative air ions/cm^3, 10-15 days (Group exposed)	Corona discharge system	No	• Electric field – No<br>• Ozone, gaseous by-products – Yes<br>• Noise – No<br>• Light – No	No/No	• Improved healing of ulcers in rats with prophylactic and therapeutic treatment (Duplicate publication of same data in Bordas and Deleanu, 1989).<br>• Reduction of anterior pituitary and adrenal gland weights with prophylactic treatment; reduction of anterior pituitary and adrenal gland weight with reduction in fascicular cortex thickness and nuclear volume with prophylactic plus therapeutic treatment

Table S10 Mean values, air ions tracheal function studies

Study	Outcome	Special Conditions; Species	Exposure Duration	Time of measurement following exposure initiation	Exposure Type (in order of exposure)	Control Mean	Exposure Mean
Krueger and Smith, 1958 [87]	Mucus flow (mm/min)	Tracheotomized; rabbit	15-20 min	180 min	Pos	2.0*	0
	Ciliary Rate (beats/min)	Tracheotomized; rabbit	15-20 min	180 min	Pos	1000*	484
	Ciliary Rate (beats/min)	Tracheotomized; rat	15-20 min	80 min<br>40 min	Pos<br>Neg	900<br>900	250<br>952
	Ciliary Rate (beats/min)	Tracheotomized; rabbit	15-20 min	60 min<br>60 min<br>60 min	Pos<br>Neg<br>Pos	958<br>961<br>959	602<br>1043<br>603
	Ciliary Rate (beats/min)	Tracheotomized; mouse	15-20 min	30 min<br>30 min<br>30 min<br>30 min	Pos<br>Neg<br>Pos<br>Neg	1002<br>947<br>997<br>998	755<br>1193<br>0<br>1255
	Respirations (per min)	1 mg chlorpromazine hydrochloride, tracheotomized; rat	15-20 min	30 min<br>30 min<br>30 min<br>30 min<br>30 min<br>30 min<br>30 min<br>30 min	Neg<br>Pos<br>Neg<br>Pos<br>Neg<br>Pos<br>Neg<br>Pos	76*	61<br>74<br>14<br>72<br>60<br>90<br>59<br>74
	Ciliary Rate (beats/min)	Tracheotomized; mouse	15-20 min	120 min	Pos	897	602
	Ciliary Rate (beats/min)	Tracheotomized; mouse	15-20 min	120 min	Neg	897	1148
Krueger and Smith, 1959 [88]	Ciliary Rate (beats/min)	mouse	3 days	4 wks	Pos	900	600
	Ciliary Rate (beats/min)	mouse	3 days	4 wks	Neg	900	1100
	Ciliary Rate (beats/min)	mouse	24 hours	30 min	Neg	850	950
	Ciliary Rate (beats/min)	mouse	24 hours	60 min	Neg	850	1050
	Ciliary Rate (beats/min)	mouse	24 hours	10 min	Pos	850	850
	Ciliary Rate (beats/min)	mouse	24 hours	20 min	Pos	850	600
	Ciliary Rate (beats/min)	mouse	24 hours	30 min	Pos	850	0

Study	Outcome	Special Conditions; Species	Exposure Duration	Time of measurement following exposure initiation	Exposure Type (in order of exposure)	Control Mean	Exposure Mean
	Ciliary Rate (beats/min)	mouse	24 hours	60 min	Pos	850	600
Krueger and Smith, 1959 [88]	Ciliary Rate (beats/min)	mouse	24 hours	120 min	Pos	850	0
Krueger and Smith, 1960 [71]	5-HT in respiratory tract (µg/g)	mouse	14 hours	4 days	Neg	5.0	3.1
	5-HT in respiratory tract (µg/g)	mouse	Continuous	4 days	Neg	5.4	3.3
	5-HIAA (µg/day)	No previous (-) exposure; Guinea pig	24 hours	24 hours	Neg<br>Neg	151*<br>118*	172<br>95
	5-HIAA (µg/day)	No previous (-) exposure; Guinea pig	24 hours	24 hours	Neg<br>Neg	99*<br>73*	187<br>99
	5-HIAA (µg/day)	No previous (-) exposure; Guinea pig	24 hours	24 hours	Neg<br>Neg	104*<br>131*	221<br>125
	5-HIAA (µg/day)	Previous (-) exposure; Guinea pig	24 hours	24 hours	Neg<br>Neg	119*<br>104*	119<br>111
Sirota et al., 2006 [97]	SOD activity (units/min/mg)	Lustre	30 min	Unspecified	Neg	5604	2737
	SOD activity (units/min/mg)	Lustre	30 min/day (8 days)	Unspecified	Neg	5604	2585
	Phagocytic activity of blood (amplitude)	Lustre	30 min	Unspecified	Neg	0.52	1.49
	Phagocytic activity of blood (amplitude)	Lustre	30 min/day (5 days)	Unspecified	Neg	0.52	0.75
	Phagocytic activity of blood (amplitude)	Lustre	30 min/day (8 day)	Unspecified	Neg	0.52	0.38

* Indicates mean of baseline measurements for exposed animal(s) used as control.
