# Supplementary material for: Exposure of laboratory animals to small air ions: a systematic review of biological and behavioral studies
Source: Biomed Eng Online. 2018 Jun 5;17:72. doi: 10.1186/s12938-018-0499-z (PMC5987445; doi:10.1186/s12938-018-0499-z)
Supplement: Supplementary file 2 — Additional file 2. Forest plots: quantitative differences between exposed and control groups. [file 12938_2018_499_MOESM2_ESM.pdf]

Additional File 2: Figures S1 – S8

Quantitative differences between exposed and control groups

Figure S1	Standardized mean differences − air ion behavior studies
Figure S2	Standardized mean differences air ion − learning and memory studies
Figure S3	Standardized mean differences air ion − serotonin or other neurotransmitter studies
Figure S4	Mean proportional differences − air ion respiratory infection studies
Figure S5	Standardized mean differences − air ion cardiovascular function studies
Figure S6	Standardized mean differences − air ion reproduction and growth studies
Figure S7	Standardized mean differences − air ion carcinogenesis studies
Figure S8	Standardized mean differences air ion − other health endpoints studies

Abbreviations and notes specific to each table follow Figure S8.

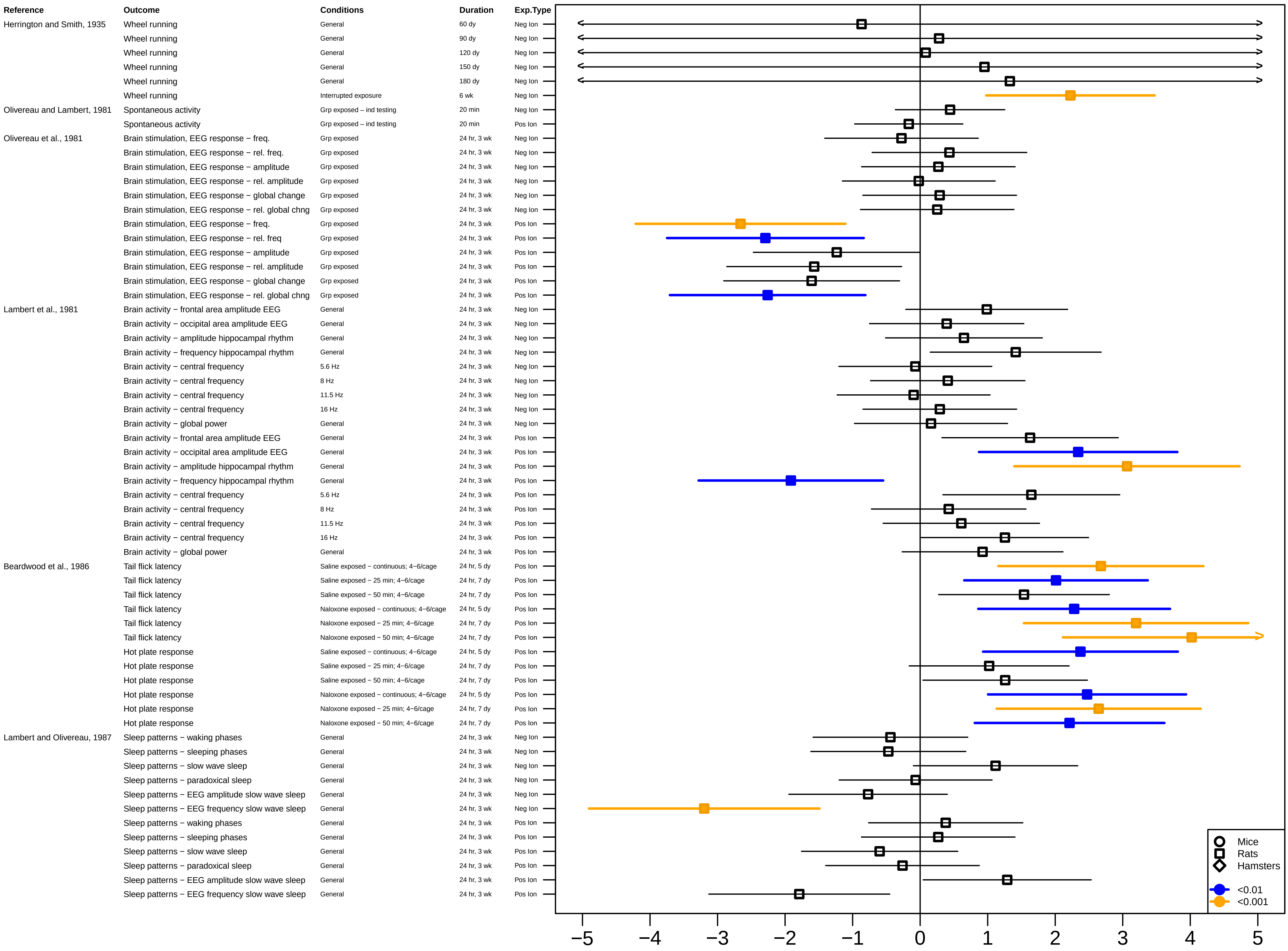

Figure S1. Standardized Mean Differences − Air Ion Behavior Studies

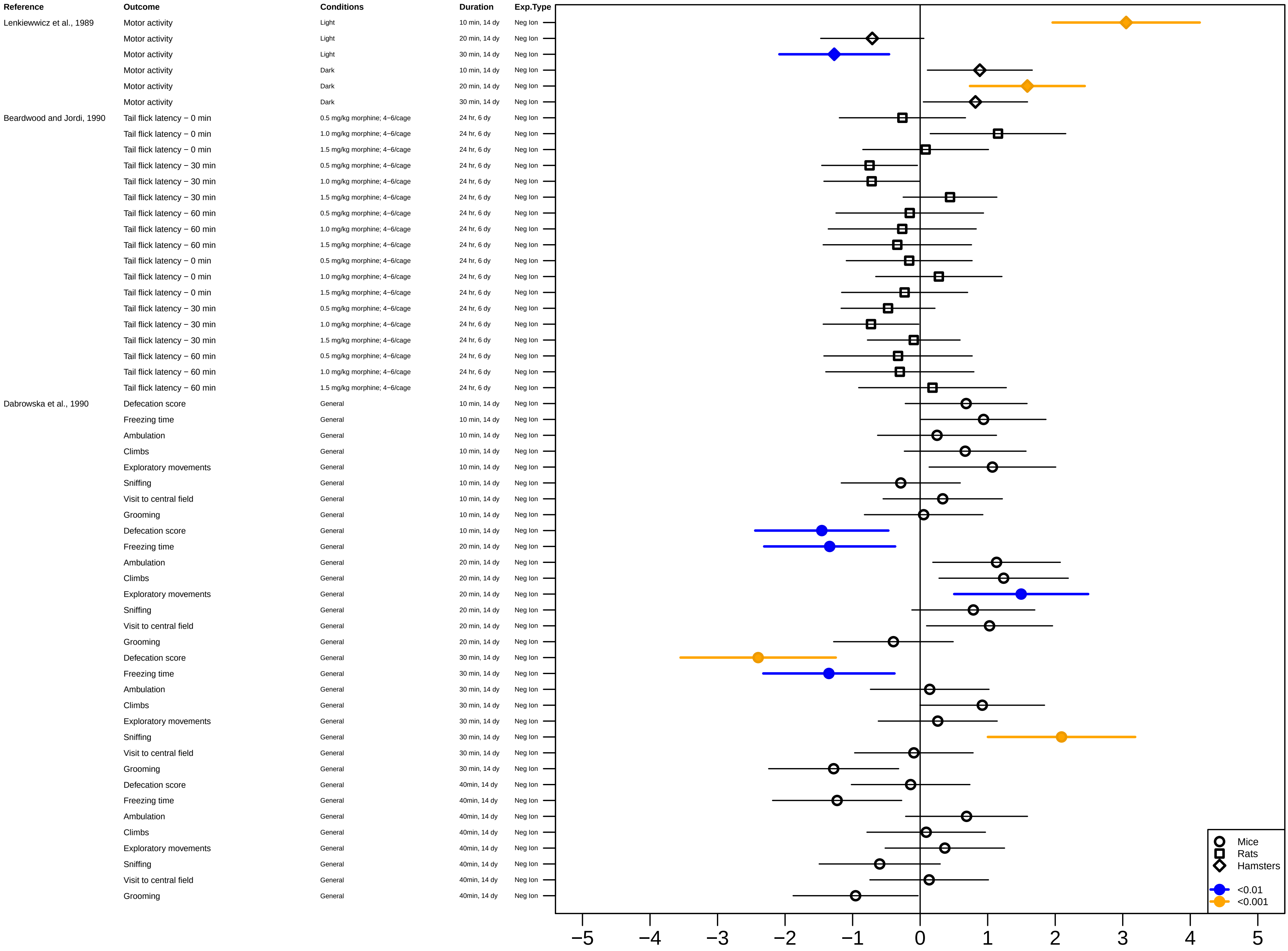

Figure S1 (Cont.)

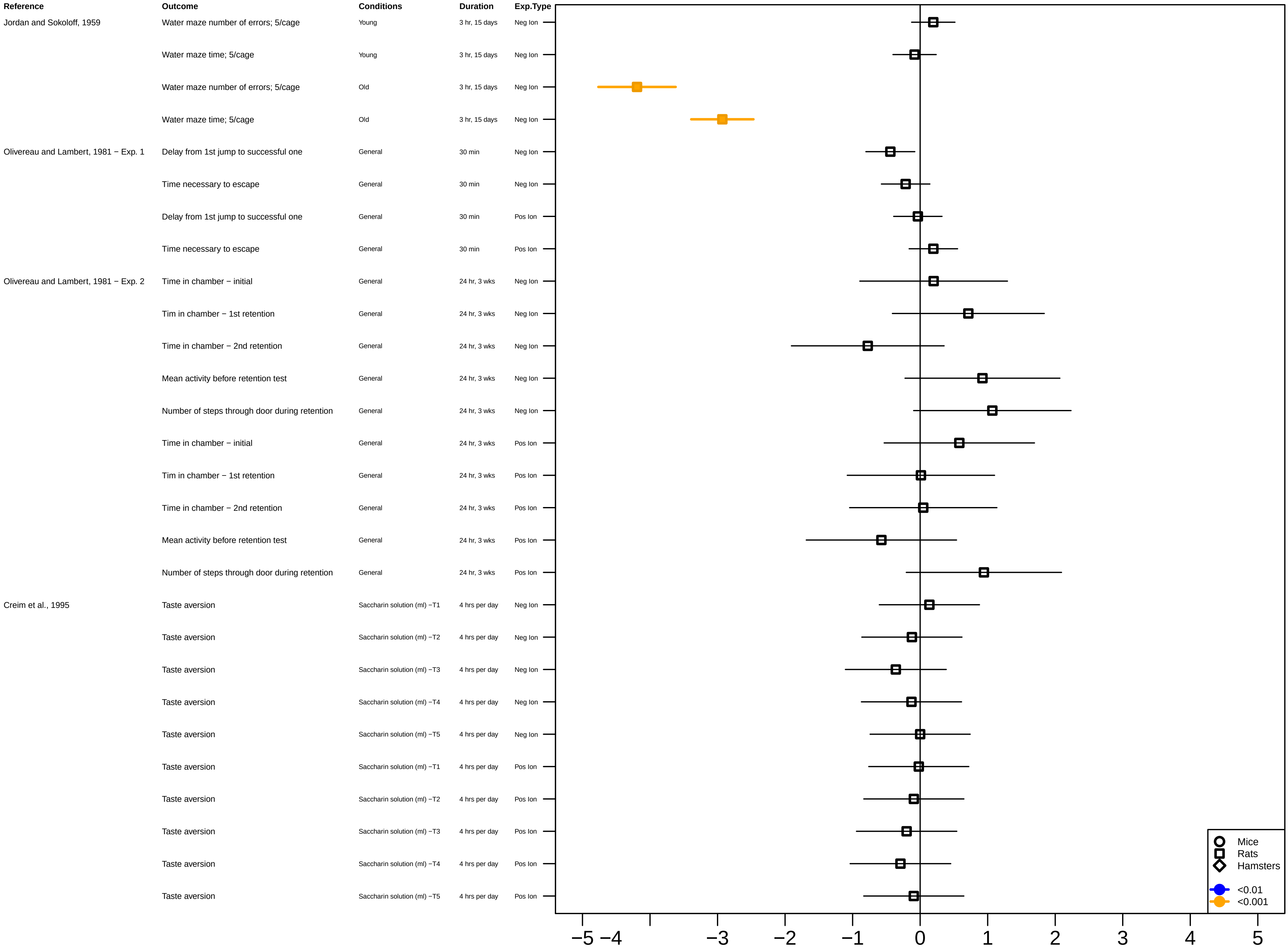

Figure S2. Standardized Mean Differences Air Ion − Learning and Memory Studies

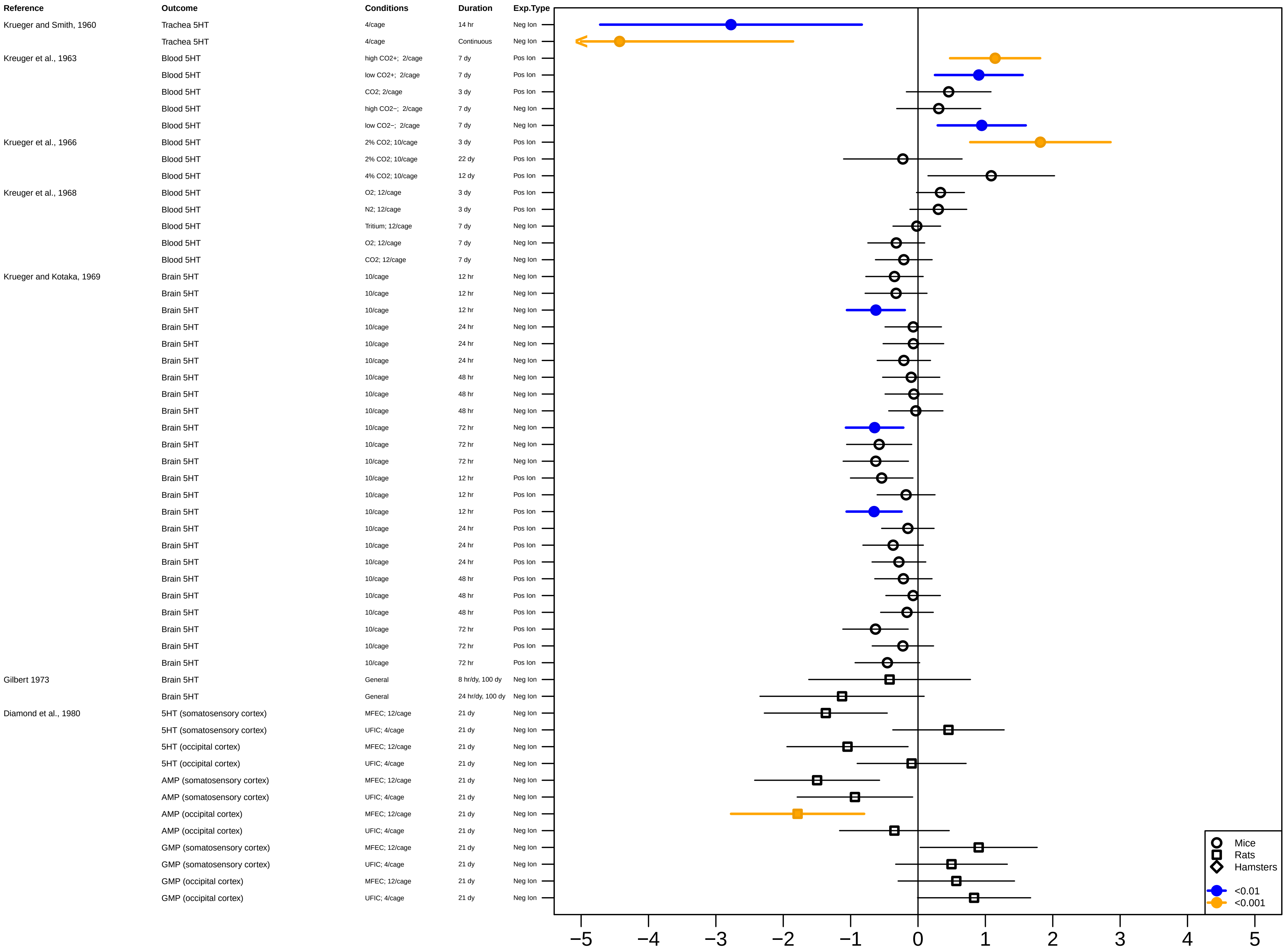

Figure S3. Standardized Mean Differences Air Ion − Serotonin or Other Neurotransmitter Studies

Reference	Outcome	Conditions	Duration	Exp.Type
Charry and Bailey, 1985	Dopamine: Hypothalamus	General	2 hr	Neg Ion
	Dopamine: Hypothalamus	AMPT Injection	2 hr	Neg Ion
	Dopamine: Brainstem	General	2 hr	Neg Ion
	Dopamine: Brainstem	AMPT Injection	2 hr	Neg Ion
	Dopamine: Hippocampus/amygdala	General	2 hr	Neg Ion
	Dopamine: Hippocampus/amygdala	AMPT Injection	2 hr	Neg Ion
	Dopamine: Frontal cortex	General	2 hr	Neg Ion
	Dopamine: Frontal cortex	AMPT Injection	2 hr	Neg Ion
	Dopamine: Striatum	General	2 hr	Neg Ion
	Dopamine: Striatum	AMPT Injection	2 hr	Neg Ion
	Dopamine: Hypothalamus	General	18 hr	Neg Ion
	Dopamine: Hypothalamus	AMPT Injection	18 hr	Neg Ion
	Dopamine: Brainstem	General	18 hr	Neg Ion
	Dopamine: Brainstem	AMPT Injection	18 hr	Neg Ion
	Dopamine: Hippocampus/amygdala	General	18 hr	Neg Ion
	Dopamine: Hippocampus/amygdala	AMPT Injection	18 hr	Neg Ion
	Dopamine: Frontal cortex	General	18 hr	Neg Ion
	Dopamine: Frontal cortex	AMPT Injection	18 hr	Neg Ion
	Dopamine: Striatum	General	18 hr	Neg Ion
	Dopamine: Striatum	AMPT Injection	18 hr	Neg Ion
	Dopamine: Hypothalamus	General	66 hr	Neg Ion
	Dopamine: Hypothalamus	AMPT Injection	66 hr	Neg Ion
	Dopamine: Brainstem	General	66 hr	Neg Ion
	Dopamine: Brainstem	AMPT Injection	66 hr	Neg Ion
	Dopamine: Hippocampus/amygdala	General	66 hr	Neg Ion
	Dopamine: Hippocampus/amygdala	AMPT Injection	66 hr	Neg Ion
	Dopamine: Frontal cortex	General	66 hr	Neg Ion
	Dopamine: Frontal cortex	AMPT Injection	66 hr	Neg Ion
	Dopamine: Striatum	General	66 hr	Neg Ion
	Dopamine: Striatum	AMPT Injection	66 hr	Neg Ion
	Norepinephrine: Hypothalamus	General	2 hr	Neg Ion
	Norepinephrine: Hypothalamus	AMPT Injection	2 hr	Neg Ion
	Norepinephrine: Brainstem	General	2 hr	Neg Ion
	Norepinephrine: Brainstem	AMPT Injection	2 hr	Neg Ion
	Norepinephrine: Hippocampus/amygdala	General	2 hr	Neg Ion
	Norepinephrine: Hippocampus/amygdala	AMPT Injection	2 hr	Neg Ion
	Norepinephrine: Frontal cortex	General	2 hr	Neg Ion
	Norepinephrine: Frontal cortex	AMPT Injection	2 hr	Neg Ion
	Norepinephrine: Hypothalamus	General	18 hr	Neg Ion
	Norepinephrine: Hypothalamus	AMPT Injection	18 hr	Neg Ion
	Norepinephrine: Brainstem	General	18 hr	Neg Ion
	Norepinephrine: Brainstem	AMPT Injection	18 hr	Neg Ion
	Norepinephrine: Hippocampus/amygdala	General	18 hr	Neg Ion
	Norepinephrine: Hippocampus/amygdala	AMPT Injection	18 hr	Neg Ion
	Norepinephrine: Frontal cortex	General	18 hr	Neg Ion
	Norepinephrine: Frontal cortex	AMPT Injection	18 hr	Neg Ion
	Norepinephrine: Hypothalamus	General	66 hr	Neg Ion
	Norepinephrine: Hypothalamus	AMPT Injection	66 hr	Neg Ion
	Norepinephrine: Brainstem	General	66 hr	Neg Ion
	Norepinephrine: Brainstem	AMPT Injection	66 hr	Neg Ion
	Norepinephrine: Hippocampus/amygdala	General	66 hr	Neg Ion
	Norepinephrine: Hippocampus/amygdala	AMPT Injection	66 hr	Neg Ion
	Norepinephrine: Frontal cortex	General	66 hr	Neg Ion
	Norepinephrine: Frontal cortex	AMPT Injection	66 hr	Neg Ion

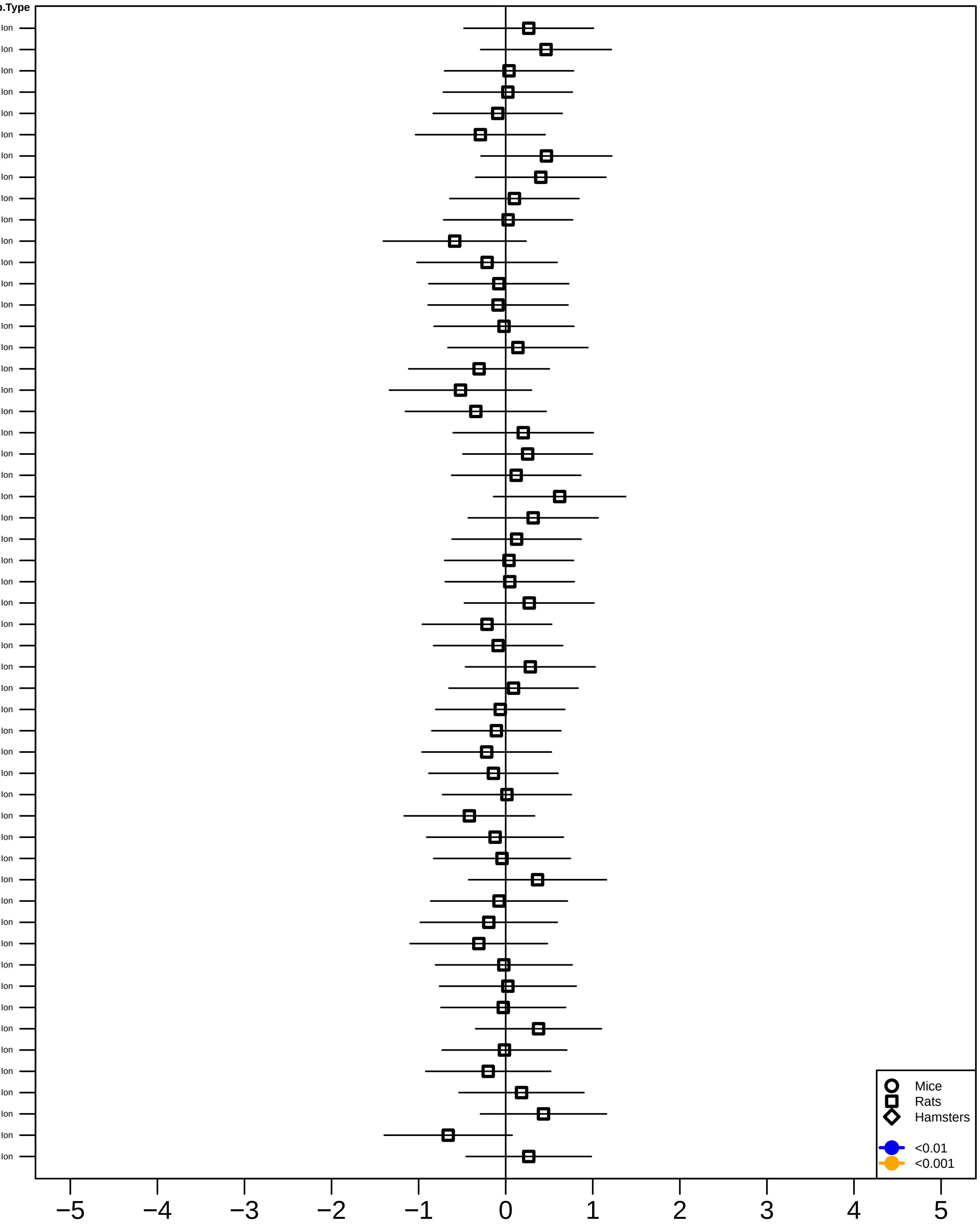

Figure S3 (Cont.)

Reference	Outcome	Conditions	Duration	Exp.Type
Charry and Bailey, 1985 (Cont.)	Dopamine: Hypothalamus	General	2 hr	Pos Ion
	Dopamine: Hypothalamus	AMPT Injection	2 hr	Pos Ion
	Dopamine: Brainstem	General	2 hr	Pos Ion
	Dopamine: Brainstem	AMPT Injection	2 hr	Pos Ion
	Dopamine: Hippocampus/amygdala	General	2 hr	Pos Ion
	Dopamine: Hippocampus/amygdala	AMPT Injection	2 hr	Pos Ion
	Dopamine: Frontal cortex	General	2 hr	Pos Ion
	Dopamine: Frontal cortex	AMPT Injection	2 hr	Pos Ion
	Dopamine: Striatum	General	2 hr	Pos Ion
	Dopamine: Striatum	AMPT Injection	2 hr	Pos Ion
	Dopamine: Hypothalamus	General	18 hr	Pos Ion
	Dopamine: Hypothalamus	AMPT Injection	18 hr	Pos Ion
	Dopamine: Brainstem	General	18 hr	Pos Ion
	Dopamine: Brainstem	AMPT Injection	18 hr	Pos Ion
	Dopamine: Hippocampus/amygdala	General	18 hr	Pos Ion
	Dopamine: Hippocampus/amygdala	AMPT Injection	18 hr	Pos Ion
	Dopamine: Frontal cortex	General	18 hr	Pos Ion
	Dopamine: Frontal cortex	AMPT Injection	18 hr	Pos Ion
	Dopamine: Striatum	General	18 hr	Pos Ion
	Dopamine: Striatum	AMPT Injection	18 hr	Pos Ion
	Dopamine: Hypothalamus	General	66 hr	Pos Ion
	Dopamine: Hypothalamus	AMPT Injection	66 hr	Pos Ion
	Dopamine: Brainstem	General	66 hr	Pos Ion
	Dopamine: Brainstem	AMPT Injection	66 hr	Pos Ion
	Dopamine: Hippocampus/amygdala	General	66 hr	Pos Ion
	Dopamine: Hippocampus/amygdala	AMPT Injection	66 hr	Pos Ion
	Dopamine: Frontal cortex	General	66 hr	Pos Ion
	Dopamine: Frontal cortex	AMPT Injection	66 hr	Pos Ion
	Dopamine: Striatum	General	66 hr	Pos Ion
	Dopamine: Striatum	AMPT Injection	66 hr	Pos Ion
	Norepinephrine: Hypothalamus	General	2 hr	Pos Ion
	Norepinephrine: Hypothalamus	AMPT Injection	2 hr	Pos Ion
	Norepinephrine: Brainstem	General	2 hr	Pos Ion
	Norepinephrine: Brainstem	AMPT Injection	2 hr	Pos Ion
	Norepinephrine: Hippocampus/amygdala	General	2 hr	Pos Ion
	Norepinephrine: Hippocampus/amygdala	AMPT Injection	2 hr	Pos Ion
	Norepinephrine: Frontal cortex	General	2 hr	Pos Ion
	Norepinephrine: Frontal cortex	AMPT Injection	2 hr	Pos Ion
	Norepinephrine: Hypothalamus	General	18 hr	Pos Ion
	Norepinephrine: Hypothalamus	AMPT Injection	18 hr	Pos Ion
	Norepinephrine: Brainstem	General	18 hr	Pos Ion
	Norepinephrine: Brainstem	AMPT Injection	18 hr	Pos Ion
	Norepinephrine: Hippocampus/amygdala	General	18 hr	Pos Ion
	Norepinephrine: Hippocampus/amygdala	AMPT Injection	18 hr	Pos Ion
	Norepinephrine: Frontal cortex	General	18 hr	Pos Ion
	Norepinephrine: Frontal cortex	AMPT Injection	18 hr	Pos Ion
	Norepinephrine: Hypothalamus	General	66 hr	Pos Ion
	Norepinephrine: Hypothalamus	AMPT Injection	66 hr	Pos Ion
	Norepinephrine: Brainstem	General	66 hr	Pos Ion
	Norepinephrine: Brainstem	AMPT Injection	66 hr	Pos Ion
	Norepinephrine: Hippocampus/amygdala	General	66 hr	Pos Ion
	Norepinephrine: Hippocampus/amygdala	AMPT Injection	66 hr	Pos Ion
	Norepinephrine: Frontal cortex	General	66 hr	Pos Ion
	Norepinephrine: Frontal cortex	AMPT Injection	66 hr	Pos Ion

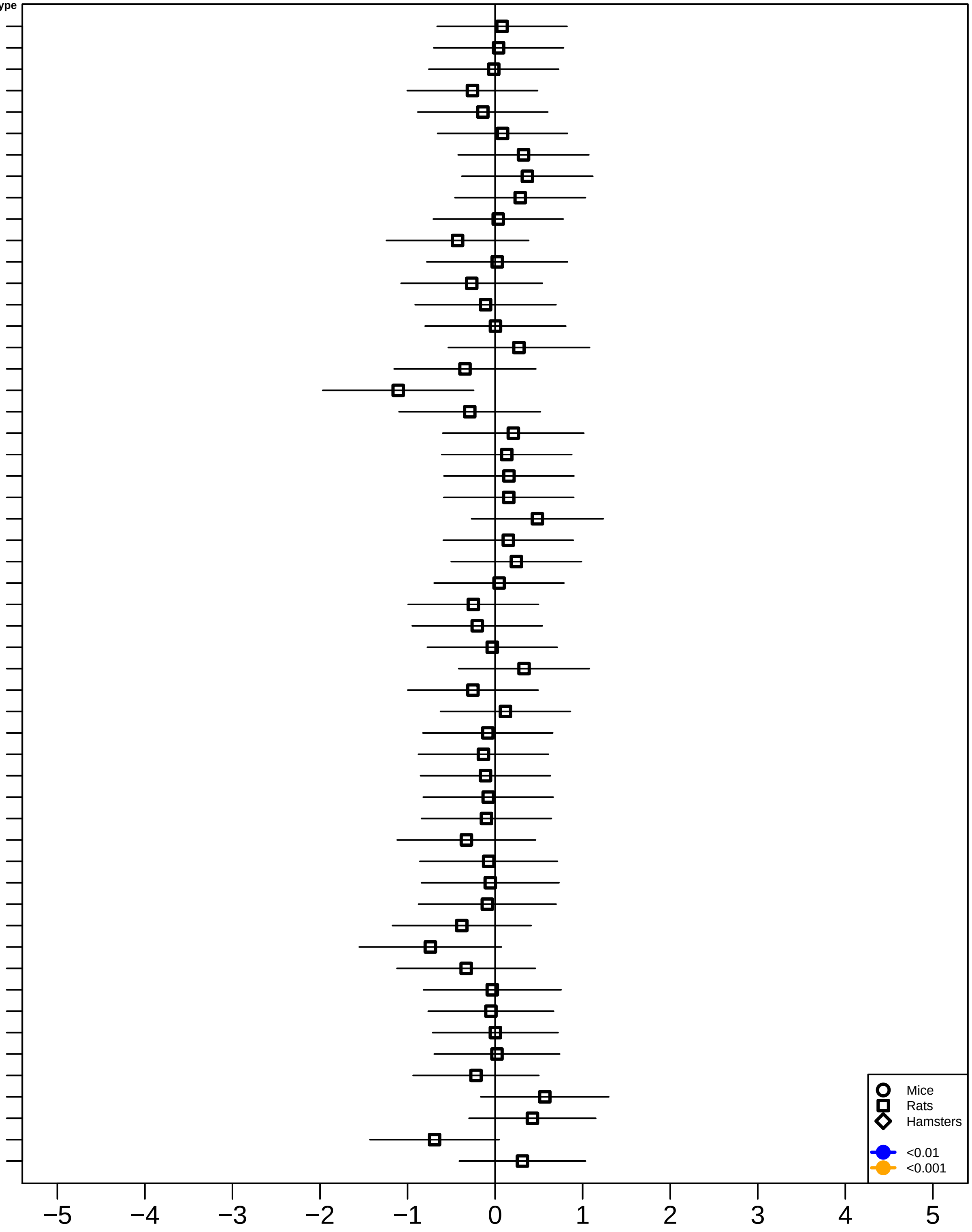

Figure S3 (Cont.)

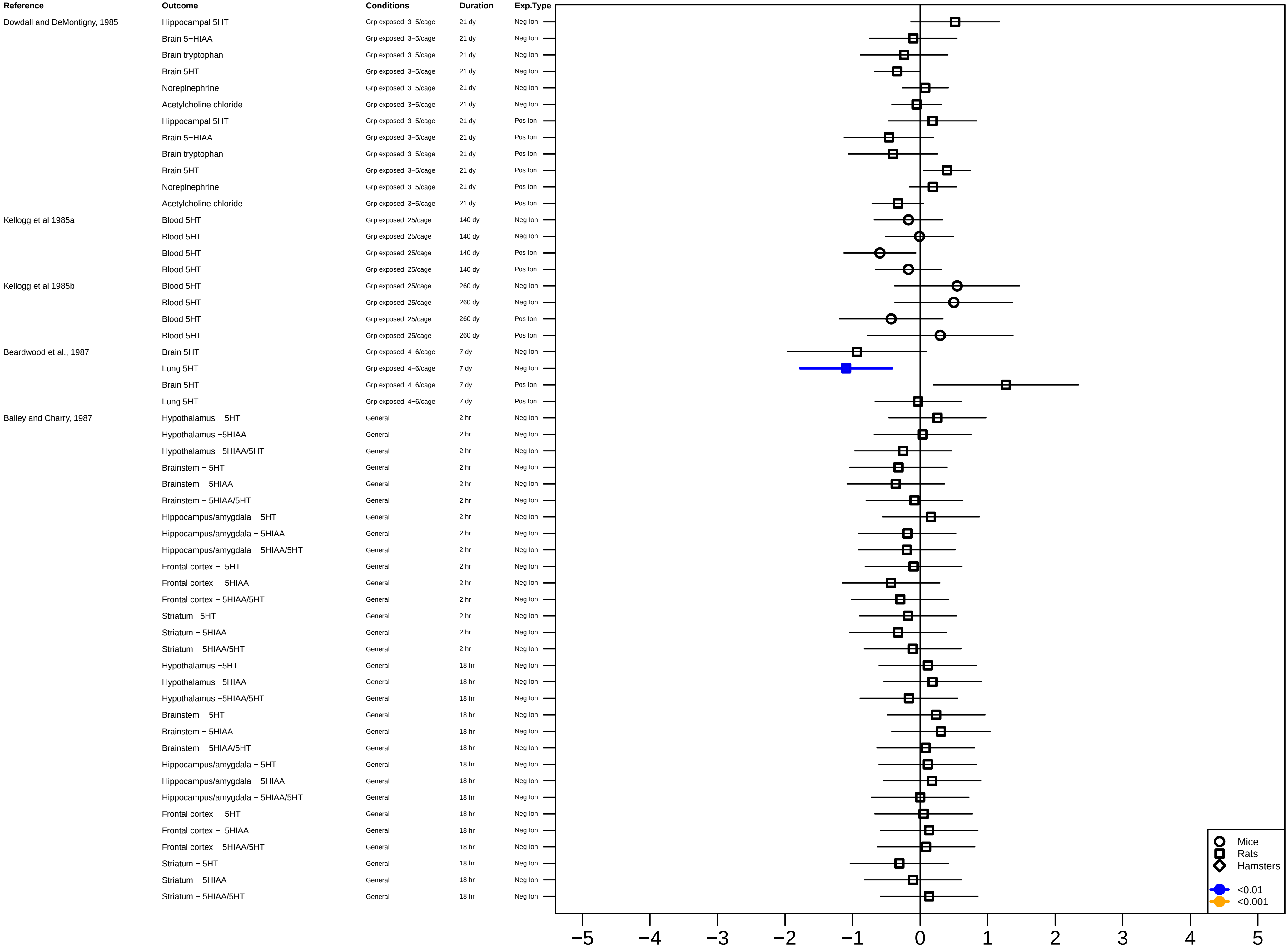

Figure S3 (Cont.)

Reference	Outcome	Conditions	Duration	Exp.Type
Bailey and Charry, 1987 (Cont.)	Hypothalamus −5HT	General	66 hr	Neg Ion
	Hypothalamus −5HIAA	General	66 hr	Neg Ion
	Hypothalamus −5HIAA/5HT	General	66 hr	Neg Ion
	Brainstem − 5HT	General	66 hr	Neg Ion
	Brainstem − 5HIAA	General	66 hr	Neg Ion
	Brainstem − 5HIAA/5HT	General	66 hr	Neg Ion
	Hippocampus/amygdala − 5HT	General	66 hr	Neg Ion
	Hippocampus/amygdala − 5HIAA	General	66 hr	Neg Ion
	Hippocampus/amygdala − 5HIAA/5HT	General	66 hr	Neg Ion
	Frontal cortex − 5HT	General	66 hr	Neg Ion
	Frontal cortex − 5HIAA	General	66 hr	Neg Ion
	Frontal cortex − 5HIAA/5HT	General	66 hr	Neg Ion
	Striatum − 5HT	General	66 hr	Neg Ion
	Striatum − 5HIAA	General	66 hr	Neg Ion
	Striatum − 5HIAA/5HT	General	66 hr	Neg Ion
	Hypothalamus −5HT	General	2 hr	Pos Ion
	Hypothalamus −5HIAA	General	2 hr	Pos Ion
	Hypothalamus −5HIAA/5HT	General	2 hr	Pos Ion
	Brainstem − 5HT	General	2 hr	Pos Ion
	Brainstem − 5HIAA	General	2 hr	Pos Ion
	Brainstem − 5HIAA/5HT	General	2 hr	Pos Ion
	Hippocampus/amygdala − 5HT	General	2 hr	Pos Ion
	Hippocampus/amygdala − 5HIAA	General	2 hr	Pos Ion
	Hippocampus/amygdala − 5HIAA/5HT	General	2 hr	Pos Ion
	Frontal cortex − 5HT	General	2 hr	Pos Ion
	Frontal cortex − 5HIAA	General	2 hr	Pos Ion
	Frontal cortex − 5HIAA/5HT	General	2 hr	Pos Ion
	Striatum − 5HT	General	2 hr	Pos Ion
	Striatum − 5HIAA	General	2 hr	Pos Ion
	Striatum − 5HIAA/5HT	General	2 hr	Pos Ion
	Hypothalamus −5HT	General	18 hr	Pos Ion
	Hypothalamus −5HIAA	General	18 hr	Pos Ion
	Hypothalamus −5HIAA/5HT	General	18 hr	Pos Ion
	Brainstem − 5HT	General	18 hr	Pos Ion
	Brainstem − 5HIAA	General	18 hr	Pos Ion
	Brainstem − 5HIAA/5HT	General	18 hr	Pos Ion
	Hippocampus/amygdala − 5HT	General	18 hr	Pos Ion
	Hippocampus/amygdala − 5HIAA	General	18 hr	Pos Ion
	Hippocampus/amygdala − 5HIAA/5HT	General	18 hr	Pos Ion
	Frontal cortex − 5HT	General	18 hr	Pos Ion
	Frontal cortex − 5HIAA	General	18 hr	Pos Ion
	Frontal cortex − 5HIAA/5HT	General	18 hr	Pos Ion
	Striatum − 5HT	General	18 hr	Pos Ion
	Striatum − 5HIAA	General	18 hr	Pos Ion
	Striatum − 5HIAA/5HT	General	18 hr	Pos Ion
	Hypothalamus −5HT	General	66 hr	Pos Ion
	Hypothalamus −5HIAA	General	66 hr	Pos Ion
	Hypothalamus −5HIAA/5HT	General	66 hr	Pos Ion
	Brainstem − 5HT	General	66 hr	Pos Ion
	Brainstem − 5HIAA	General	66 hr	Pos Ion
	Brainstem − 5HIAA/5HT	General	66 hr	Pos Ion
	Hippocampus/amygdala − 5HT	General	66 hr	Pos Ion
	Hippocampus/amygdala − 5HIAA	General	66 hr	Pos Ion
	Hippocampus/amygdala − 5HIAA/5HT	General	66 hr	Pos Ion
	Frontal cortex − 5HT	General	66 hr	Pos Ion
	Frontal cortex − 5HIAA	General	66 hr	Pos Ion
	Frontal cortex − 5HIAA/5HT	General	66 hr	Pos Ion
	Striatum − 5HT	General	66 hr	Pos Ion
	Striatum − 5HIAA	General	66 hr	Pos Ion
	Striatum − 5HIAA/5HT	General	66 hr	Pos Ion

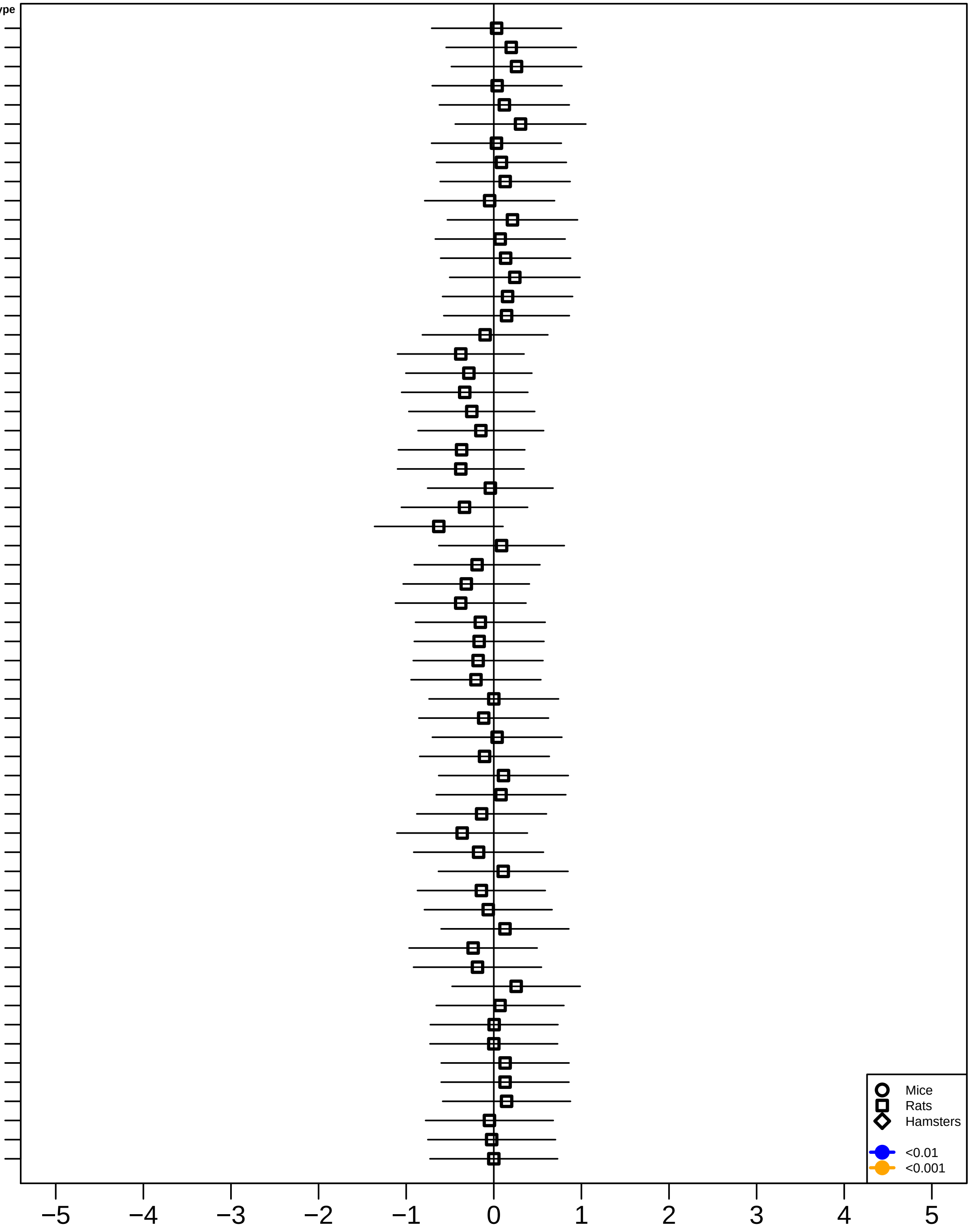

Figure S3 (Cont.)

Reference	Outcome	Conditions	Duration	Exp.Type
Krueger and Levine, 1967	Cumulative Mortality	23 strain Coccidioides Immitis; up to 12/cage	24 hr, 30 dy	Pos Ion
	Cumulative Mortality	21 strain Coccidioides Immitis; up to 12/cage	24 hr, 35 dy	Pos Ion
Krueger et al., 1970	Cumulative Mortality	Klebsiella pneumoniae; grp exposed	24 hr, 6 dy	Pos Ion
	Cumulative Mortality	PR8 strain of influenza virus; grp exposed	24 hr, 10 dy	Pos Ion
	Cumulative Mortality	PR8 strain of influenza virus; grp exposed	24 hr, 10 dy	Pos Ion
	Cumulative Mortality	PR8 strain of influenza virus; grp exposed	24 hr, 16 dy	Pos Ion
Krueger et al., 1971	Cumulative Mortality	Influenza virus; grp exposed	24 hr, 11 dy	Neg Ion
Krueger and Reed, 1972	Cumulative Mortality	PR8 strain of influenza virus; 10/cage; med neg	24 hr, 12 dy	Neg Ion
	Cumulative Mortality	PR8 strain of influenza virus; 10/cage; low neg	24 hr, 12 dy	Neg Ion
	Cumulative Mortality	PR8 strain of influenza virus; 10/cage; med neg	24 hr, 13 dy	Neg Ion
	Cumulative Mortality	PR8 strain of influenza virus; 10/cage; high neg	24 hr, 13 dy	Neg Ion
	Cumulative Mortality	PR8 strain of influenza virus; 10/cage; high pos	24 hr, 12 dy	Pos Ion
	Cumulative Mortality	PR8 strain of influenza virus; 10/cage; low pos	24 hr, 13 dy	Pos Ion
	Cumulative Mortality	PR8 strain of influenza virus; 10/cage; neg <70 and pos <90	24 hr, 12 dy	Dep
	Cumulative Mortality	PR8 strain of influenza virus; 10/cage; neg <75 and pos <100	24 hr, 12 dy	Dep
	Cumulative Mortality	PR8 strain of influenza virus; 10/cage; neg <60 and pos <100	24 hr, 13 dy	Dep
	Cumulative Mortality	PR8 strain of influenza virus; 10/cage	24 hr, 12 dy	Mix
Krueger et al., 1974	Cumulative Mortality	PR8 strain of influenza virus; 10/cage	24 hr, 11 dy	Neg Ion
	Cumulative Mortality	PR8 strain of influenza virus; 10/cage	24 hr, 11 dy	Neg Ion
	Cumulative Mortality	PR8 strain of influenza virus; 10/cage	24 hr, 11 dy	Pos Ion
	Cumulative Mortality	PR8 strain of influenza virus; 10/cage	24 hr, 11 dy	Pos Ion

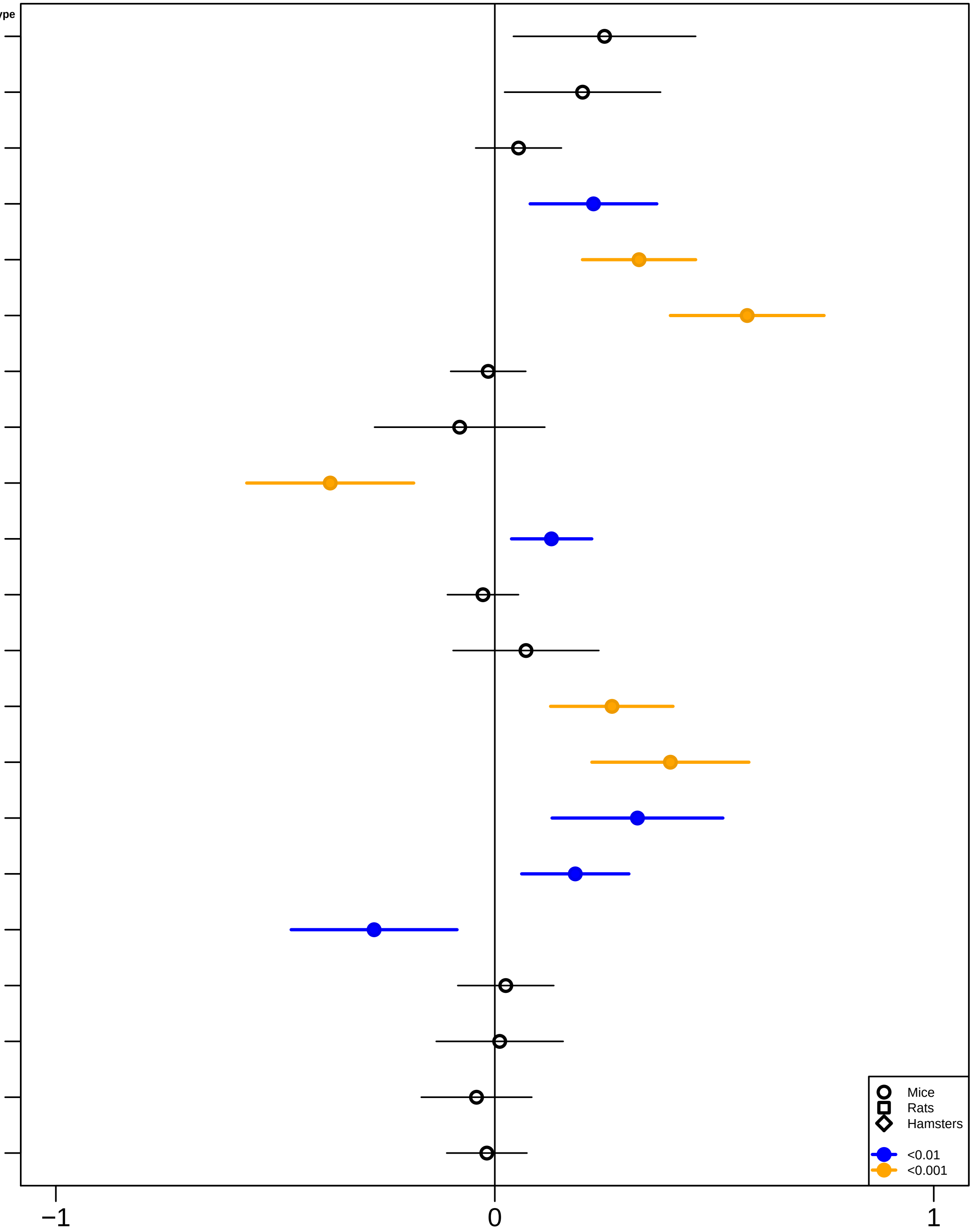

Figure S4. Proportional Differences − Air Ion Respiratory Infection Studies

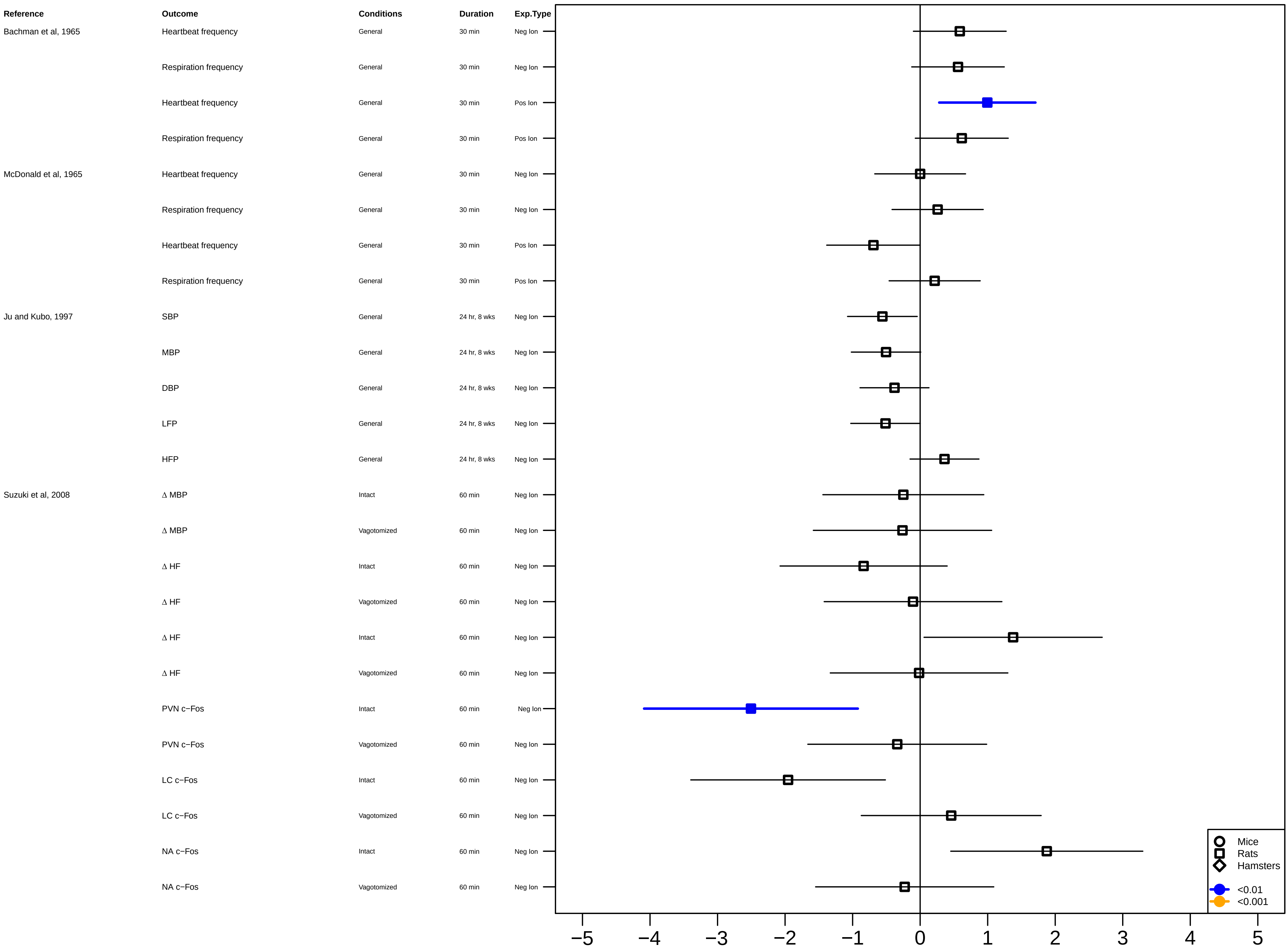

Figure S5. Standardized Mean Differences − Air Ion Cardiovascular Function Studies

Reference	Outcome	Conditions	Duration	Exp.Type
Hinsull et al., 1984	Body weight	F1 females; group exposed	260 dy	Neg Ion
	Body weight	F1 males; group exposed	260 dy	Neg Ion
	Body weight	F2 females; group exposed	260 dy	Neg Ion
	Body weight	F2 males; group exposed	260 dy	Neg Ion
Kellogg et al., 1985a	Glucose (serum)	Females; 25/cage	316 dy	Neg Ion
	Glucose (serum)	Females; 25/cage	316 dy	Neg Ion
	Cholesterol (serum)	Females; 25/cage	316 dy	Neg Ion
	Cholesterol (serum)	Females; 25/cage	316 dy	Neg Ion
	Urea Nitrogen (serum)	Females; 25/cage	316 dy	Neg Ion
	Urea Nitrogen (serum)	Females; 25/cage	316 dy	Neg Ion
	Globulin (serum)	Females; 25/cage	316 dy	Neg Ion
	Globulin (serum)	Females; 25/cage	316 dy	Neg Ion
	Body weight	Females; 25/cage	316 dy	Neg Ion
	Body weight	Females; 25/cage	316 dy	Neg Ion
	Glucose (serum)	Females; 25/cage	316 dy	Pos Ion
	Glucose (serum)	Females; 25/cage	316 dy	Pos Ion
	Cholesterol (serum)	Females; 25/cage	316 dy	Pos Ion
	Cholesterol (serum)	Females; 25/cage	316 dy	Pos Ion
	Urea Nitrogen (serum)	Females; 25/cage	316 dy	Pos Ion
	Urea Nitrogen (serum)	Females; 25/cage	316 dy	Pos Ion
	Globulin (serum)	Females; 25/cage	316 dy	Pos Ion
	Globulin (serum)	Females; 25/cage	316 dy	Pos Ion
	Body weight	Females; 25/cage	316 dy	Pos Ion
	Body weight	Females; 25/cage	316 dy	Pos Ion
Kellogg et al., 1985b	Glucose (serum)	Females; 25/cage	680 dy	Neg Ion
	Glucose (serum)	Females; 25/cage	680 dy	Neg Ion
	Cholesterol (serum)	Females; 25/cage	680 dy	Neg Ion
	Cholesterol (serum)	Females; 25/cage	680 dy	Neg Ion
	Urea Nitrogen (serum)	Females; 25/cage	680 dy	Neg Ion
	Urea Nitrogen (serum)	Females; 25/cage	680 dy	Neg Ion
	Globulin (serum)	Females; 25/cage	260 dy	Neg Ion
	Globulin (serum)	Females; 25/cage	260 dy	Neg Ion
	Glucose (serum)	Females; 25/cage	260 dy	Pos Ion
	Glucose (serum)	Females; 25/cage	260 dy	Pos Ion
	Cholesterol (serum)	Females; 25/cage	260 dy	Pos Ion
	Cholesterol (serum)	Females; 25/cage	260 dy	Pos Ion
	Urea Nitrogen (serum)	Females; 25/cage	260 dy	Pos Ion
	Urea Nitrogen (serum)	Females; 25/cage	260 dy	Pos Ion
	Globulin (serum)	Females; 25/cage	260 dy	Pos Ion
	Globulin (serum)	Females; 25/cage	260 dy	Pos Ion
Hinsull and Head, 1986	Body weight	F0 males; 5/cage	50 wk	Pos Ion
	Body weight	F1 males; 5/cage	50 wk	Pos Ion
Kellog and Yost, 1986	Glucose (serum)	Females; 25/cage	680 dy	Mix
Hinsull et al., 1988	Body weight	Males; 5/cage	95 wk	Neg Ion
	Body weight	Females; 5/cage	95 wk	Neg Ion
Yamamoto et al., 2014	Body weight	5 rm cage w/2 subrm prtns; 2 dams/rm	6 hr/dy, 20 dy	Mix
	Body weight gain	5 rm cage w/2 subrm prtns; 2 dams/rm	6 hr/dy, 20 dy	Mix
	Food consumption	5 rm cage w/2 subrm prtns; 2 dams/rm	6 hr/dy, 20 dy	Mix
	Gravid uterine weight	5 rm cage w/2 subrm prtns; 2 dams/rm	6 hr/dy, 20 dy	Mix
	Corrected body weight	5 rm cage w/2 subrm prtns; 2 dams/rm	6 hr/dy, 20 dy	Mix
	Degree of ossification Sternebrae	5 rm cage w/2 subrm prtns; 2 dams/rm	6 hr/dy, 20 dy	Mix
	Degree of ossification Sacral/caudal vertebrae	5 rm cage w/2 subrm prtns; 2 dams/rm	6 hr/dy, 20 dy	Mix

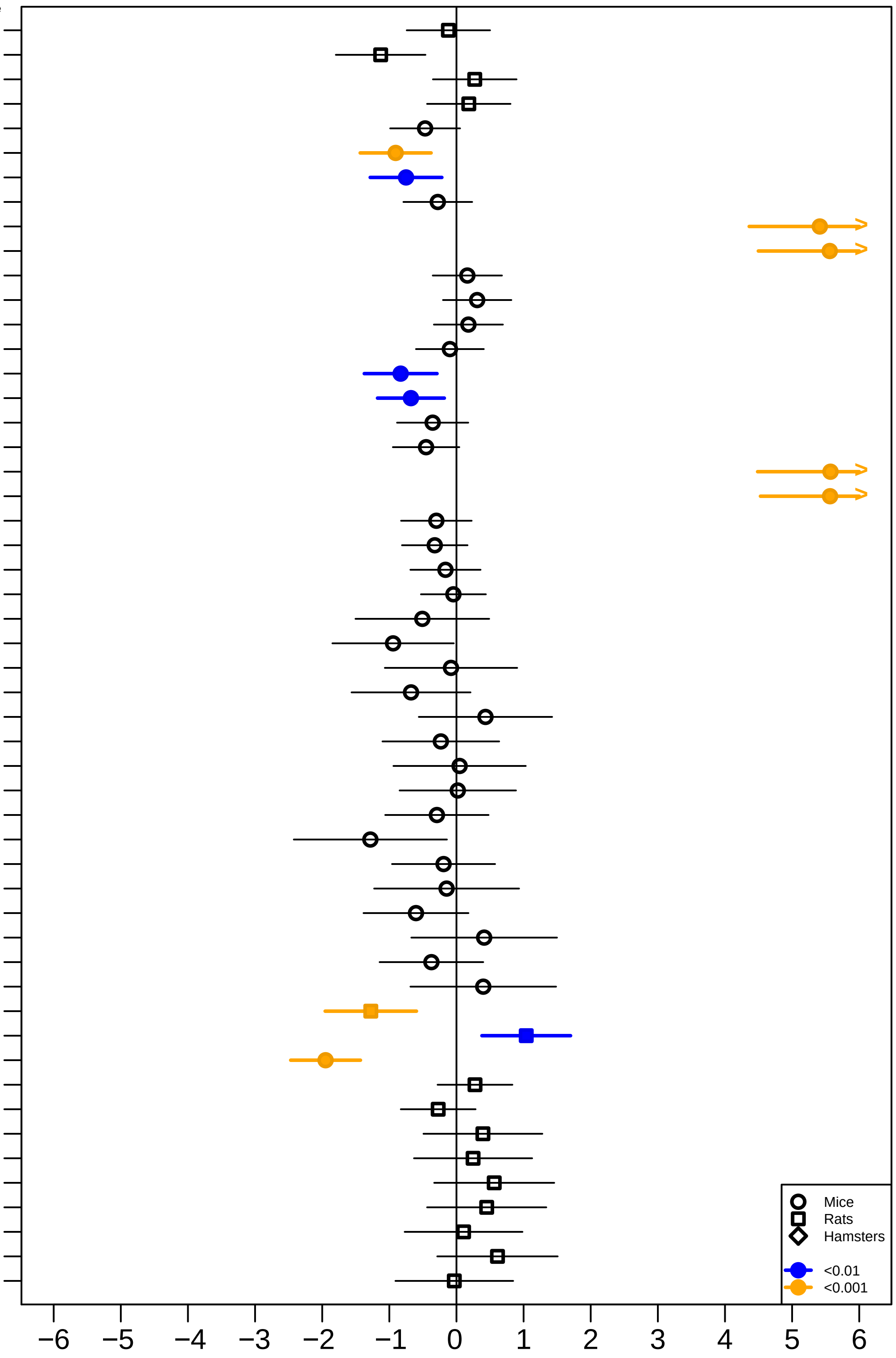

Figure S6. Standardized Mean Differences − Air Ion Reproduction and Growth Studies

Reference	Outcome	Conditions	Duration	Exp.Type
Yamamoto et al., 2015	Final body weight (g)	F1 offspring; female; 2/rm; 5 rm cage w/2 subrm prtns	6 hr/dy, 21 dy	Mix
	Brain (g)	F1 offspring; female; 2/rm; 5 rm cage w/2 subrm prtns	6 hr/dy, 21 dy	Mix
	Relative brain (%g)	F1 offspring; female; 2/rm; 5 rm cage w/2 subrm prtns	6 hr/dy, 21 dy	Mix
	Thymus (mg)	F1 offspring; female; 2/rm; 5 rm cage w/2 subrm prtns	6 hr/dy, 21 dy	Mix
	Relative thymus (%mg)	F1 offspring; female; 2/rm; 5 rm cage w/2 subrm prtns	6 hr/dy, 21 dy	Mix
	Spleen (g	F1 offspring; female; 2/rm; 5 rm cage w/2 subrm prtns	6 hr/dy, 21 dy	Mix
	Relative spleen (%g)	F1 offspring; female; 2/rm; 5 rm cage w/2 subrm prtns	6 hr/dy, 21 dy	Mix
	Uterus (mg)	F1 offspring; female; 2/rm; 5 rm cage w/2 subrm prtns	6 hr/dy, 21 dy	Mix
	Relative uterus (%mg)	F1 offspring; female; 2/rm; 5 rm cage w/2 subrm prtns	6 hr/dy, 21 dy	Mix
	Final body weight (g)	F2 offspring; female; 2/rm; 5 rm cage w/2 subrm prtns	6 hr/dy, 21 dy	Mix
	Brain (g)	F2 offspring; female; 2/rm; 5 rm cage w/2 subrm prtns	6 hr/dy, 21 dy	Mix
	Relative brain (%g)	F2 offspring; female; 2/rm; 5 rm cage w/2 subrm prtns	6 hr/dy, 21 dy	Mix
	Thymus (mg)	F2 offspring; female; 2/rm; 5 rm cage w/2 subrm prtns	6 hr/dy, 21 dy	Mix
	Relative thymus (%mg)	F2 offspring; female; 2/rm; 5 rm cage w/2 subrm prtns	6 hr/dy, 21 dy	Mix
	Spleen (g	F2 offspring; female; 2/rm; 5 rm cage w/2 subrm prtns	6 hr/dy, 21 dy	Mix
	Relative spleen (%g)	F2 offspring; female; 2/rm; 5 rm cage w/2 subrm prtns	6 hr/dy, 21 dy	Mix
	Uterus (mg)	F2 offspring; female; 2/rm; 5 rm cage w/2 subrm prtns	6 hr/dy, 21 dy	Mix
	Relative uterus (%mg)	F2 offspring; female; 2/rm; 5 rm cage w/2 subrm prtns	6 hr/dy, 21 dy	Mix
	Final body weight (g)	F1 offspring; male; 2/rm; 5 rm cage w/2 subrm prtns	6 hr/dy, 21 dy	Mix
	Brain (g)	F1 offspring; male; 2/rm; 5 rm cage w/2 subrm prtns	6 hr/dy, 21 dy	Mix
	Relative brain (%g)	F1 offspring; male; 2/rm; 5 rm cage w/2 subrm prtns	6 hr/dy, 21 dy	Mix
	Thymus (mg)	F1 offspring; male; 2/rm; 5 rm cage w/2 subrm prtns	6 hr/dy, 21 dy	Mix
	Relative thymus (%mg)	F1 offspring; male; 2/rm; 5 rm cage w/2 subrm prtns	6 hr/dy, 21 dy	Mix
	Spleen (g	F1 offspring; male; 2/rm; 5 rm cage w/2 subrm prtns	6 hr/dy, 21 dy	Mix
	Relative spleen (%g)	F1 offspring; male; 2/rm; 5 rm cage w/2 subrm prtns	6 hr/dy, 21 dy	Mix
	Final body weight (g)	F2 offspring; male; 2/rm; 5 rm cage w/2 subrm prtns	6 hr/dy, 21 dy	Mix
	Brain (g)	F2 offspring; male; 2/rm; 5 rm cage w/2 subrm prtns	6 hr/dy, 21 dy	Mix
	Relative brain (%g)	F2 offspring; male; 2/rm; 5 rm cage w/2 subrm prtns	6 hr/dy, 21 dy	Mix
	Thymus (mg)	F2 offspring; male; 2/rm; 5 rm cage w/2 subrm prtns	6 hr/dy, 21 dy	Mix
	Relative thymus (%mg)	F2 offspring; male; 2/rm; 5 rm cage w/2 subrm prtns	6 hr/dy, 21 dy	Mix
	Spleen (g	F2 offspring; male; 2/rm; 5 rm cage w/2 subrm prtns	6 hr/dy, 21 dy	Mix
	Relative spleen (%g)	F2 offspring; male; 2/rm; 5 rm cage w/2 subrm prtns	6 hr/dy, 21 dy	Mix
	Sexual maturation age (day)	F1 offspring; male; 2/rm; 5 rm cage w/2 subrm prtns	6 hr/dy, 21 dy	Mix
	Sexual maturation body weight (g)	F1 offspring; male; 2/rm; 5 rm cage w/2 subrm prtns	6 hr/dy, 21 dy	Mix
	Sexual maturation age (day)	F1 offspring; male; 2/rm; 5 rm cage w/2 subrm prtns	6 hr/dy, 21 dy	Mix
	Sexual maturation body weight (g)	F1 offspring; male; 2/rm; 5 rm cage w/2 subrm prtns	6 hr/dy, 21 dy	Mix
	Days until copulation	F0 parental/F1 offspring; 2/rm; 5 rm cage w/2 subrm prtns	6 hr/dy; 10−12 wk	Mix
	# of estrus stages	F0 parental/F1 offspring; 2/rm; 5 rm cage w/2 subrm prtns	6 hr/dy; 10−12 wk	Mix
	Gestation length (days)	F0 parental/F1 offspring; 2/rm; 5 rm cage w/2 subrm prtns	6 hr/dy; 10−12 wk	Mix
	Birth index (%)	F0 parental/F1 offspring; 2/rm; 5 rm cage w/2 subrm prtns	6 hr/dy; 10−12 wk	Mix
	# implantation sites	F0 parental/F1 offspring; 2/rm; 5 rm cage w/2 subrm prtns	6 hr/dy; 10−12 wk	Mix
	# offspring at birth	F0 parental/F1 offspring; 2/rm; 5 rm cage w/2 subrm prtns	6 hr/dy; 10−12 wk	Mix
	# live offspring at birth	F0 parental/F1 offspring; 2/rm; 5 rm cage w/2 subrm prtns	6 hr/dy; 10−12 wk	Mix
	Live birth index (%)	F0 parental/F1 offspring; 2/rm; 5 rm cage w/2 subrm prtns	6 hr/dy; 10−12 wk	Mix
	Viability index day 4 (%)	F0 parental/F1 offspring; 2/rm; 5 rm cage w/2 subrm prtns	6 hr/dy; 10−12 wk	Mix
	Weaning index day 21 (%)	F0 parental/F1 offspring; 2/rm; 5 rm cage w/2 subrm prtns	6 hr/dy; 10−12 wk	Mix
	Body weight offspring (day 21)	F0 parental/F1 offspring; female; 2/rm; 5 rm cage w/2 subrm prtns	6 hr/dy, 21 dy	Mix
	Body weight gain offspring (day 4−21)	F0 parental/F1 offspring; female; 2/rm; 5 rm cage w/2 subrm prtns	6 hr/dy, 21 dy	Mix
	Body weight offspring (day 21)	F0 parental/F1 offspring; male; 2/rm; 5 rm cage w/2 subrm prtns	6 hr/dy, 21 dy	Mix
	Body weight gain offspring (day 4−21)	F0 parental/F1 offspring; male; 2/rm; 5 rm cage w/2 subrm prtns	6 hr/dy, 21 dy	Mix
	Days until copulation	F1 parental/F2 offspring; 2/rm; 5 rm cage w/2 subrm prtns	6 hr/dy; 10−12 wk	Mix
	# of estrus stages	F1 parental/F2 offspring; 2/rm; 5 rm cage w/2 subrm prtns	6 hr/dy; 10−12 wk	Mix
	Gestation length (days)	F1 parental/F2 offspring; 2/rm; 5 rm cage w/2 subrm prtns	6 hr/dy; 10−12 wk	Mix
	Birth index (%)	F1 parental/F2 offspring; 2/rm; 5 rm cage w/2 subrm prtns	6 hr/dy; 10−12 wk	Mix
	# implantation sites	F1 parental/F2 offspring; 2/rm; 5 rm cage w/2 subrm prtns	6 hr/dy; 10−12 wk	Mix
	# offspring at birth	F1 parental/F2 offspring; 2/rm; 5 rm cage w/2 subrm prtns	6 hr/dy; 10−12 wk	Mix
	# live offspring at birth	F1 parental/F2 offspring; 2/rm; 5 rm cage w/2 subrm prtns	6 hr/dy; 10−12 wk	Mix
	Live birth index (%)	F1 parental/F2 offspring; 2/rm; 5 rm cage w/2 subrm prtns	6 hr/dy; 10−12 wk	Mix
	Viability index day 4 (%)	F1 parental/F2 offspring; 2/rm; 5 rm cage w/2 subrm prtns	6 hr/dy; 10−12 wk	Mix
	Weaning index day 21 (%)	F1 parental/F2 offspring; 2/rm; 5 rm cage w/2 subrm prtns	6 hr/dy; 10−12 wk	Mix
	Body weight offspring (day 21)	F1 parental/F2 offspring; females; 2/rm; 5 rm cage w/2 subrm prtns	6 hr/dy, 21 dy	Mix
	Body weight gain offspring (day 4−21)	F1 parental/F2 offspring; females; 2/rm; 5 rm cage w/2 subrm prtns	6 hr/dy, 21 dy	Mix
	Body weight offspring (day 21)	F1 parental/F2 offspring; males; 2/rm; 5 rm cage w/2 subrm prtns	6 hr/dy, 21 dy	Mix
	Body weight gain offspring (day 4−21)	F1 parental/F2 offspring; males; 2/rm; 5 rm cage w/2 subrm prtns	6 hr/dy, 21 dy	Mix

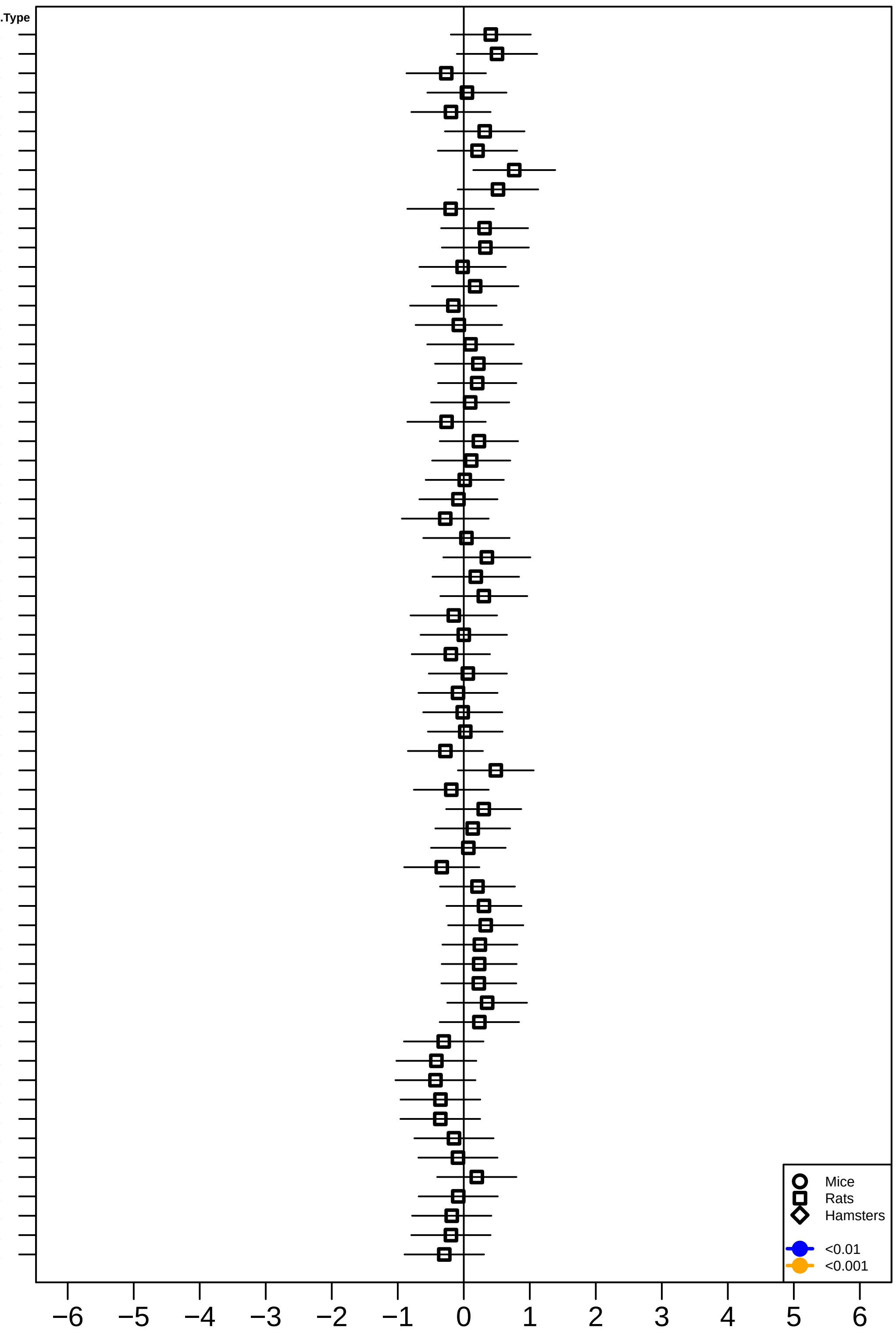

Figure S6 (Cont.)

Reference	Outcome	Conditions	Duration	Exp.Type
Yamamoto et al., 2015	Mean estrous cycle length (days)	F0 parental; 2/rm; 5 rm cage w/2 subrm prtns	6 hr/dy; 10−12 wk	Mix
(Cont.)	Mean estrous cycle length (days)	F1 parental; 2/rm; 5 rm cage w/2 subrm prtns	6 hr/dy; 10−12 wk	Mix
	Sperm motility (%)	F0 parental; 2/rm; 5 rm cage w/2 subrm prtns	6 hr/dy; 10−12 wk	Mix
	Sperm motility (%)	F1 parental; 2/rm; 5 rm cage w/2 subrm prtns	6 hr/dy; 10−12 wk	Mix
	Homogenization−resistant spermatids (10^6/g testis)	F0 parental; 2/rm; 5 rm cage w/2 subrm prtns	6 hr/dy; 10−12 wk	Mix
	Homogenization−resistant spermatids (10^6/g testis)	F1 parental; 2/rm; 5 rm cage w/2 subrm prtns	6 hr/dy; 10−12 wk	Mix
	Sperm count (10^6/g cauda epididymis)	F0 parental; 2/rm; 5 rm cage w/2 subrm prtns	6 hr/dy; 10−12 wk	Mix
	Sperm count (10^6/g cauda epididymis)	F1 parental; 2/rm; 5 rm cage w/2 subrm prtns	6 hr/dy; 10−12 wk	Mix
	Abnormal sperm (%)	F0 parental; 2/rm; 5 rm cage w/2 subrm prtns	6 hr/dy; 10−12 wk	Mix
	Abnormal sperm (%)	F1 parental; 2/rm; 5 rm cage w/2 subrm prtns	6 hr/dy; 10−12 wk	Mix
	Tailless sperm (%)	F0 parental; 2/rm; 5 rm cage w/2 subrm prtns	6 hr/dy; 10−12 wk	Mix
	Tailless sperm (%)	F1 parental; 2/rm; 5 rm cage w/2 subrm prtns	6 hr/dy; 10−12 wk	Mix
	Small ovarian follicles	F1 parental; female; 2/rm; 5 rm cage w/2 subrm prtns	6 hr/dy; 10−12 wk	Mix
	Medium ovarian follicles	F1 parental; female; 2/rm; 5 rm cage w/2 subrm prtns	6 hr/dy; 10−12 wk	Mix
	Large ovarian follicles	F1 parental; female; 2/rm; 5 rm cage w/2 subrm prtns	6 hr/dy; 10−12 wk	Mix
	Total ovarian follicles	F1 parental; female; 2/rm; 5 rm cage w/2 subrm prtns	6 hr/dy; 10−12 wk	Mix
	Final body weight (g)	F0 parental; male; 2/rm; 5 rm cage w/2 subrm prtns	6 hr/dy; 10−12 wk	Mix
	Brain (g)	F0 parental; male; 2/rm; 5 rm cage w/2 subrm prtns	6 hr/dy; 10−12 wk	Mix
	Relative brain (%g)	F0 parental; male; 2/rm; 5 rm cage w/2 subrm prtns	6 hr/dy; 10−12 wk	Mix
	Pituitary (mg)	F0 parental; male; 2/rm; 5 rm cage w/2 subrm prtns	6 hr/dy; 10−12 wk	Mix
	Relative pituitary (%mg)	F0 parental; male; 2/rm; 5 rm cage w/2 subrm prtns	6 hr/dy; 10−12 wk	Mix
	Thyroids (mg)	F0 parental; male; 2/rm; 5 rm cage w/2 subrm prtns	6 hr/dy; 10−12 wk	Mix
	Relative thyroids (%mg)	F0 parental; male; 2/rm; 5 rm cage w/2 subrm prtns	6 hr/dy; 10−12 wk	Mix
	Thymus (mg)	F0 parental; male; 2/rm; 5 rm cage w/2 subrm prtns	6 hr/dy; 10−12 wk	Mix
	Relative thymus (%mg)	F0 parental; male; 2/rm; 5 rm cage w/2 subrm prtns	6 hr/dy; 10−12 wk	Mix
	Lungs (g)	F0 parental; male; 2/rm; 5 rm cage w/2 subrm prtns	6 hr/dy; 10−12 wk	Mix
	Relative lungs (%g)	F0 parental; male; 2/rm; 5 rm cage w/2 subrm prtns	6 hr/dy; 10−12 wk	Mix
	Heart (g)	F0 parental; male; 2/rm; 5 rm cage w/2 subrm prtns	6 hr/dy; 10−12 wk	Mix
	Relative heart (%g)	F0 parental; male; 2/rm; 5 rm cage w/2 subrm prtns	6 hr/dy; 10−12 wk	Mix
	Liver (g)	F0 parental; male; 2/rm; 5 rm cage w/2 subrm prtns	6 hr/dy; 10−12 wk	Mix
	Relative liver (%g)	F0 parental; male; 2/rm; 5 rm cage w/2 subrm prtns	6 hr/dy; 10−12 wk	Mix
	Spleen (g)	F0 parental; male; 2/rm; 5 rm cage w/2 subrm prtns	6 hr/dy; 10−12 wk	Mix
	Relative spleen (%g)	F0 parental; male; 2/rm; 5 rm cage w/2 subrm prtns	6 hr/dy; 10−12 wk	Mix
	Kidneys (g)	F0 parental; male; 2/rm; 5 rm cage w/2 subrm prtns	6 hr/dy; 10−12 wk	Mix
	Relative kidneys (%g)	F0 parental; male; 2/rm; 5 rm cage w/2 subrm prtns	6 hr/dy; 10−12 wk	Mix
	Adrenals (mg)	F0 parental; male; 2/rm; 5 rm cage w/2 subrm prtns	6 hr/dy; 10−12 wk	Mix
	Relative adrenals (%mg)	F0 parental; male; 2/rm; 5 rm cage w/2 subrm prtns	6 hr/dy; 10−12 wk	Mix
	Testes (g)	F0 parental; male; 2/rm; 5 rm cage w/2 subrm prtns	6 hr/dy; 10−12 wk	Mix
	Relative testes (%g)	F0 parental; male; 2/rm; 5 rm cage w/2 subrm prtns	6 hr/dy; 10−12 wk	Mix
	Prostate (g)	F0 parental; male; 2/rm; 5 rm cage w/2 subrm prtns	6 hr/dy; 10−12 wk	Mix
	Relative prostate (%g)	F0 parental; male; 2/rm; 5 rm cage w/2 subrm prtns	6 hr/dy; 10−12 wk	Mix
	Seminal vesicles (g)	F0 parental; male; 2/rm; 5 rm cage w/2 subrm prtns	6 hr/dy; 10−12 wk	Mix
	Relative seminal vesicles (%g)	F0 parental; male; 2/rm; 5 rm cage w/2 subrm prtns	6 hr/dy; 10−12 wk	Mix
	Epididymides (g)	F0 parental; male; 2/rm; 5 rm cage w/2 subrm prtns	6 hr/dy; 10−12 wk	Mix
	Relative epididymides (%g)	F0 parental; male; 2/rm; 5 rm cage w/2 subrm prtns	6 hr/dy; 10−12 wk	Mix
	Final body weight (g)	F1 parental; male; 2/rm; 5 rm cage w/2 subrm prtns	6 hr/dy; 10−12 wk	Mix
	Brain (g)	F1 parental; male; 2/rm; 5 rm cage w/2 subrm prtns	6 hr/dy; 10−12 wk	Mix
	Relative brain (%g)	F1 parental; male; 2/rm; 5 rm cage w/2 subrm prtns	6 hr/dy; 10−12 wk	Mix
	Pituitary (mg)	F1 parental; male; 2/rm; 5 rm cage w/2 subrm prtns	6 hr/dy; 10−12 wk	Mix
	Relative pituitary (%mg)	F1 parental; male; 2/rm; 5 rm cage w/2 subrm prtns	6 hr/dy; 10−12 wk	Mix
	Thyroids (mg)	F1 parental; male; 2/rm; 5 rm cage w/2 subrm prtns	6 hr/dy; 10−12 wk	Mix
	Relative thyroids (%mg)	F1 parental; male; 2/rm; 5 rm cage w/2 subrm prtns	6 hr/dy; 10−12 wk	Mix
	Thymus (mg)	F1 parental; male; 2/rm; 5 rm cage w/2 subrm prtns	6 hr/dy; 10−12 wk	Mix
	Relative thymus (%mg)	F1 parental; male; 2/rm; 5 rm cage w/2 subrm prtns	6 hr/dy; 10−12 wk	Mix
	Lungs (g)	F1 parental; male; 2/rm; 5 rm cage w/2 subrm prtns	6 hr/dy; 10−12 wk	Mix
	Relative lungs (%g)	F1 parental; male; 2/rm; 5 rm cage w/2 subrm prtns	6 hr/dy; 10−12 wk	Mix
	Heart (g)	F1 parental; male; 2/rm; 5 rm cage w/2 subrm prtns	6 hr/dy; 10−12 wk	Mix

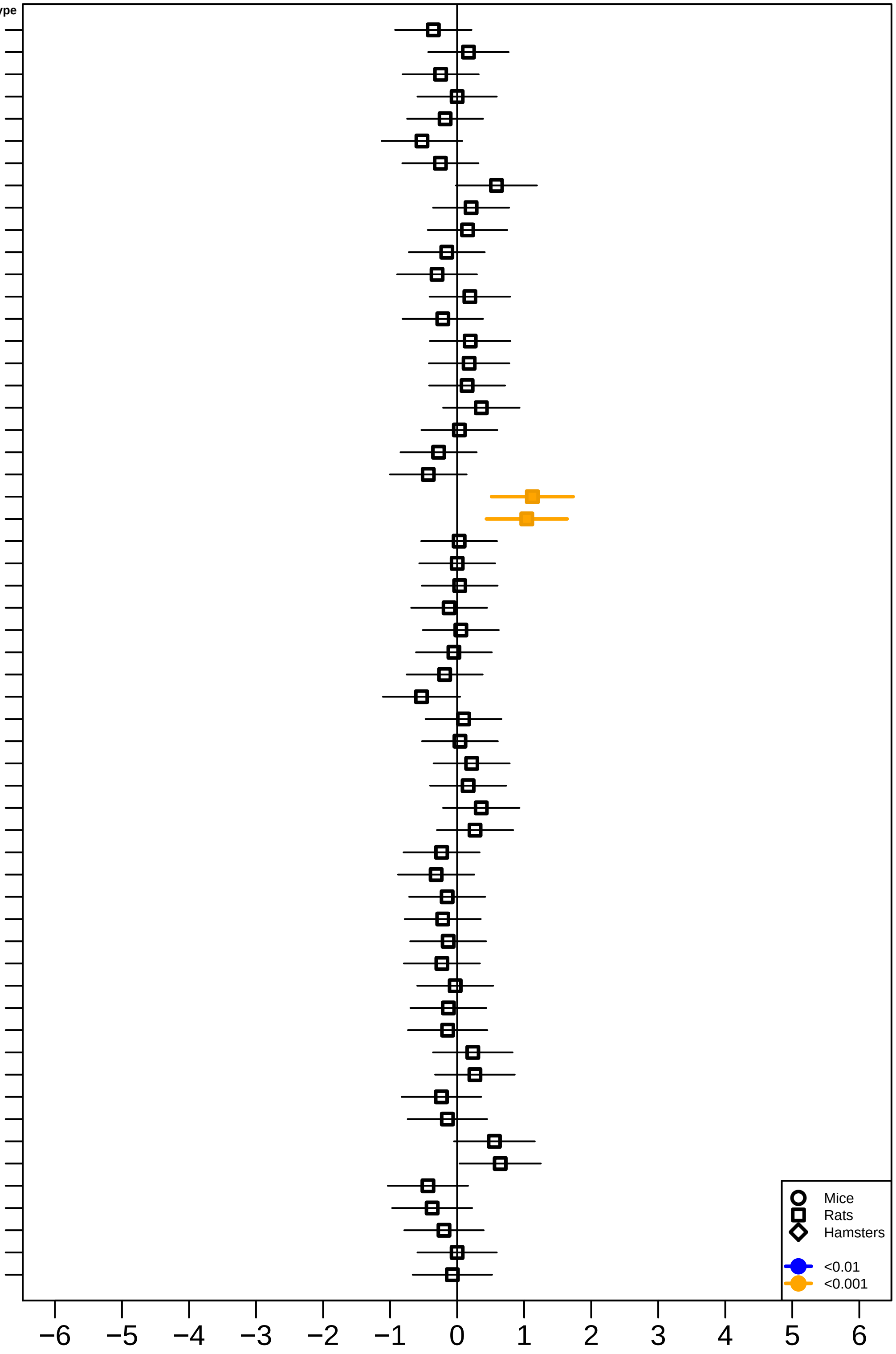

Figure S6 (Cont.)

Reference	Outcome	Conditions	Duration	Exp.Type
Yamamoto et al., 2015	Relative heart (%g)	F1 parental; male; 2/rm; 5 rm cage w/2 subrm prtns	6 hr/dy; 10−12 wk	Mix
(Cont.)	Liver (g)	F1 parental; male; 2/rm; 5 rm cage w/2 subrm prtns	6 hr/dy; 10−12 wk	Mix
	Relative liver (%g)	F1 parental; male; 2/rm; 5 rm cage w/2 subrm prtns	6 hr/dy; 10−12 wk	Mix
	Spleen (g)	F1 parental; male; 2/rm; 5 rm cage w/2 subrm prtns	6 hr/dy; 10−12 wk	Mix
	Relative spleen (%g)	F1 parental; male; 2/rm; 5 rm cage w/2 subrm prtns	6 hr/dy; 10−12 wk	Mix
	Kidneys (g)	F1 parental; male; 2/rm; 5 rm cage w/2 subrm prtns	6 hr/dy; 10−12 wk	Mix
	Relative kidneys (%g)	F1 parental; male; 2/rm; 5 rm cage w/2 subrm prtns	6 hr/dy; 10−12 wk	Mix
	Adrenals (mg)	F1 parental; male; 2/rm; 5 rm cage w/2 subrm prtns	6 hr/dy; 10−12 wk	Mix
	Relative adrenals (%mg)	F1 parental; male; 2/rm; 5 rm cage w/2 subrm prtns	6 hr/dy; 10−12 wk	Mix
	Testes (g)	F1 parental; male; 2/rm; 5 rm cage w/2 subrm prtns	6 hr/dy; 10−12 wk	Mix
	Relative testes (%g)	F1 parental; male; 2/rm; 5 rm cage w/2 subrm prtns	6 hr/dy; 10−12 wk	Mix
	Prostate (g)	F1 parental; male; 2/rm; 5 rm cage w/2 subrm prtns	6 hr/dy; 10−12 wk	Mix
	Relative prostate (%g)	F1 parental; male; 2/rm; 5 rm cage w/2 subrm prtns	6 hr/dy; 10−12 wk	Mix
	Seminal vesicles (g)	F1 parental; male; 2/rm; 5 rm cage w/2 subrm prtns	6 hr/dy; 10−12 wk	Mix
	Relative seminal vesicles (%g)	F1 parental; male; 2/rm; 5 rm cage w/2 subrm prtns	6 hr/dy; 10−12 wk	Mix
	Epididymides (g)	F1 parental; male; 2/rm; 5 rm cage w/2 subrm prtns	6 hr/dy; 10−12 wk	Mix
	Relative epididymides (%g)	F1 parental; male; 2/rm; 5 rm cage w/2 subrm prtns	6 hr/dy; 10−12 wk	Mix
	Final body weight (g)	F0 parental; female; 2/rm; 5 rm cage w/2 subrm prtns	6 hr/dy; 10−12 wk	Mix
	Brain (g)	F0 parental; female; 2/rm; 5 rm cage w/2 subrm prtns	6 hr/dy; 10−12 wk	Mix
	Relative brain (%g)	F0 parental; female; 2/rm; 5 rm cage w/2 subrm prtns	6 hr/dy; 10−12 wk	Mix
	Pituitary (mg)	F0 parental; female; 2/rm; 5 rm cage w/2 subrm prtns	6 hr/dy; 10−12 wk	Mix
	Relative pituitary (%mg)	F0 parental; female; 2/rm; 5 rm cage w/2 subrm prtns	6 hr/dy; 10−12 wk	Mix
	Thyroids (mg)	F0 parental; female; 2/rm; 5 rm cage w/2 subrm prtns	6 hr/dy; 10−12 wk	Mix
	Relative thyroids (%mg)	F0 parental; female; 2/rm; 5 rm cage w/2 subrm prtns	6 hr/dy; 10−12 wk	Mix
	Thymus (mg)	F0 parental; female; 2/rm; 5 rm cage w/2 subrm prtns	6 hr/dy; 10−12 wk	Mix
	Relative thymus (%mg)	F0 parental; female; 2/rm; 5 rm cage w/2 subrm prtns	6 hr/dy; 10−12 wk	Mix
	Lungs (g)	F0 parental; female; 2/rm; 5 rm cage w/2 subrm prtns	6 hr/dy; 10−12 wk	Mix
	Relative lungs (%g)	F0 parental; female; 2/rm; 5 rm cage w/2 subrm prtns	6 hr/dy; 10−12 wk	Mix
	Heart (g)	F0 parental; female; 2/rm; 5 rm cage w/2 subrm prtns	6 hr/dy; 10−12 wk	Mix
	Relative heart (%g)	F0 parental; female; 2/rm; 5 rm cage w/2 subrm prtns	6 hr/dy; 10−12 wk	Mix
	Liver (g)	F0 parental; female; 2/rm; 5 rm cage w/2 subrm prtns	6 hr/dy; 10−12 wk	Mix
	Relative liver (%g)	F0 parental; female; 2/rm; 5 rm cage w/2 subrm prtns	6 hr/dy; 10−12 wk	Mix
	Spleen (g)	F0 parental; female; 2/rm; 5 rm cage w/2 subrm prtns	6 hr/dy; 10−12 wk	Mix
	Relative spleen (%g)	F0 parental; female; 2/rm; 5 rm cage w/2 subrm prtns	6 hr/dy; 10−12 wk	Mix
	Kidneys (g)	F0 parental; female; 2/rm; 5 rm cage w/2 subrm prtns	6 hr/dy; 10−12 wk	Mix
	Relative kidneys (%g)	F0 parental; female; 2/rm; 5 rm cage w/2 subrm prtns	6 hr/dy; 10−12 wk	Mix
	Adrenals (mg)	F0 parental; female; 2/rm; 5 rm cage w/2 subrm prtns	6 hr/dy; 10−12 wk	Mix
	Relative adrenals (%mg)	F0 parental; female; 2/rm; 5 rm cage w/2 subrm prtns	6 hr/dy; 10−12 wk	Mix
	Ovaries (mg)	F0 parental; female; 2/rm; 5 rm cage w/2 subrm prtns	6 hr/dy; 10−12 wk	Mix
	Relative ovaries (%mg)	F0 parental; female; 2/rm; 5 rm cage w/2 subrm prtns	6 hr/dy; 10−12 wk	Mix
	Uterus (mg)	F0 parental; female; 2/rm; 5 rm cage w/2 subrm prtns	6 hr/dy; 10−12 wk	Mix
	Relative uterus (%mg)	F0 parental; female; 2/rm; 5 rm cage w/2 subrm prtns	6 hr/dy; 10−12 wk	Mix
	Final body weight (g)	F1 parental; female; 2/rm; 5 rm cage w/2 subrm prtns	6 hr/dy; 10−12 wk	Mix
	Brain (g)	F1 parental; female; 2/rm; 5 rm cage w/2 subrm prtns	6 hr/dy; 10−12 wk	Mix
	Relative brain (%g)	F1 parental; female; 2/rm; 5 rm cage w/2 subrm prtns	6 hr/dy; 10−12 wk	Mix
	Pituitary (mg)	F1 parental; female; 2/rm; 5 rm cage w/2 subrm prtns	6 hr/dy; 10−12 wk	Mix
	Relative pituitary (%mg)	F1 parental; female; 2/rm; 5 rm cage w/2 subrm prtns	6 hr/dy; 10−12 wk	Mix
	Thyroids (mg)	F1 parental; female; 2/rm; 5 rm cage w/2 subrm prtns	6 hr/dy; 10−12 wk	Mix
	Relative thyroids (%mg)	F1 parental; female; 2/rm; 5 rm cage w/2 subrm prtns	6 hr/dy; 10−12 wk	Mix
	Thymus (mg)	F1 parental; female; 2/rm; 5 rm cage w/2 subrm prtns	6 hr/dy; 10−12 wk	Mix
	Relative thymus (%mg)	F1 parental; female; 2/rm; 5 rm cage w/2 subrm prtns	6 hr/dy; 10−12 wk	Mix
	Lungs (g)	F1 parental; female; 2/rm; 5 rm cage w/2 subrm prtns	6 hr/dy; 10−12 wk	Mix
	Relative lungs (%g)	F1 parental; female; 2/rm; 5 rm cage w/2 subrm prtns	6 hr/dy; 10−12 wk	Mix
	Heart (g)	F1 parental; female; 2/rm; 5 rm cage w/2 subrm prtns	6 hr/dy; 10−12 wk	Mix
	Relative heart (%g)	F1 parental; female; 2/rm; 5 rm cage w/2 subrm prtns	6 hr/dy; 10−12 wk	Mix
	Liver (g)	F1 parental; female; 2/rm; 5 rm cage w/2 subrm prtns	6 hr/dy; 10−12 wk	Mix
	Relative liver (%g)	F1 parental; female; 2/rm; 5 rm cage w/2 subrm prtns	6 hr/dy; 10−12 wk	Mix

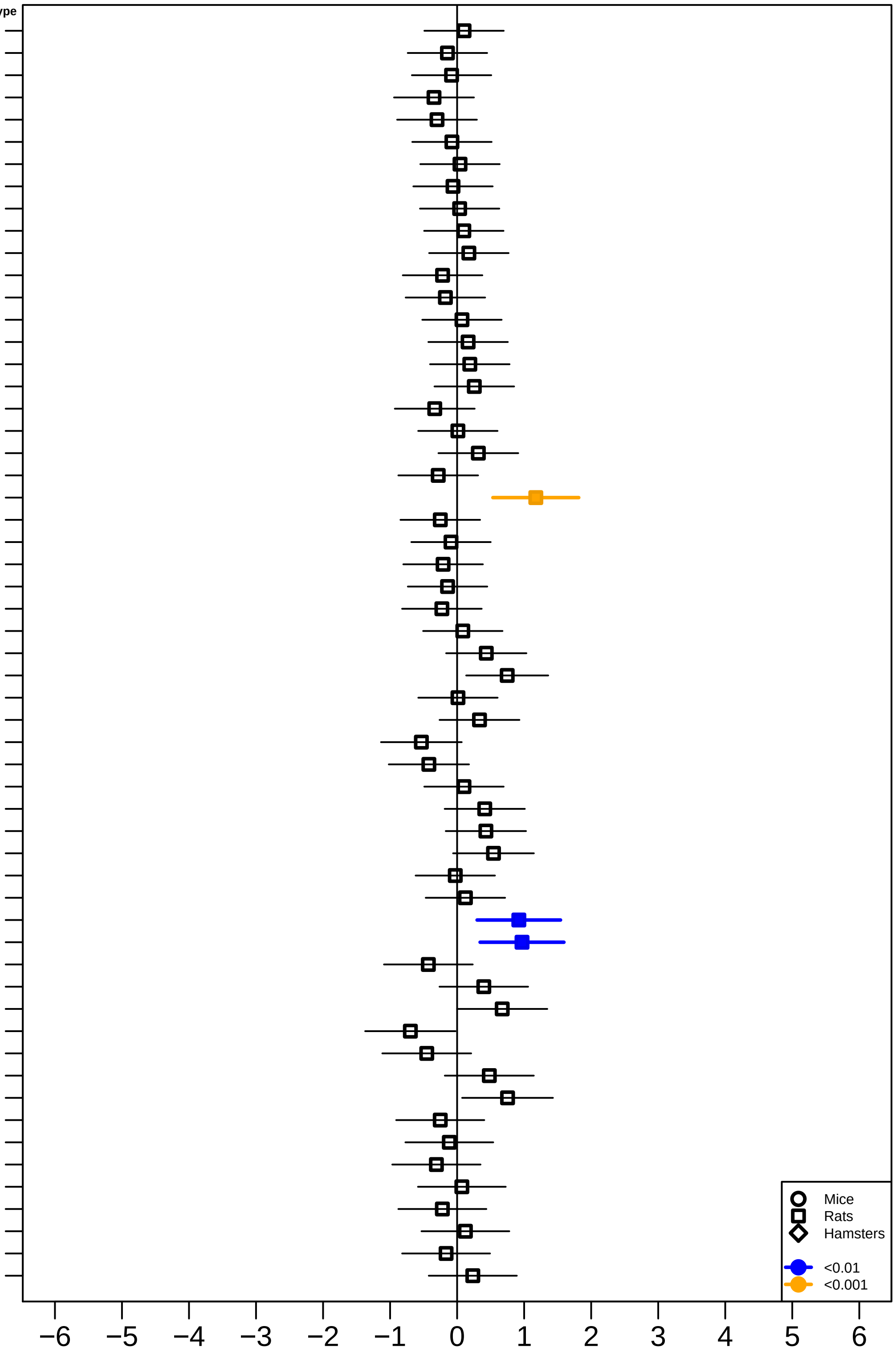

Figure S6 (Cont.)

Reference	Outcome	Conditions	Duration	Exp.Type
Yamamoto et al., 2015	Spleen (g)	F1 parental; female; 2/rm; 5 rm cage w/2 subrm prtns	6 hr/dy; 10−12 wk	Mix
(Cont.)	Relative spleen (%g)	F1 parental; female; 2/rm; 5 rm cage w/2 subrm prtns	6 hr/dy; 10−12 wk	Mix
	Kidneys (g)	F1 parental; female; 2/rm; 5 rm cage w/2 subrm prtns	6 hr/dy; 10−12 wk	Mix
	Relative kidneys (%g)	F1 parental; female; 2/rm; 5 rm cage w/2 subrm prtns	6 hr/dy; 10−12 wk	Mix
	Adrenals (mg)	F1 parental; female; 2/rm; 5 rm cage w/2 subrm prtns	6 hr/dy; 10−12 wk	Mix
	Relative adrenals (%mg)	F1 parental; female; 2/rm; 5 rm cage w/2 subrm prtns	6 hr/dy; 10−12 wk	Mix
	Ovaries (mg)	F1 parental; female; 2/rm; 5 rm cage w/2 subrm prtns	6 hr/dy; 10−12 wk	Mix
	Relative ovaries (%mg)	F1 parental; female; 2/rm; 5 rm cage w/2 subrm prtns	6 hr/dy; 10−12 wk	Mix
	Uterus (mg)	F1 parental; female; 2/rm; 5 rm cage w/2 subrm prtns	6 hr/dy; 10−12 wk	Mix
	Relative uterus (%mg)	F1 parental; female; 2/rm; 5 rm cage w/2 subrm prtns	6 hr/dy; 10−12 wk	Mix
	Body weight (g) pre−mating day 0/21	F0 parental; male; 2/rm; 5 rm cage w/2 subrm prtns	6 hr/dy; 10−12 wk	Mix
	Body weight (g) pre−mating day 70/91	F0 parental; male; 2/rm; 5 rm cage w/2 subrm prtns	6 hr/dy; 10−12 wk	Mix
	Body weight gain (g) pre−mating day 0−7/21−28	F0 parental; male; 2/rm; 5 rm cage w/2 subrm prtns	6 hr/dy; 10−12 wk	Mix
	Body weight gain (g) pre−mating day 0−70/21−91	F0 parental; male; 2/rm; 5 rm cage w/2 subrm prtns	6 hr/dy; 10−12 wk	Mix
	Food consumption pre−mating day 7/28	F0 parental; male; 2/rm; 5 rm cage w/2 subrm prtns	6 hr/dy; 10−12 wk	Mix
	Food consumption pre−mating day 70/91	F0 parental; male; 2/rm; 5 rm cage w/2 subrm prtns	6 hr/dy; 10−12 wk	Mix
	Body weight (g) pre−mating day 0/21	F1 parental; male; 2/rm; 5 rm cage w/2 subrm prtns	6 hr/dy; 10−12 wk	Mix
	Body weight (g) pre−mating day 70/91	F1 parental; male; 2/rm; 5 rm cage w/2 subrm prtns	6 hr/dy; 10−12 wk	Mix
	Body weight gain (g) pre−mating day 0−7/21−28	F1 parental; male; 2/rm; 5 rm cage w/2 subrm prtns	6 hr/dy; 10−12 wk	Mix
	Body weight gain (g) pre−mating day 0−70/21−91	F1 parental; male; 2/rm; 5 rm cage w/2 subrm prtns	6 hr/dy; 10−12 wk	Mix
	Food consumption pre−mating day 7/28	F1 parental; male; 2/rm; 5 rm cage w/2 subrm prtns	6 hr/dy; 10−12 wk	Mix
	Food consumption pre−mating day 70/91	F1 parental; male; 2/rm; 5 rm cage w/2 subrm prtns	6 hr/dy; 10−12 wk	Mix
	Body weight (g) pre−mating day 0/21	F0 parental; female; 2/rm; 5 rm cage w/2 subrm prtns	6 hr/dy; 10−12 wk	Mix
	Body weight (g) pre−mating day 70/91	F0 parental; female; 2/rm; 5 rm cage w/2 subrm prtns	6 hr/dy; 10−12 wk	Mix
	Gestation day 0	F0 parental; female; 2/rm; 5 rm cage w/2 subrm prtns	6 hr/dy; 10−12 wk	Mix
	Gestation day 20	F0 parental; female; 2/rm; 5 rm cage w/2 subrm prtns	6 hr/dy; 10−12 wk	Mix
	Lactation day 0	F0 parental; female; 2/rm; 5 rm cage w/2 subrm prtns	6 hr/dy; 10−12 wk	Mix
	Lactation day 14	F0 parental; female; 2/rm; 5 rm cage w/2 subrm prtns	6 hr/dy; 10−12 wk	Mix
	Lactation day 21	F0 parental; female; 2/rm; 5 rm cage w/2 subrm prtns	6 hr/dy; 10−12 wk	Mix
	Body weight gain (g) pre−mating day 0−7/21−28	F0 parental; female; 2/rm; 5 rm cage w/2 subrm prtns	6 hr/dy; 10−12 wk	Mix
	Body weight gain (g) pre−mating day 0−70/21−91	F0 parental; female; 2/rm; 5 rm cage w/2 subrm prtns	6 hr/dy; 10−12 wk	Mix
	Body weight gain (g) gestation day 0−20	F0 parental; female; 2/rm; 5 rm cage w/2 subrm prtns	6 hr/dy; 10−12 wk	Mix
	Body weight gain (g) lactation day 0−14	F0 parental; female; 2/rm; 5 rm cage w/2 subrm prtns	6 hr/dy; 10−12 wk	Mix
	Body weight gain (g) lactation day 0−21	F0 parental; female; 2/rm; 5 rm cage w/2 subrm prtns	6 hr/dy; 10−12 wk	Mix
	Food consumption pre−mating day 7/28	F0 parental; female; 2/rm; 5 rm cage w/2 subrm prtns	6 hr/dy; 10−12 wk	Mix
	Food consumption pre−mating day 70/91	F0 parental; female; 2/rm; 5 rm cage w/2 subrm prtns	6 hr/dy; 10−12 wk	Mix
	Food consumption gestation day 20	F0 parental; female; 2/rm; 5 rm cage w/2 subrm prtns	6 hr/dy; 10−12 wk	Mix
	Food consumption lactation day 14	F0 parental; female; 2/rm; 5 rm cage w/2 subrm prtns	6 hr/dy; 10−12 wk	Mix
	Food consumption lactation day 21	F0 parental; female; 2/rm; 5 rm cage w/2 subrm prtns	6 hr/dy; 10−12 wk	Mix
	Body weight (g) pre−mating day 0/21	F1 parental; female; 2/rm; 5 rm cage w/2 subrm prtns	6 hr/dy; 10−12 wk	Mix
	Body weight (g) pre−mating day 70/91	F1 parental; female; 2/rm; 5 rm cage w/2 subrm prtns	6 hr/dy; 10−12 wk	Mix
	Gestation day 0	F1 parental; female; 2/rm; 5 rm cage w/2 subrm prtns	6 hr/dy; 10−12 wk	Mix
	Gestation day 20	F1 parental; female; 2/rm; 5 rm cage w/2 subrm prtns	6 hr/dy; 10−12 wk	Mix
	Lactation day 0	F1 parental; female; 2/rm; 5 rm cage w/2 subrm prtns	6 hr/dy; 10−12 wk	Mix
	Lactation day 14	F1 parental; female; 2/rm; 5 rm cage w/2 subrm prtns	6 hr/dy; 10−12 wk	Mix
	Lactation day 21	F1 parental; female; 2/rm; 5 rm cage w/2 subrm prtns	6 hr/dy; 10−12 wk	Mix
	Body weight gain (g) pre−mating day 0−7/21−28	F1 parental; female; 2/rm; 5 rm cage w/2 subrm prtns	6 hr/dy; 10−12 wk	Mix
	Body weight gain (g) pre−mating day 0−70/21−91	F1 parental; female; 2/rm; 5 rm cage w/2 subrm prtns	6 hr/dy; 10−12 wk	Mix
	Body weight gain (g) gestation day 0−20	F1 parental; female; 2/rm; 5 rm cage w/2 subrm prtns	6 hr/dy; 10−12 wk	Mix
	Body weight gain (g) lactation day 0−14	F1 parental; female; 2/rm; 5 rm cage w/2 subrm prtns	6 hr/dy; 10−12 wk	Mix
	Body weight gain (g) lactation day 0−21	F1 parental; female; 2/rm; 5 rm cage w/2 subrm prtns	6 hr/dy; 10−12 wk	Mix
	Food consumption pre−mating day 7/28	F1 parental; female; 2/rm; 5 rm cage w/2 subrm prtns	6 hr/dy; 10−12 wk	Mix
	Food consumption pre−mating day 70/91	F1 parental; female; 2/rm; 5 rm cage w/2 subrm prtns	6 hr/dy; 10−12 wk	Mix
	Food consumption gestation day 20	F1 parental; female; 2/rm; 5 rm cage w/2 subrm prtns	6 hr/dy; 10−12 wk	Mix
	Food consumption lactation day 14	F1 parental; female; 2/rm; 5 rm cage w/2 subrm prtns	6 hr/dy; 10−12 wk	Mix
	Food consumption lactation day 21	F1 parental; female; 2/rm; 5 rm cage w/2 subrm prtns	6 hr/dy; 10−12 wk	Mix

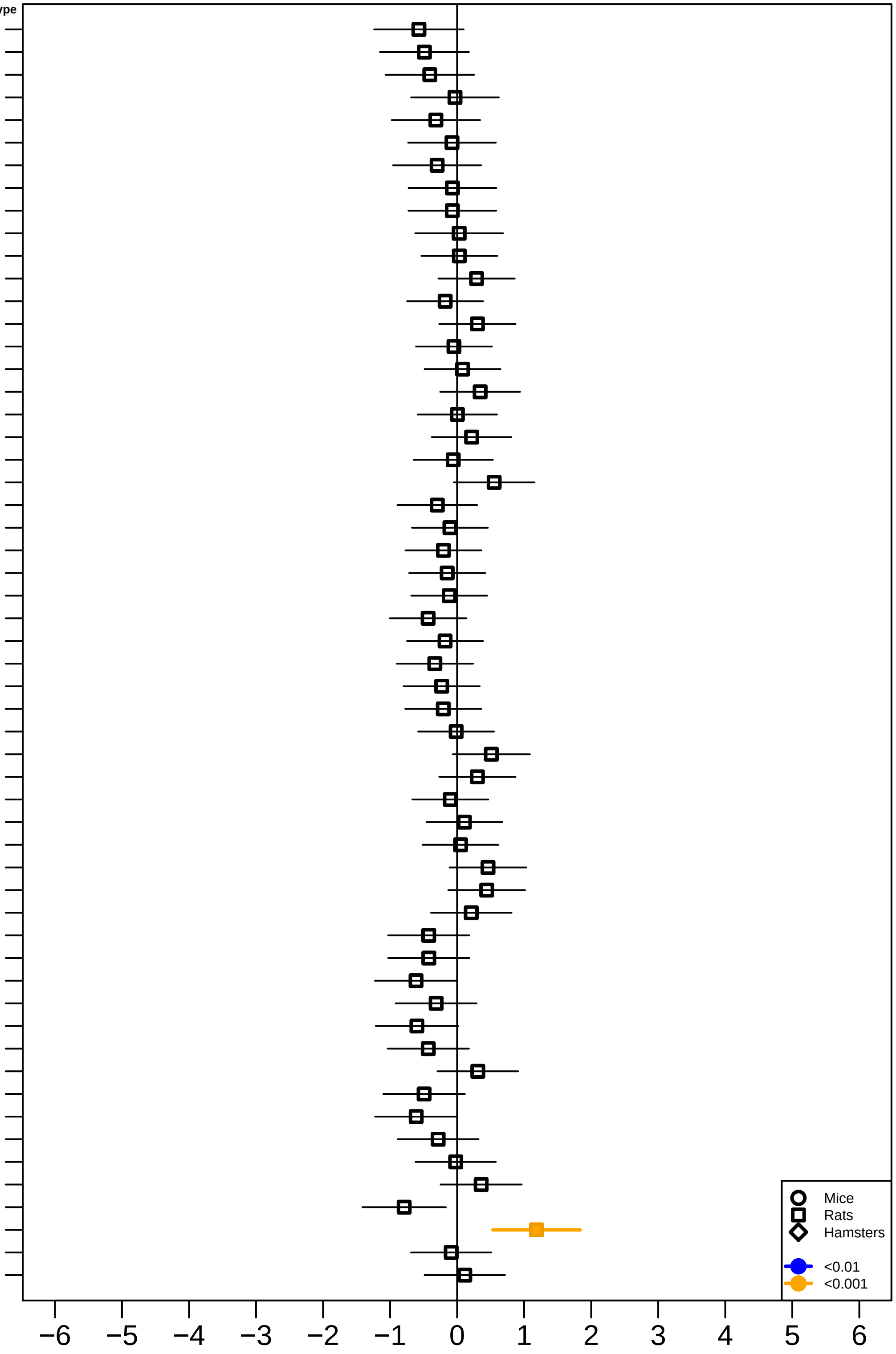

Figure S6 (Cont.)

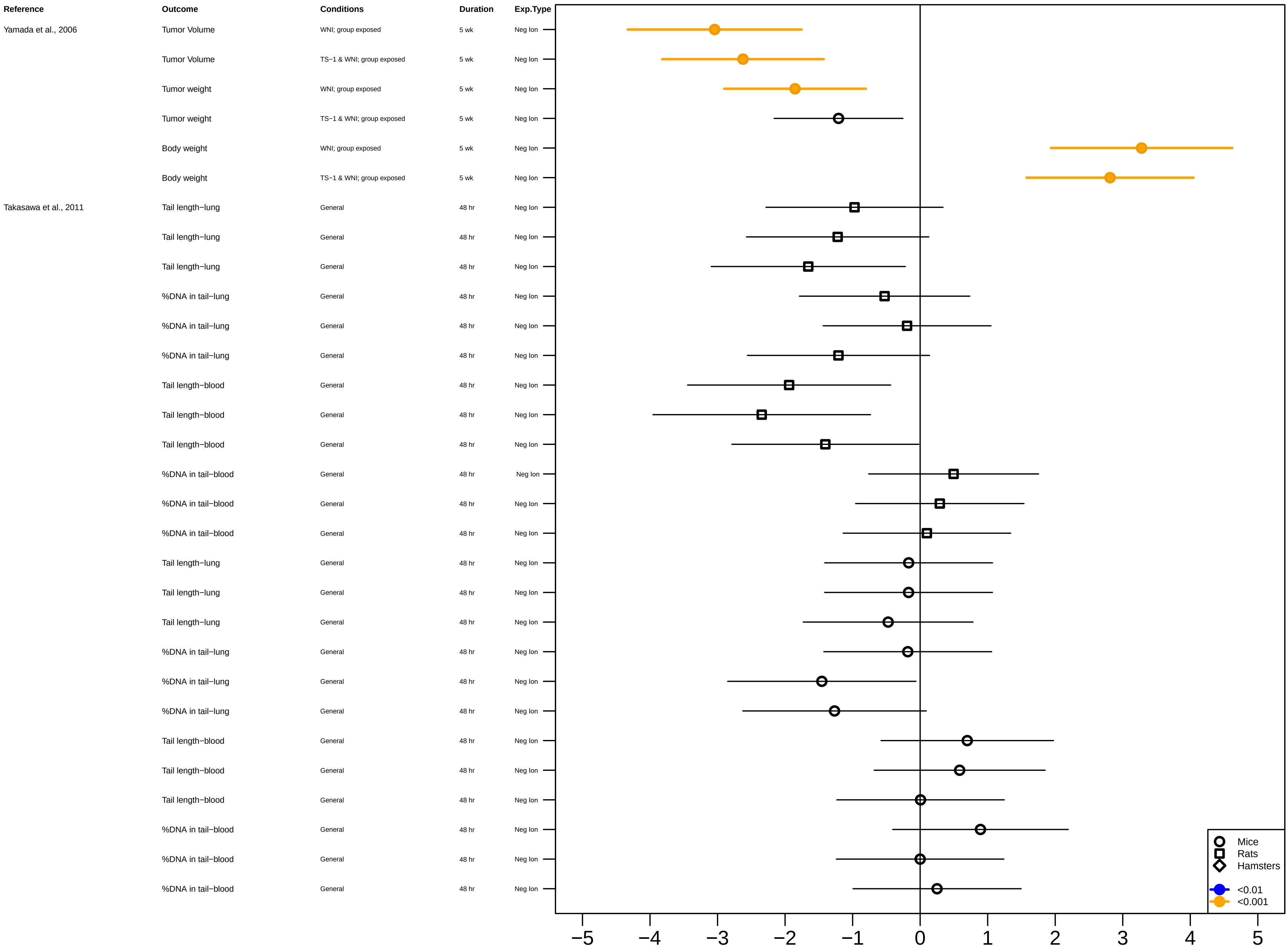

Figure S7. Standardized Mean Differences − Air Ion Carcinogenesis Studies

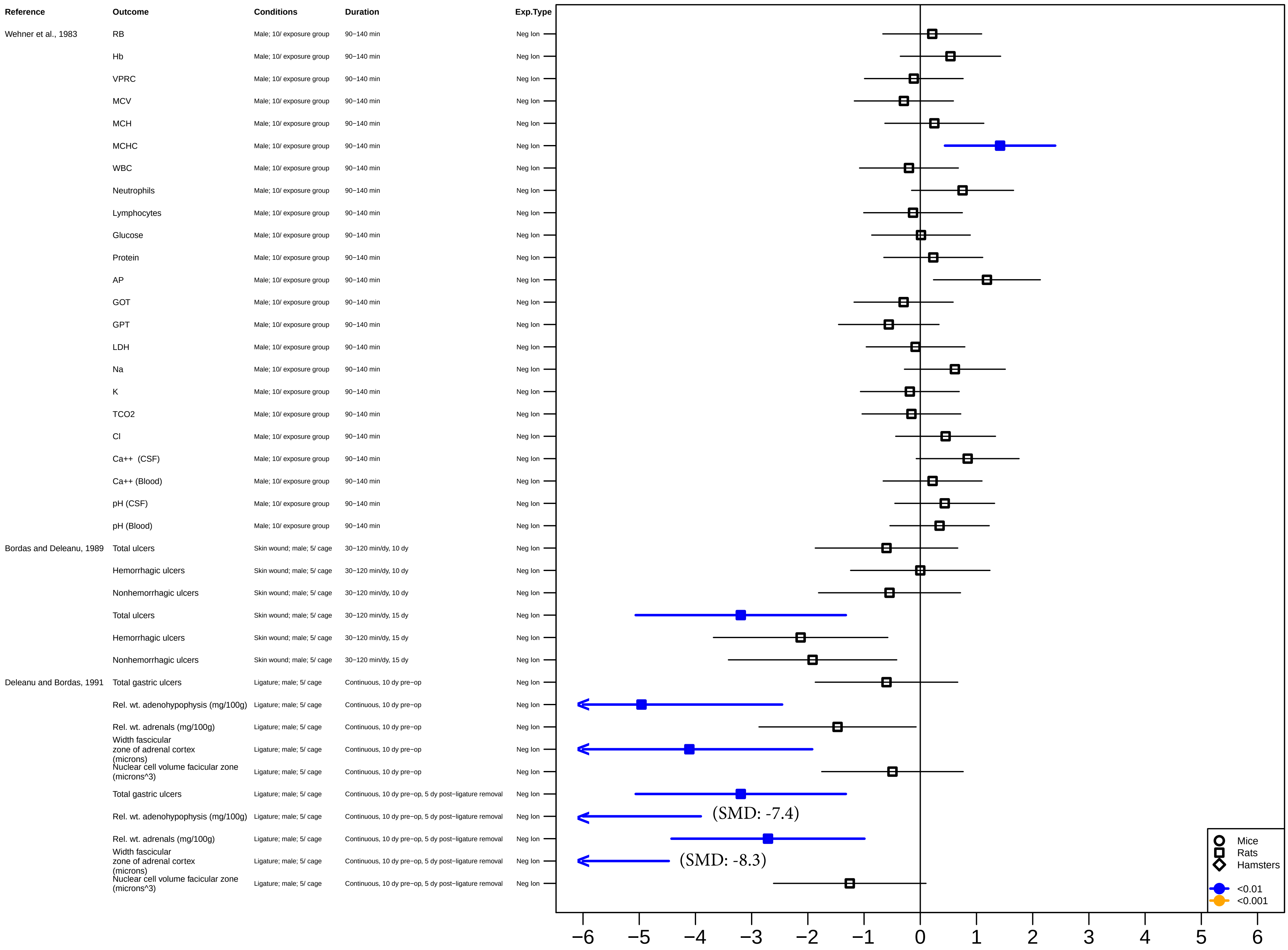

Figure S8. Standardized Mean Differences Air Ion − Other Health Endpoints Studies

Additional files 2: Abbreviations and notes

Figure S1 Standardized mean differences - air ion behavior studies

Abbreviations: dy, day; Hz, Hertz; hr, hours; kg, kilograms; mg, milligrams; min, minutes; neg, negative; pos, positive; wks, weeks. Excluded: SMDs could not be calculated for: Beardwood et al., 1987 [58]; Bailey and Charry, 1986 [57]; Creim et al., 1993 [59] and Livanova et al., 1999 [609].

Figure S2 Standardized mean differences - air ion learning and memory studies

Abbreviations: hrs, hours; ml, milliliter; min, minutes; neg, negative; pos, positive; wks, weeks. Excluded: SMDs could not be calculated for Bauer, 1955 [65]; Duffee and Koontz, 1965 [66]; Frey, 1967 [67]; Nazzario et al., 1967 [68]; Terry et al., 1969 [69]; and Falkenberg and Kirk, 1977 [70]. Data from Creim et al., 1995 [64] extracted from study figures.

Figure S3 Standardized mean differences - air ion serotonin or other neurotransmitter studies

Abbreviations: 5HT, 5-hydroxytryptamine (serotonin); 5HIAA, 5-hydroxyindoleacetic acid; AMP, adenosine 3', 5'-monophosphate; AMPT, d,1 a-methyl-p-tyrosine methyl ester; CI, confidence interval; CO2, carbon dioxide; GMP, guanosine 3', 5'-monophosphate; hr, hours; MFEC, multifamily enriched condition; N2, nitrogen; neg, negative; O2, oxygen; pos, positive; UFIC, unifamily impoverished condition. Data for calculation extracted from study figures in Krueger and Kotaka, 1969 [75]; Diamond et al., 1980 [76]; Dowdall and DeMontigny, 1985 [79].

Figure S4 Mean proportional differences - air ion respiratory infection studies

Abbreviations: dep, depleted of ions; hrs, hours; mix, mixture of pos and neg ions; neg, negative; pos, positive. Data for all studies were extracted from study figures.

Figure S5 Standardized mean differences - air ion cardiovascular function studies

Abbreviations: Amb, nucleus ambiguous; DBP, diastolic blood pressure; HF, high-frequency component in the power spectrum of HRV; HR, heart rate; LC, locus coeruleus; LFP, low-frequency power; MBP, mean blood pressure; neg, negative; pos, positive; PVN, paraventricular nucleus of the hypothalamus; SBP, systolic blood pressure. Data from Ju and Kubo 1997 [108]; Suzuki et al., 2008 [109] extracted from study figures.

Figure S6 Standardized mean differences - air ion reproduction and growth studies

Abbreviations: dy, day; hrs, hours; F0, initial breeding stock; F1, first generation; F2, second generation; neg, negative; ptrns, partitions; pos, positive; rm, room; subrm, subroom; wks, weeks, w/2, with 2. Excluded: SMDs could not be calculated for Herrington and Smith, 1935 [47]; Hinsull et al., 1981 [117]; Hinsull et al., 1983 [118].

Figure S7 Standardized mean differences - air ion carcinogenesis studies

Abbreviations: DNA, deoxyribonucleic acid; hr, hours; WNI, water-generated negative ions; neg, negative; pos, positive; TS-1, titanium silicate.

Figure S8 Standardized mean differences - other health endpoints studies

Abbreviations: Ca, calcium; CSF, cerebrospinal fluid; GOT, glutamic oxaloacetic transaminase; GPT, glutamic pyruvic transaminase; MC, mean corpuscular; MCH, mean corpuscular hemoglobin; min, minutes neg, negative; pH, potential of hydrogen; pos, positive; VPRC, volume of packed red blood cells. Excluded: SMDs could not be calculated for Jaśkowski and Myśliwski, 1986 [126].
