# Supplementary material for: Exposure of laboratory animals to small air ions: a systematic review of biological and behavioral studies
Source: Biomed Eng Online. 2018 Jun 5;17:72. doi: 10.1186/s12938-018-0499-z (PMC5987445; doi:10.1186/s12938-018-0499-z)
Supplement: Supplementary file 3 — Additional file 3. Evaluation of study quality and statistical strength of evidence. [file 12938_2018_499_MOESM3_ESM.pdf]

Additional File 3: Table S1 and Figures S2 and S3

Evaluation of Study Quality and Statistical Strength of Evidence

Table S1. Ratings of Individual Studies for Compliance with Quality Indicators and Statistical Strength of Evidence (number and percentage of tests reported as $p < 0.01$ and $p < 0.001$).

Figure S1. Comparison of Quality Ratings to Modest Statistical Strength of Evidence

Percent of Quality indicators (8 maximum) rated Yes for compliance in each study and the percent of SMDs and PDs reported at $p < 0.01$.

Figure S2. Comparison of Quality Ratings to Strong Statistical Strength of Evidence.

Percent of Quality indicators (8 maximum) rated Yes for compliance in each study and the percent of SMDs and PDs reported at $p < 0.001$.

Table S1. Ratings of Individual Studies* for Compliance with Quality Indicators and Statistical Strength of Evidence (Number of Tests Reported as p <0.01 and p <0.001)

**Study**	**Sham exposure**	**Confounding factors**				**Blinding**	**Randomized**	**Individual Exposure**	**Overall Compliance with Quality Indicators (percent)§**	**Statistical Strength of Evidence**	
		**Electric field**	**Ozone****	**Noise****	**Light****					p <0.01 / # of tests	p < 0.001 / # of tests
**Behavior**											
Herrington and Smith, 1935 [47]	**Y**	No	No	No	No	No	No	No	13	0/6	1/6
Bachman et al., 1966 [56]	**Y**	No	N/A	N/A	N/A	No	**Y**	No	40		
Olivereau and Lambert, 1981 [48]	**Y**	**Y**	**Y**	**Y**	No	No	No	No	50	0/2	0/2
Olivereau et al., 1981 [49]	**Y**	No	**Y**	No	No	**Y**	No	No	38	2/12	1/2
Lambert et al., 1981 [50]	**Y**	No	**Y**	No	No	**Y**	No	**Y**	50	2/18	1/18
Beardwood et al., 1986, 1987 [53, 58]	**Y**	No	No	No	No	No	No	No	13		

Bailey and Charry, 1986 [78]	**Y**	**Y**	**Y**	**Y**	No	**Y**	**Y**	**Y**	88		
Lambert and Olivereau, 1987 [55]	**Y**	No	**Y**	No	No	**Y**	**Y**	**Y**	63	0/12	1/12
Lenkiewicz et al., 1989 [52]	**Y**	No	No	No	No	No	No	**Y**	25	1/6	2/6
Beardwood and Jordi, 1990 [54], Beardwood et al. 1987 [58]	**Y**	No	No	No	No	No	**Y**	No	25	0/18	0/18
Dabrowska et al., 1990 [51]	**Y**	No	No	No	No	No	No	**Y**	25	4/32	2/32
Creim et al., 1993 [59]	**Y**	**Y**	**Y**	**Y**	**Y**	No	No	**Y**	75		
Livanova et al., 1999 [60]	No	No	No	No	No	No	No	No	0		
**Learning & Memory**											
Bauer, 1955 [65]	No	No	N/A	N/A	N/A	No	No	No	0		
Jordan and Sokoloff, 1959 [62]	No	No	N/A	N/A	N/A	No	No	No	0	0/4	2/4
Duffee and Koontz, 1965 [66]	No	No	N/A	N/A	N/A	**Y**	**Y**	No	40		
Frey, 1967 [67]	No	No	No	No	No	No	**Y**	No	60		

Nazzaro et al., 1967 [68]	**Y**	No	N/A	N/A	N/A	No	No	**Y**	40		
Terry et al., 1969 [69]	**Y**	No	N/A	N/A	N/A	No	**Y**	No	40		
Falkenberg and Kirk, 1977 [70]	No	No	No	No	No	No	**Y**	**Y**	25		
Olivereau and Lambert, 1981 [48]	**Y**	**Y**	**Y**	No	No	No	No	No	38	0/14	0/14
Creim et al., 1995 [64]	**Y**	**Y**	**Y**	**Y**	**Y**	N	**Y**	**Y**	88	0/10	0/10
**Serotonin**											
Krueger and Smith, 1960 [71]	**Y**	No	N/A	N/A	N/A	No	No	**Y**	40	1/2	1/2
Krueger et al., 1963 [72]	No	No	N/A	N/A	N/A	No	**Y**	No	20	2/2	2/2
Krueger et al., 1966 [73]	No	No	N/A	N/A	N/A	No	**Y**	No	20	0/3	0/3
Krueger et al., 1968 [74]	No	No	N/A	N/A	N/A	No	No	No	0	0/5	0/5
Krueger and Kotaka, 1969 [75]	No	**Y**	N/A	N/A	N/A	No	**Y**	No	40	3/24	0/24
Gilbert, 1973 [76]	No	No	N/A	N/A	N/A	No	**Y**	**Y**	40	0/2	0/2
Diamond et al., 1980	No	No	No	No	No	**Y**	No	No	13	0/12	1/12

[76]											
Charry and Bailey, 1985 [82]	**Y**	**Y**	**Y**	**Y**	No	**Y**	**Y**	**Y**	88	0/100	0/100
Dowdall and DeMontigny, 1985 [79]	**Y**	No	No	No	No	**Y**	No	No	25	0/12	0/12
Kellogg et al., 1985 [80]	**Y**	**Y**	N/A	N/A	N/A	No	No	No	40	0/4	0/4
Kellogg et al., 1985 [81]	**Y**	**Y**	N/A	N/A	N/A	No	No	No	40	0/4	0/4
Beardwood et al., 1987 [58]	No	No	No	No	No	No	No	No	0	1/4	0/4
Bailey and Charry, 1987 [57]	**Y**	**Y**	**Y**	**Y**	No	**Y**	**Y**	**Y**	88	0/90	0/90
**Tracheal**											
Krueger and Smith, 1958 [86]	**Y**	No	N/A	N/A	N/A	No	No	**Y**	40		
Krueger and Smith, 1958 [87]	**Y**	N	N/A	N/A	N/A	No	No	**Y**	40		
Krueger and Smith, 1959 [88]	**Y**	No	N/A	N/A	N/A	No	No	No	20		
Krueger and Smith, 1960 [71]	**Y**	No	N/A	N/A	N/A	No	No	No	20		
Andersen, 1972	**Y**	**Y**	N/A	N/A	N/A	No	No	**Y**	60		

[94]											
Sirota et al., 2006 [97]	**Y**	No	**Y**	No	No	No	No	No	25		
Sirota et al., 2008 [98]	No	No	No	No	No	No	No	No	0		
**Respiratory Infection**											
Krueger and Levine, 1967 [99]	**Y**	**Y**	N/A	N/A	N/A	No	**Y**	No	60	0/2	0/2
Krueger et al., 1970 [100]	**Y**	No	N/A	N/A	N/A	No	**Y**	No	40	1/4	2/4
Krueger et al., 1971 [101]	No	No	N/A	N/A	N/A	No	No	No	0	0/1	0/1
Krueger and Reed, 1972 [102]	**Y**	No	No	No	No	No	No	No	13	4/10	3/10
Krueger et al., 1974 [103]	**Y**	**Y**	N/A	N/A	N/A	No	No	No	40	0/4	0/4
**Cardiovascular Function**											
Bachman et al, 1965 [106]	**Y**	No	N/A	N/A	N/A	No	No	Y	40	1/4	0/4
McDonald et al., 1965 [107]	**Y**	No	N/A	N/A	N/A	No	No	**Y**	40	0/4	0/4
Bachman et al, 1966 [110],	**Y**	No	N/A	N/A	N/A	No	No	**Y**	45		

Ju and Kubo, 1997 [108]	No	N/A	N/A	N/A	N/A	No	No	**Y**	20	0/5	0/4
Suzuki et al., 2008 [109]	**Y**	No	N/A	No	N/A	No	No	**Y**	33	1/12	0/12
**Reproduction and Growth**											
Herrington and Smith, 1935 [47]	**Y**	No	No	No	No	No	No	**Y**	25		
Hinsull et al., 1981 [117]	No	No	No	No	No	No	**Y**	No	13		
Hinsull et al., 1983 [118]	No	No	No	No	No	No	No	No	0		
Hinsull et al., 1984 [111]	No	No	No	No	No	No	No	No	0	0/4	0/4
Kellogg et al., 1985 [80]	**Y**	**Y**	N/A	N/A	N/A	No	No	No	40	3/20	1/20
Kellogg et al., 1985 [81]	**Y**	**Y**	N/A	N/A	N/A	No	No	No	40	0/16	0/16
Kellogg and Yost, 1986 [114]	**Y**	**Y**	N/A	N/A	N/A	No	No	No	40	0/1	1/1
Hinsull and Head, 1986 [113]	**Y**	No	No	No	No	No	No	No	13	1/2	1/2
Hinsull, 1988 [112]	**Y**	No	No	No	No	No	No	No	13	0/2	0/2

Yamamoto et al., 2014 [115]	**Y**	No	No	No	No	No	**Y**	No	25	0/7	0/7
Yamamoto et al., 2015 [116]	**Y**	No	No	No	No	No	**Y**	No	25	2/224	4/224
**Carcinogenesis**											
Yamada et al., 2006 [119]	No	No	No	No	No	No	No	No	0	0/6	5/6
Takasawa et al., 2011 [120]	**Y**	No	No	No	No	No	**Y**	**Y**	38	0/24	0/24
**Other health endpoints**											
Wehner et al., 1983 [123]	**Y**	No	No	No	N/A	No	**Y**	No	29	1/23	0/23
Jaśkowski and Myśliwski, 1986 [126]	**Y**	No	No	No	No	No	No	**Y**	25		
Bordas and Deleanu, 1989 [124]	No	No	No	No	No	No	No	No	0	1/6	0/6
Deleanu and Bordas, 1991 [125]	No	No	**Y**	No	No	No	No	No	13	6/10	0/10

* Compliance is indicated by Y (Yes); non-compliance by No; N/A (Not Applicable)

** For air ion exposures produced by non-corona sources these confounders were assumed to be de minimus and therefore rated as Not Applicable (N/A)

§ N/A ratings are not included in the maximum number of rating for the calculation of percent compliance

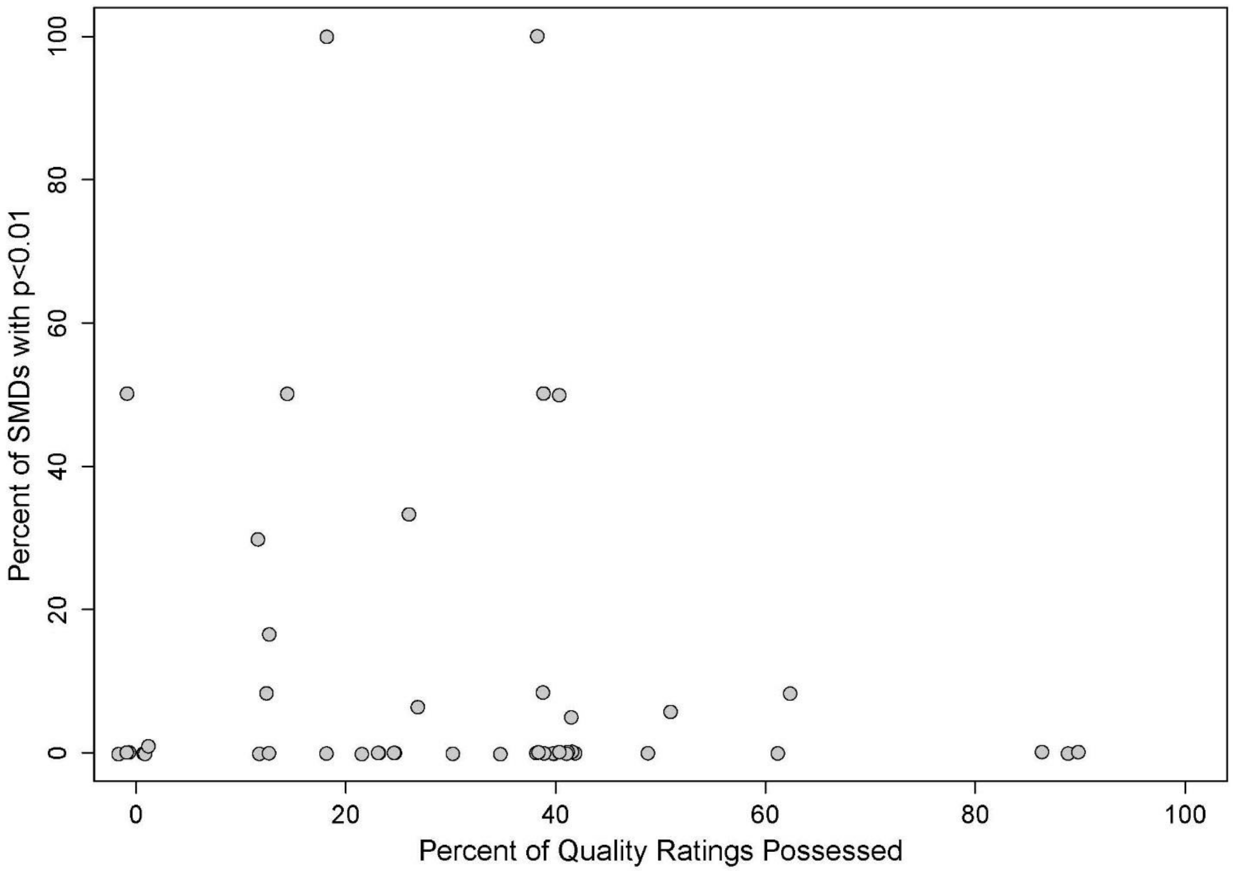

Figure S1. Comparison of Quality Ratings to Modest Statistical Strength of Evidence.

Percent of quality indicators (8 maximum) rated Yes for compliance in each study and the percent of SMDs and PDs reported at *p* <0.01.

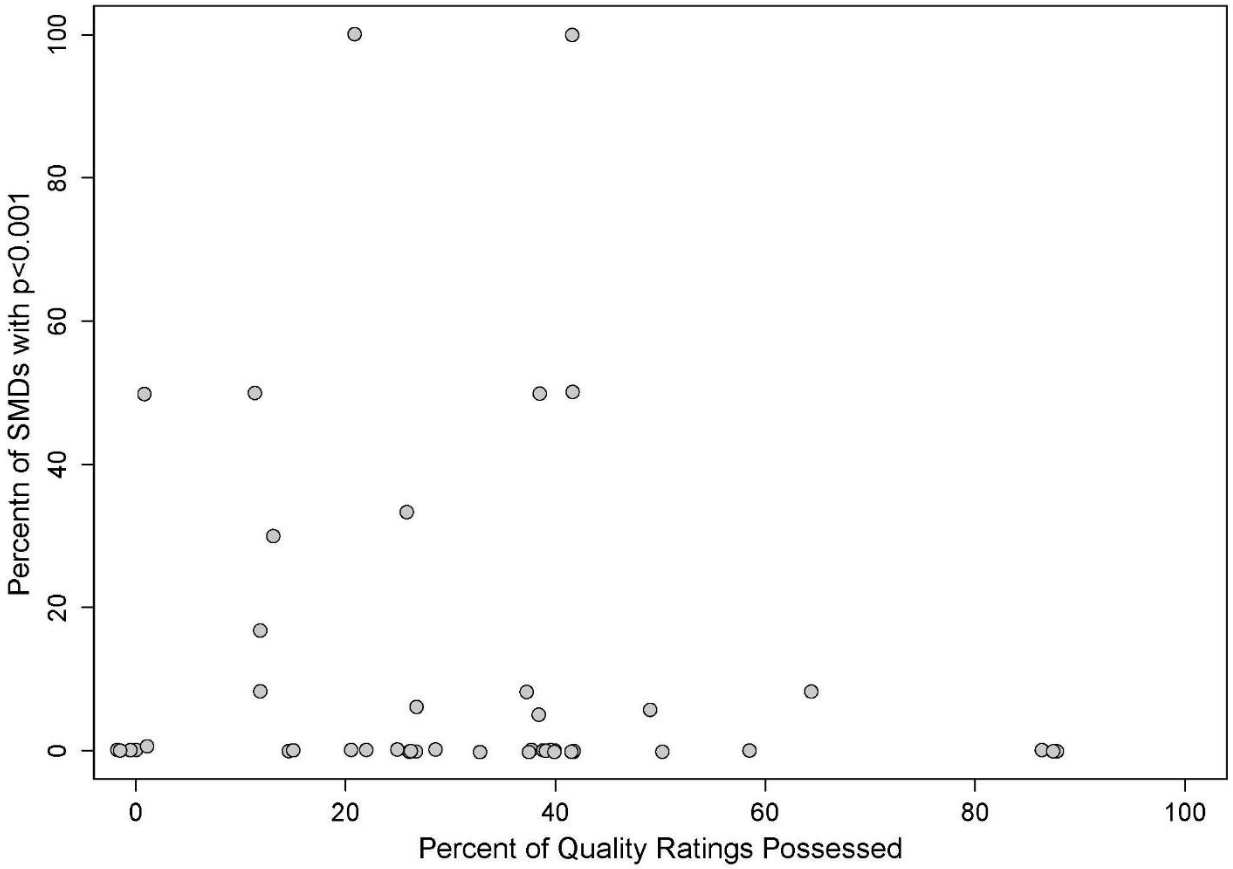

Figure S2. Comparison of Quality Ratings to Strong Statistical Strength of Evidence.

Percent of quality indicators (8 maximum) rated Yes for compliance in each study and the percent of SMDs and PDs reported at $p < 0.001$.
